# Folding complex DNA nanostructures from limited sets of reusable sequences

**DOI:** 10.1093/nar/gkw208

**Published:** 2016-04-01

**Authors:** Stefan Niekamp, Katy Blumer, Parsa M. Nafisi, Kathy Tsui, John Garbutt, Shawn M. Douglas

**Affiliations:** Department of Cellular and Molecular Pharmacology, University of California, San Francisco, CA 94158, USA

## Abstract

Scalable production of DNA nanostructures remains a substantial obstacle to realizing new applications of DNA nanotechnology. Typical DNA nanostructures comprise hundreds of DNA oligonucleotide strands, where each unique strand requires a separate synthesis step. New design methods that reduce the strand count for a given shape while maintaining overall size and complexity would be highly beneficial for efficiently producing DNA nanostructures. Here, we report a method for folding a custom template strand by binding individual staple sequences to multiple locations on the template. We built several nanostructures for well-controlled testing of various design rules, and demonstrate folding of a 6-kb template by as few as 10 unique strand sequences binding to 10 ± 2 locations on the template strand.

## INTRODUCTION

DNA nanotechnology solves an important problem that remains extremely challenging for other engineering platforms, which is the positional control of matter on nanometer length scales (1). Thus, DNA may hold great potential for creating nanoscale tools and devices that could impact many fields including materials science, electronics, and medicine. However, the path from laboratory proofs-of-concept to demand-meeting applications will require further innovation in both design and synthesis of DNA nanostructures.

When developing novel strategies for creation of DNA nanostructures, we can evaluate design choices in the context of how the structure will be used and how it will be made. We might consider the total amount of structures needed, ease of design, initial and marginal costs of synthesis and recovery, minimum yield of well-folded structures, surface addressability and so on. Certain properties that appear meaningful in one context may be less relevant in another context.

Once functional requirements are chosen, many design parameters can be explored such as tiled (2–4) versus templated (5,6) assembly, crossover arrangement (7), helix-axis orientation (8–11), total size in nucleotides (12) and multimerization via sticky ends (13) or base stacking (14). For example, many studies have made a point of exploring shape diversity by fixing some of these parameters and varying others. Using tiled assembly, hundreds of shapes have been created by fixing the sequences and orientation of a set of strands, and varying which subset of strands are folded. Using a templated approach, the similar scaffold sequences have been folded into 2D planar (6), 3D planar (15,16), 3D lattice (17,18), gridiron (10) and polyhedral mesh (11) shapes by varying helix-axis orientation and crossover arrangements.

**Figure 1.**
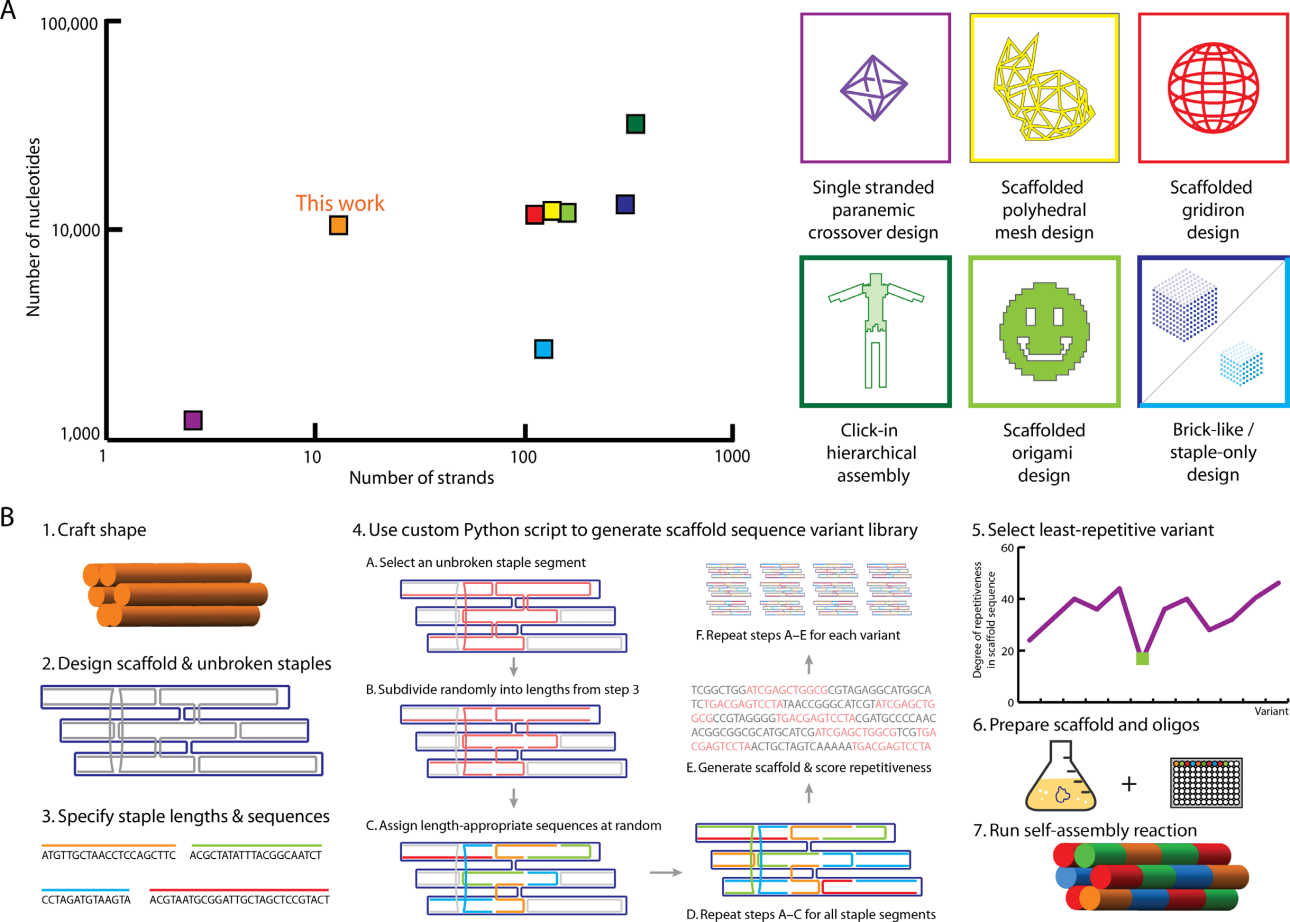
Custom scaffolded DNA nanostructures in context of previous works. (**A**) 2D plot of number of strands versus number of nucleotides for representative shapes folded using selected DNA nanostructure design strategies: Single-stranded paranemic crossover design (5) (purple), scaffolded polyhedral mesh design (11) (yellow), scaffolded gridiron design (10) (red), click-in hierarchical assembly (14) (dark green), scaffolded origami design (6) (light green), staple-only (4) design (light and dark blue), and custom scaffold DNA origami design (orange). (**B**) Step-by-step guide to design and fold custom scaffold DNA origami structures. Step 1: Conceive a target shape. Step 2: Lay out scaffold and unbroken staples accordingly using caDNAno. Step 3: Define number of unique staple sequences to use and either provide staple sequences to custom Python script or use script to generate random staple sequences. Steps 4: Apply custom Python script to caDNAno design to generate a library of layouts. Step 5: Select staple layout with the least repetitive scaffold sequence. Step 6: Clone custom scaffold into M13K07 vector for amplification and isolate custom scaffold. Step 7: Mix all components (scaffold, staples and buffer) and run molecular self-assembly reaction by thermal annealing.

Here, we aimed to take a step toward applications of DNA nanotechnology that require large-scale synthesis of complex structures comprising at least 10,000 nucleotides. The future scalability of templated structures appears promising in light of recently reported gram-scale production of single-stranded DNA (ssDNA) scaffold templates in bioreactors (19). However, because each unique template-binding ‘staple’ strand requires a separate synthesis step, large-scale synthesis remains prohibitively expensive for methods that rely on hundreds of unique strands (cost calculations in Supplementary Data). Thus, we sought to reduce the total number of distinct strands necessary to fold a structure (Figure 1A). We report a novel approach to creating DNA nanostructures that reduces the marginal cost of large-scale strand synthesis by reusing individual staple sequences multiple times on the same template. Like Shih-style single-stranded DNA origami (5), our approach comes with some tradeoffs compared to other methods, namely the initial difficulty and costs associated with strand and template design are increased. Nevertheless, by employing both custom scaffolds and templated assembly, we were able to expand the available design space for parameters such as template length, sequence content and number of strands. That flexibility allowed us to achieve an order-of-magnitude reduction in the number of unique strands required to fold DNA nanostructures (Supplementary Figure S1) without significant reductions in size, complexity or yield.

Designing DNA nanostructures using our method requires some modifications to similar template-based design approaches (Figure 1B). Using caDNAno, a computer-aided design tool for DNA origami (20), we first routed the scaffold to approximate a 3D shape and exported the design from caDNAno as a text file in JSON format. Next, we input the JSON file into a custom Python script (see Materials and Methods) and specified the desired number of unique staples (e.g. (10)). The script determines each custom scaffold sequence by generating a random staple layout, repeatedly assigning a set of staple sequences to that layout, and then assigning the appropriate complementary bases to the scaffold. Because highly repetitive DNA sequences can be difficult to synthesize, we sought to minimize scaffold sequence repetitiveness. We created a library of candidate scaffold sequences and selected the top-ranked sequence according to total fraction of nucleotides that appear in a ‘repetitive’ motif, defined as a 12-base window that appears more than once in the scaffold. Using a similar approach to the MOSIC (21) method for enzyme-mediated production of DNA oligonucleotides, we cloned each scaffold sequence flanked by hairpins encoding recognition sequences for the restriction enzyme BtsCI into the helper phage M13K07 for amplification of ssDNA (Supplementary Figure S2). We purified ssDNA (22) followed by a digestion with BtsCI for separation of the vector M13K07 and the custom scaffold. The scaffold was then added to folding reactions to create the final shapes.

## MATERIALS AND METHODS

### Design of custom scaffolds

The script and a detailed manual are available for download here: https://github.com/stefanniekamp/ReuseDNA. In brief, the parameters that can be changed besides the number of staples are: Input caDNAno / json-file, number of iterations that should be used (number of different versions that will be generated), number of best design(s) in terms of degree of repetitiveness in scaffold sequence ranked from lowest to highest that should be shown, usage of predefined staple sequences or random sequences, the minimum repeat length for repetitive motifs and staple lengths as well as colors. The output will be as depicted in Figure 1B where the degree of repetitiveness for each scaffold sequence is shown. An example output is also shown in Supplementary Figure S3. In addition to the plot caDNAno / json-files and sequences for all scaffolds are generated and saved.

### Cloning of custom scaffolds

Screening device custom scaffold sequence inserts (1082 bp) were ordered in pBluescript from Genewiz (sequences can be found in Supplementary Data). They were then PCR amplified with ctagta**ccgcgg**AGGAATAGGGC and ctagta**gagctc**GTCGACCCACTC (uppercase bases anneal with scaffold sequence in pBluescript and lowercase bases add SacII and SacI restriction sites with some extra DNA to allow the enzyme to bind, respectively). The amplicon was digested and cloned into M13K07 RF at SacII and SacI restriction sites. Larger custom scaffold sequences for the 24-helix bundles were ordered as DNA blocks from Genewiz and Gibson-cloned into digested (SacII and SacI) M13K07 RF. For Gibson cloning of each of the three designs, three ∼2 kb DNA blocks were assembled (see block sequences in Supplementary Data) and amplified (primer sequences in Supplementary Table S2) before cloning into M13K07 RF.

### Scaffold amplification and purification

First, a 5 mL overnight culture of XL1-Blue cells (if not specified otherwise) in LB media with tetracycline was grown. The next day a 500 mL flask with 150 mL of 2xYT medium mixed with 1.5 mL of the saturated overnight culture, 1 mL of 50% glucose, 1.5 mL of 1.3 M MgCl_2_, tetracycline, and kanamycin was prepared. Then, the culture was incubated at 37°C with shaking at 250 rpm for ∼6 h before the supernatant was harvested by spinning at 4,000 rcf for 15 min at 4°C. Afterwards, the supernatant was filtered with four layers of Whatman No. 1, followed by a 0.6 μm glass fiber filter, and a 0.2 μm filter. Then, PEG 8000 and NaCl were added to final concentrations of 40 and 30 g/L, respectively. Samples were incubated in an ice bath for 30 min. Next, the phage was pelleted at 4,000 rcf for 15 min at 4°C and the supernatant was decanted. Then, the pellet was resuspended in 1/100 of the original culture volume in TE buffer (5 mM Tris pH 8.5 and 1 mM EDTA). Afterwards, residual *E. coli* cells were pelleted at 15,000 rcf for 15 min at 4°C and phage supernatant was transferred to a fresh container. This was followed by the addition of 2 volumes of lysis buffer (0.2 M NaOH, 1% SDS) and 1.5 volumes of neutralization buffer (3 M KOAc pH 5.5). The mixture was incubated in an ice-water bath for 15 min, and then spun at 16,000 rcf for 15 min at 4°C. Next, the supernatant was transferred into fresh centrifuge bottles. Immediately, 2 volumes of ice cold 100% ethanol were added and mixed by swirling. The mixture was incubated in a 20°C freezer for 2 h and spun at 16,000 rcf for 15 min at 4°C afterwards. Next, the supernatant was decanted and 10 mL of ice cold 75% ethanol was added to each centrifuge bottle and mixed by swirling. Afterwards, the mixture was spun at 16,000 rcf for 5 min at 4°C and the supernatant was removed. Finally, the pellet was air dried and resuspended in TE buffer (5 mM Tris pH 8.5 and 1 mM EDTA) - the volume will depend on desired final concentration of scaffold. For the custom scaffold 24-helix bundle, the purified scaffold was subsequently digested with BtsCI (NEB, Catalog # R0647L) as follows: 10 μL of ssDNA at 100 nM with 2 μL of 10x CutSmart Buffer, 1 μL of BtsCI and 7 μL of ddH_2_O was incubated at 50°C overnight. Note, that the final scaffold sequence will contain two dsDNA restriction sites for BtsCI (hairpin at each end, Supplementary Figure S2) and several ssDNA restriction sites for BtsCI. But since BtsCI has a significantly higher affinity for dsDNA, this was not a concern.

### Oligonucleotides

All oligonucleotides were ordered from IDT and resuspended in 5 mM Tris pH 8.5 and 1 mM EDTA.

### Molecular self-assembly reactions and purification

Scaffold (final concentration 20 nM) and staples (final concentration 200 nM for DNA origami, which equals a 10-fold excess per corresponding scaffold binding site, and a 10-fold excess per corresponding scaffold binding sites for custom scaffold DNA origami was used, if not specified otherwise), were mixed in 5 mM Tris pH 8.5, 1 mM EDTA and 18 mM MgCl_2_ and annealed with the following temperature ramp: denaturation at 65°C for 15 min followed by cooling from 62°C to 35°C with a decrease of 1°C per 2 h. Then, the reaction was held at 12°C for at least 30 min. Afterwards, products were analyzed by 2% agarose gel electrophoresis in TBE (45 mM Tris-borate and 1 mM EDTA) with 11 mM MgCl_2_ and purified by extraction and centrifugation in Freeze’ N Squeeze columns.

### Agarose gel-based yield estimation

Agarose gel-based yield estimation was carried out by using ImageJ (http://rsb.info.nih.gov/ij/). The percentage of structure that ran as a monomeric, leading band was estimated as the background-subtracted integrated intensity value divided by the background-subtracted integrated intensity value enclosing the material from the well, down to the bottom of the leading band.

### Transmission electron microscopy

Six microliters of the purified folding reaction product was applied on glow-discharged, carbon-coated, 400 mesh formvar grids (Electron Microscopy Sciences), incubated for 1 min, blotted off and stained with 2% (w/v) aqueous uranyl formate solution. The electron micrographs were collected with a FEI TECNAI T20 transmission electron microscope and a Tietz TVIPS 8k camera at normal magnification of 46,000×. Particles for class averaging were picked and calculated with EMAN 2. The number of particles picked for the class averages in Figure 3A, B and C was between 220 and 295.

## RESULTS AND DISCUSSION

**Figure 2.**
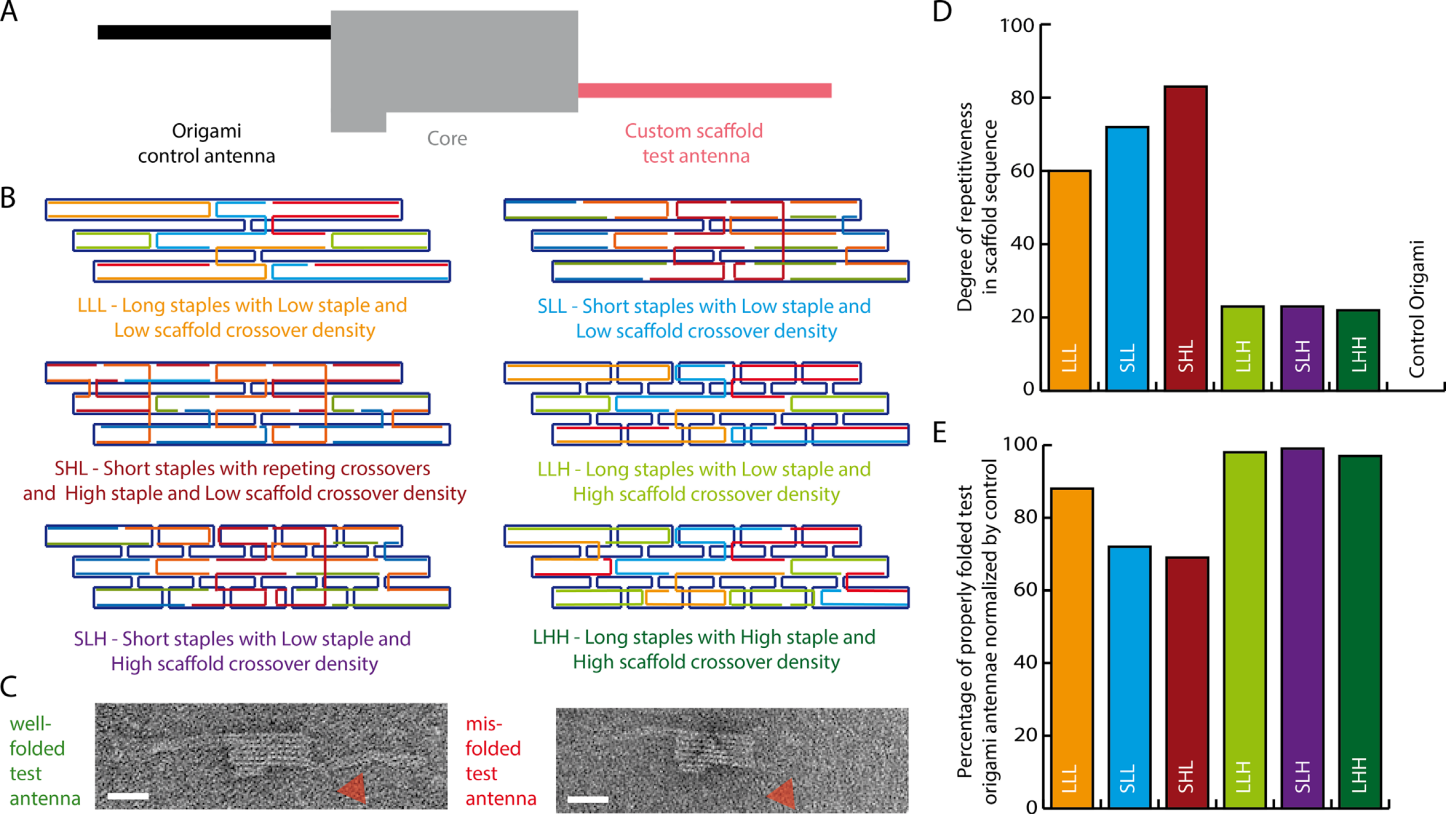
DNA origami screening device enables side-by-side testing of control and custom-scaffolded antenna domains. (**A**) Schematic of screening device. An asymmetric central core (gray) allows for distinguishing between control (black) and test domains (red). The control antennae are designed with the standard DNA origami method and the test antennae with custom scaffold sequences and reused staple sequences. (**B**) Six versions of custom scaffold antennae where the scaffold is shown in dark blue and like-colored staples represent identical sequences: (i) LLL: Long staples with Low staple and Low scaffold crossover density, (ii) SLL: Short staples with Low staple and Low scaffold crossover density, (iii) SHL: Short staples with repetitive staple crossover arrangement, and High staple and Low scaffold crossover density, (iv) LLH: Long staples with Low staple and High scaffold crossover density, (v) SLH: Short staples with Low staple and High scaffold crossover density, and (vi) LHH: Long staples with High staple and High scaffold crossover density. (**C**) Representative transmission electron microscope (TEM) micrographs of well-folded (left) and misfolded (right) test antennae (red arrowhead). Scale bars: 50 nm. (**D**) Comparison of degree of repetitiveness in scaffold sequence in antenna of the six approaches shown in (B) and the origami control. (**E**) Relative folding yields of the six antenna types, normalized by the folding yield of the corresponding control antenna. The number of particles analyzed for each design was between 110 and 202.

Adapting a template-based design strategy for reduced strand counts without reducing the nucleotide count required the introduction of repetitive elements into our custom scaffold sequences. To assess the feasibility of our approach and to examine some initial design parameters, we created a DNA origami screening device with a central core domain and two opposing ‘antennae’ (Figure 2). Each antenna is a six-helix bundle folded from a 1082-base scaffold segment. The antennae can be distinguished by an asymmetric domain in the core structure (Figure 2A). A series of ‘control’ and ‘test’ antenna pairs were designed (Supplementary Figure S4). Various control antennae were designed using the standard DNA origami method with a fully unique staple set. We cloned several custom scaffold segments at the location of the second antenna for testing.

We analyzed the influence of four parameters on custom sequence repetitiveness and antennae folding yield: scaffold crossover density, staple crossover density, staple length, and repetitiveness of staple crossover arrangements. We again scored repetitiveness using a 12-base window. Crossover densities were ranked by counting the number of crossovers per 1,000 nucleotides. We analyzed designs with ‘short’ staples (25–62 bases) and ‘long’ staples (60–125 bases), listed in Supplementary Table S1. We also tested one design with highly repetitive staple crossover arrangements. That is, when the same staple binds to the scaffold in different locations, the crossover positions tend to occur at identical phosphate positions within the staple (Supplementary Figure S4).

We tested three parameters across six designs, and devised a three-letter abbreviation to identify each parameter set (Short or Long staple length, Low or High staple crossover density, and Low or High scaffold crossover density). Thus our six designs can be designated as LLL, SLL, SHL, LLH, SLH and LHH (Figure 2B and Supplementary Figure S5 and Table S1). After running the folding products on an agarose gel, we isolated the leading bands for all six designs by physical extraction (Supplementary Figure S6) and determined relative yields of antenna domains by manually counting the percentage of well-folded custom scaffold antennae as visualized by negative-stain transmission electron microscopy. We normalized yields by the percentage of well-folded control antennae attached to the same DNA origami screening devices (Figure 2C and Supplementary Figure S7). Of the six versions, we observed that the three least-repetitive designs folded with the highest yields ranging from 96% to 98%. The three most-repetitive designs folded to lower yields ranging from 69% to 88% (Figure 2D and E). Hence, there seems to be an inverse correlation between the yield of correctly folded antennae and the degree of repetitiveness in the scaffold sequence. Comparing the SHL and SLH designs, which have short staples and a similar combined number of staple and scaffold crossovers (Supplementary Table S1), we observed that the SHL design with its repetitive staple crossover arrangement has a much higher sequence redundancy and lower yield. This may indicate that repetitive staple crossover arrangements can compromise folding yield. When we compare SLL and LLL designs, it appears that using longer staples improved the yield, perhaps due to the lower degree of repetitiveness in the scaffold sequence. In light of these data, we designed subsequent shapes using a high density of staple and scaffold crossovers, longer staples and non-repetitive staple crossover arrangements.

**Figure 3.**
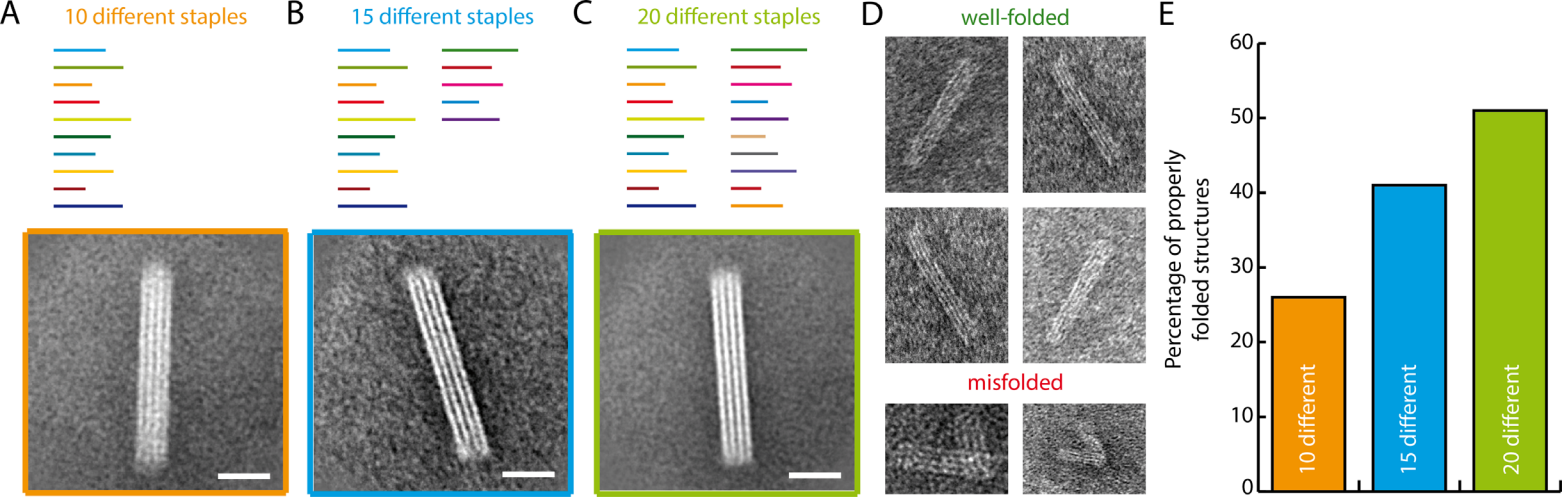
DNA nanostructures folded with custom scaffolds using 10, 15 or 20 unique staple sequences binding in multiple template locations. TEM class averages of 24-helix bundles designed with (**A**) 10 unique staples that each bind 10±2 template locations, (**B**) 15 unique staples that each bind in 7 ± 1 template locations and (**C**) 20 unique staples that each bind 5 ± 1 template locations. Scale bars: 20 nm. (**D**) TEM micrographs of representative well-folded (top) and misfolded (bottom) 24-helix bundle particles. (**E**) Absolute folding yields for designs with 10 (orange), 15 (blue) and 20 (green) unique staple sequences, as determined by gel electrophoresis and by manual counting of 142–355 particles in electron micrographs for each design.

We next set out to determine how few unique staple sequences could be used to fold a large (>10,000 nucleotide) DNA nanostructure without significantly compromising the folding yield (Figure 3). We designed a set of 24-helix bundles with 6-kilobase scaffolds, and tested versions designed to fold using 10, 15 or 20 unique staple sequences (Figure 3A, B, C, respectively). For comparison, a similar shape designed using the DNA origami method requires approximately 150 unique staples. Thus, for the designs with 10, 15 and 20 different staple sequences that means a reduction in number of different strands of 15-, 10- and 7.5-fold, respectively. We generated scaffold sequences with 56% to 39% repetitiveness using a 12-base window (Supplementary Figures S8 and S9). For comparison, the standard DNA origami scaffold M13mp18 contains only 2% sequence redundancy by this measure. We followed the design strategy described above, allowing the script to generate staple sequences with lengths ranging from 38–77 bases and a crossover density of 240 crossovers per 1,000 nucleotides. We successfully folded all three versions as can be seen by the transmission electron micrograph class averages (Figure 3A–C) and representative micrographs of individual particles (Figure 3D and Supplementary Figure S10).

The fraction of structures that migrated as a monomeric species in gel electrophoresis was estimated as integrated intensity of the leading band divided by total intensity of gel lane up to and including the well (Supplementary Figure S11). Here, we found 48%, 59% and 61% of intensity in the leading bands for the designs with 10, 15 and 20 different staple sequences, respectively. Subsequently, the yield estimate was refined by manually counting the percentage of well-folded particles from purified structures as seen in electron micrographs. For the designs with 10, 15 and 20 different staple sequences we counted 55%, 69% and 83% intact structures, and thus calculated absolute yields of 26%, 41% and 51%, respectively (Figure 3E). We observed an inverse correlation between the yield of intact structures and the repetitiveness in the scaffold sequence.

Finally, we quantified the impact of staple-to-scaffold concentration on folding yield of our designs. We folded structures with 2-, 3-, 4-, 5-, 6-, 8- and 10-to-1 ratios of staple-to-scaffold binding sites and measured the folding yield by gel electrophoresis (Supplementary Figure S12). This study was carried out with the 24-helix bundle with 10 unique staple sequences. We noted relative similar yields (21–22%) for the folding reactions with 2- and 3-fold staple excess, higher yields (33-42%) for the folding with 4-, 5- and 6-fold excess and the highest yields (49–50%) for the assemblies with 8- and 10-fold excess of staples.

In conclusion, we devised a novel DNA nanostructure design approach by employing custom scaffolds that allows for successful folding of large (>10,000) nucleotide structures with an order-of-magnitude reduction in staple count compared to similar template-based shapes. We tested several combinations of design parameters, namely staple length, staple and scaffold crossover density, and total number of strands. Future exploration of design space and fine-tuning of low-level parameters may further boost yields and reduce the number of strands required for folding. We hope that our approach will provide useful inspiration in realizing applications of DNA self-assembly that require large-scale production of complex nanostructures.

## Supplementary Material

Supplementary Data

SUPPLEMENTARY DATA
