# Supplementary material for: Folding complex DNA nanostructures from limited sets of reusable sequences
Source: Nucleic Acids Res. 2016 Apr 1;44(11):e102. doi: 10.1093/nar/gkw208 (PMC4914096; doi:10.1093/nar/gkw208)
Supplement: Supplementary Data [file gkw208_Supplementary_Data.zip › 20160324_Supp.pdf]

Supplementary Note:

Cost calculation for DNA nanostructures

Although the prices of conventional synthesized oligonucleotides have decreased significantly over the past years, many DNA origami designs require over 200 distinct oligonucleotides with a length distribution of 20-60 bases. Assuming a design based on a 6000 bp scaffold we will calculate the approximate cost for prototyping of shapes and milligram-scale production of structures with the conventional DNA origami method and the reusable unique sequence method.

DNA origami

Estimating the average staple length to 40 bp, we will need about 150 oligonucleotides. For a 25 nmole synthesis scale we get for each oligo approximately 100 µg. Thus, for 150 oligos we get a total mass of 150 x 100 µg = 15 mg. Commonly, a 10-fold excess of staples to desired scaffold binding sites is used in a molecular self assembly reaction. Since we can not fold more structures than we have scaffolds in a reaction, we end up with about 1.5 mg of oligos in the structure. Since the number of base pairs for staples and scaffold in the final structure is approximately similar, we get 3 mg of structure. Now, assuming a folding and recovery yield of 70% we get 2.1 mg. For the synthesis of the oligonucleotides we assume a cost of $0.29 per base at 25 nmole synthesis scale. Hence,

150 x 40 bp x 0.29 $/bp = 1740 $

for 2.1 mg of folded structure only as the cost for oligonucleotides.

Folding with limited sets of reusable sequences (in this example 20)

For the folding with limited sets of reusable sequences we have higher initial costs for the custom scaffold. Genewiz charges 0.29 $/bp plus and additional 0.06 $/bp for a complicated template. Thus, for a 6000 bp custom scaffold sequence we end up with

6000 bp * 0.35 $/bp = 2100 $

As additional cost for cloning of the custom scaffold (labor, gibson reaction, purification, sequencing) we will assume about 400 $. For the oligonucleotides we have an average length of 65 bp and need 20 different sequences. Here, we can choose a 25 nmole synthesis scale and we get for each oligo approximately 100 ug (as for the origami design). Hence, for 20 oligos we get a total mass of 20 x 100 µg = 2 mg. Again, using a 10-fold excess of staples to desired scaffold binding sites, knowing that the number of base pairs for staples and scaffold in the final structure is approximately similar and with an absolute folding yield of 51% for a design with 20 unique staple sequences, we get

2 mg / 10 x 2 x 0.51 = 0.204 mg

of structure. For the cost of the oligonucleotides we calculate with $0.29 per base at 25 nmole synthesis scale. Hence,

20 x 65 bp x 0.29 $/bp = 377 $

for 0.204 mg of folded structure only as the cost for oligonucleotides.

Comparison - Cost for prototyping of shapes

Assuming that the cost for scaffold production as well as purification, all buffers, labor and equipment is the same for both methods (thus, not included in comparison) we get a yield and cost for the initial order of one shape / design:

Design method	Yield	Cost
DNA origami	2.1 mg	1740 $
Reusable Sequences	0.204 mg	2877 $

Hence, for just prototyping of shapes the DNA origami method is cheaper.

Comparison - Cost for milligram scale production

If we are interested in scaling up, we can choose a 1 µmole synthesis scale for the design with 20 unique staple sequences and we get for each oligo approximately 1630 µg. Hence, for 20 oligos we get a total mass of 20 x 1630 µg = 32.6 mg. Using a 10 fold excess of staples to desired scaffold binding sites, knowing that the number of base pairs for staples and scaffold in the final structure is approximately similar and with an absolute folding yield of 51% for a design with 20 unique staple sequences, we get

$$32.6 \text{ mg} / 10 \times 2 \times 0.51 = 3.33 \text{ mg}$$

of structure. For the cost of the oligonucleotides we calculate with $1.75 per base at 1 µmole synthesis scale. Hence,

$$20 \times 65 \text{ bp} \times 1.75 \text{ \$/bp} = 2275 \text{ \$}$$

for 3.33 mg of folded structure only as the cost for oligonucleotides.
Hence, for approximately 10 mg of structure we can find:

Design method	Yield	Cost	Cost per mg
DNA origami	10.0 mg	8286 $	828.6 $/mg
Reusable Sequences	10.0 mg	9325 $	932.5 $/mg

Thus, the costs are comparable for the mg scale. But we anticipate that the true value of the method will be realized when we move beyond chemical synthesis scale to scalable ssDNA methods that require cloning, where gains will be made at saving the labor of component production, not at the raw synthesis step. Since only 20 unique oligonucleotide sequences are required, the MOSIC (21) method for oligonucleotide production can easily be applied together with our reduced component method and therewith reduce costs significantly further.

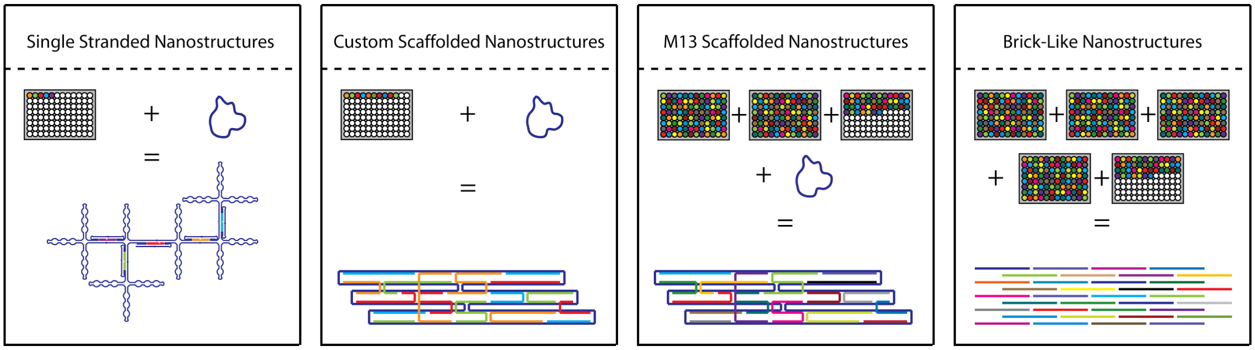

Figure S1: Alternate representation for comparison of number of strands required for (from left to right) single-stranded nanostructures, custom scaffolded nanostructures, M13 scaffolded nanostructures, and brick-like nanostructures. The number of oligonucleotides is represented by the colored dots in 96 well plates.

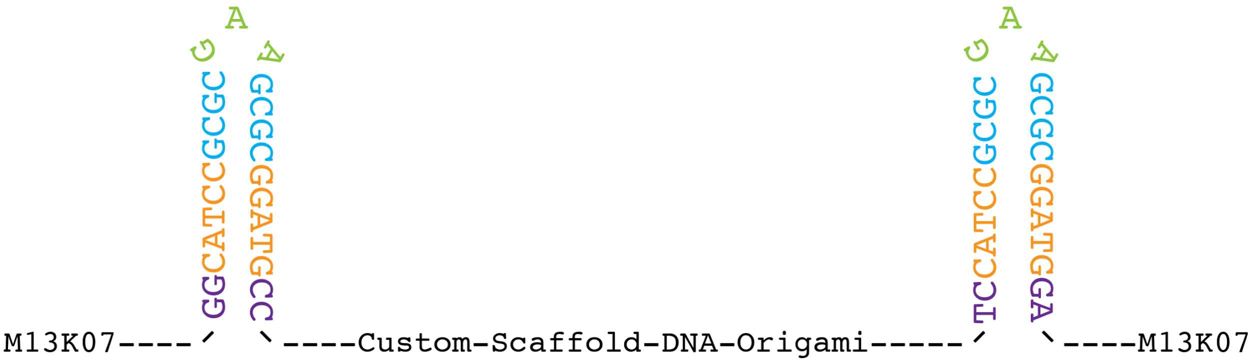

Figure S2: Schematic representation of the vector used to amplify the custom DNA scaffold. The scaffold sequence is cloned into the phagemid M13K07 for production of single-stranded DNA. To facilitate isolation of the custom scaffold during purification, it is flanked by two hairpins encoding recognition sequences for the restriction enzyme BtsCI (orange). The hairpin design is based on the MOSIC method (21).

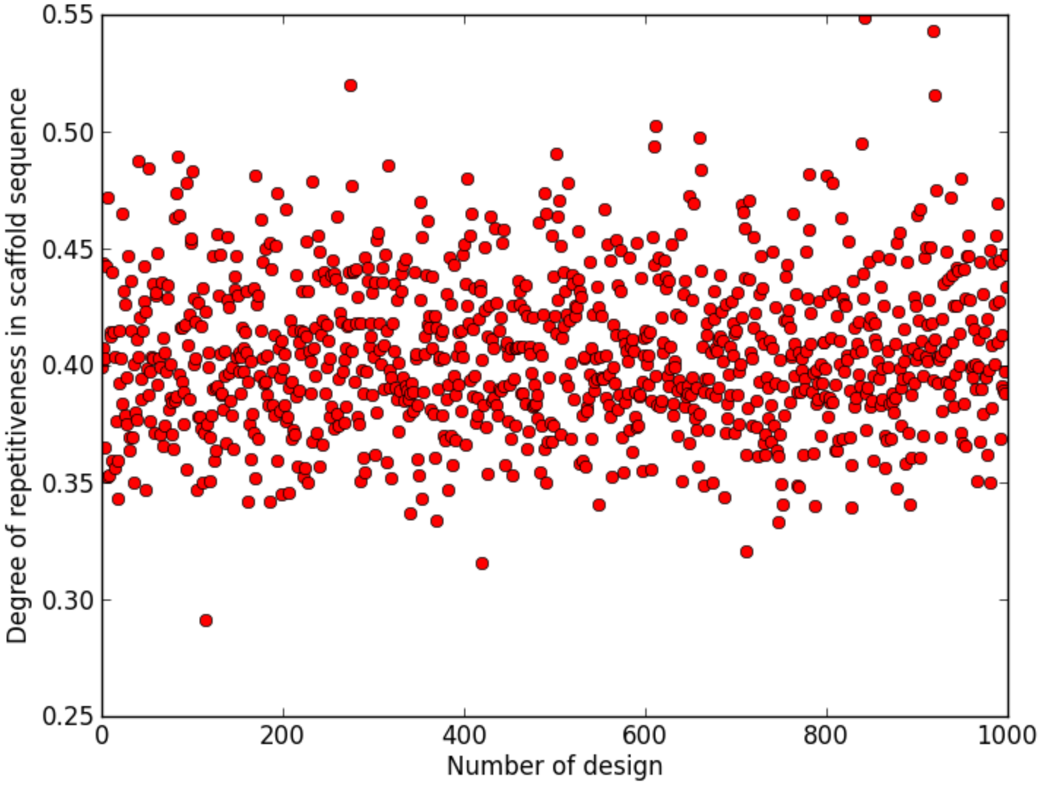

Figure S3: Example output for 24-helix bundle design with 20 unique staple sequences. Degree of repetitiveness for scaffold sequence for each of the 1000 designs with staple length of 38, 56, 63, 70, and 77. Minimum repeat length was set to 12 bp and random staple sequences were used.

Design	Unique staple count (control antenna)	Unique staple count (test antenna)	Average staple length in bases	Number of crossover per 1000 nucleotides	Repetitive staple crossover arrangement	Custom staple length (count of each)
Long staples with Low staple crossover density and Low scaffold crossover density (LLL)	14	7	72	88	No	60 (2), 63 (1), 65 (2), 70 (2), 75 (3), 80 (2), 85 (2)
Short staples with Low staple crossover density and Low scaffold crossover density (SLL)	28	7	36	88	No	25 (6), 30 (4), 35 (5), 38 (1), 40 (6), 45 (3), 50 (3)
Short staples with High staple crossover density and Low scaffold crossover density with repetitive staple crossover arrangement (SHL)	23	6	44	188	Yes	26 (3), 40 (7), 41 (3), 45 (4), 47 (1), 60 (5)
Long staples with Low staple crossover density and High scaffold crossover density (LLH)	10	5	101	208	No	75 (3), 98 (1), 100 (2), 120 (3), 125 (1)
Short staples with Low staple crossover density and High scaffold crossover density (SLH)	24	12	42	208	No	25 (3), 27 (3), 29 (1), 37 (3), 38 (2), 47 (1), 48 (1), 49 (1), 50 (3), 50 (1), 56 (3), 62 (2)
Long staples with High staple crossover density and High scaffold crossover density (LHH)	13	7	78	304	No	63 (1), 65 (4), 66 (2), 79 (2), 86 (1), 101 (2), 107 (1)

Table S1: Design strategies for the six different custom scaffold DNA origami antennae. The first column shows the name of each design.

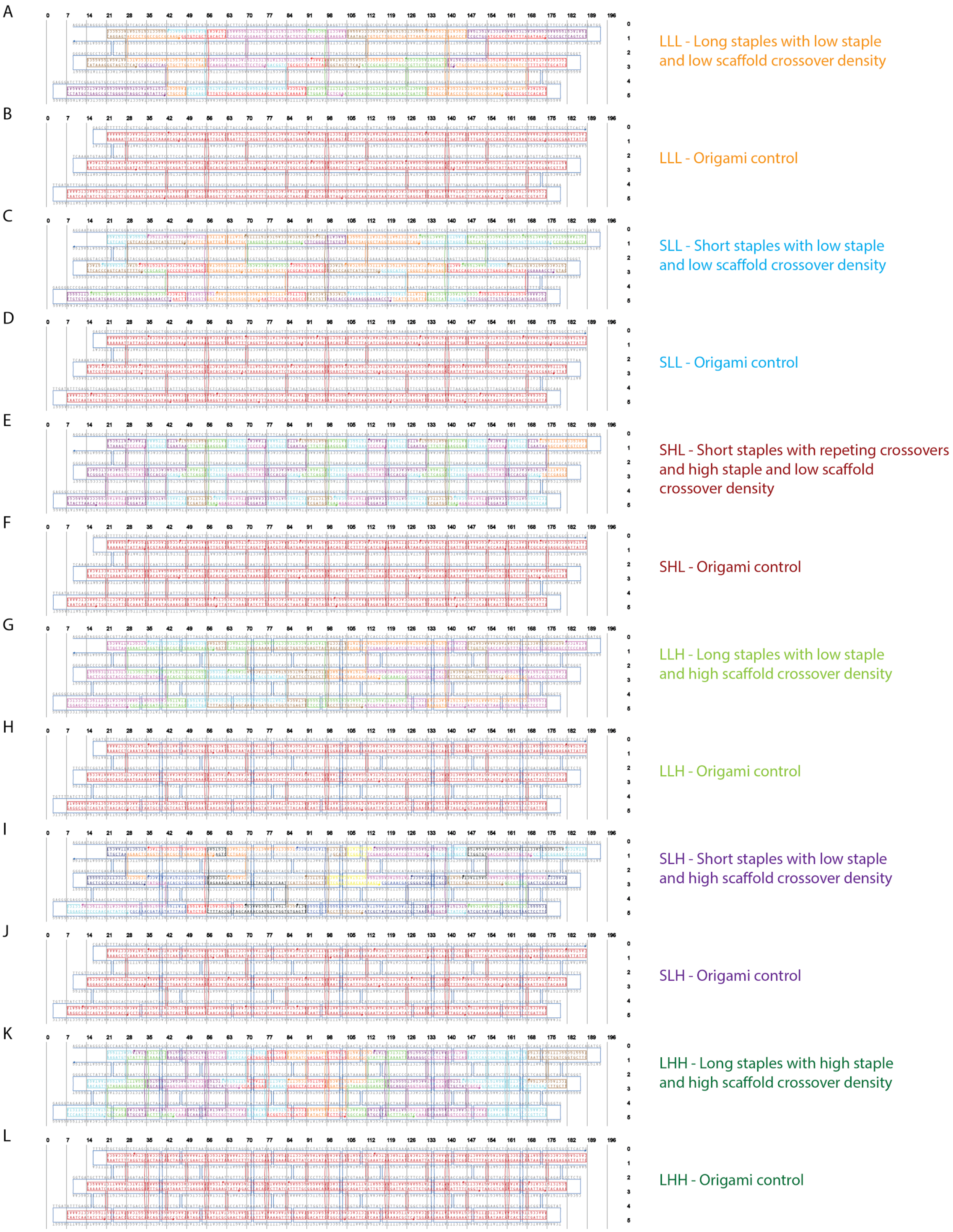

Figure S4: Detailed caDNAno design schematics for the custom scaffold DNA origami and the corresponding control origami antenna as used in the screening device. (A) LLL: Long staples with low staple and low scaffold crossover density and (B) corresponding control origami

antenna. (C) SLL: Short staples with low staple and low scaffold crossover density and (D) corresponding control origami antenna. (E) SHL: Short staples with repetitive staple crossover arrangement and high staple and low scaffold crossover density and (F) corresponding control origami antenna. (G) LLH: Long staples with low staple and high scaffold crossover density and (H) corresponding control origami antenna. (I) SLH: Short staples with low staple and high scaffold crossover density and (J) corresponding control origami antenna. (K) LHH: Long staples with high staple and high scaffold crossover density and (L) corresponding control origami antenna.

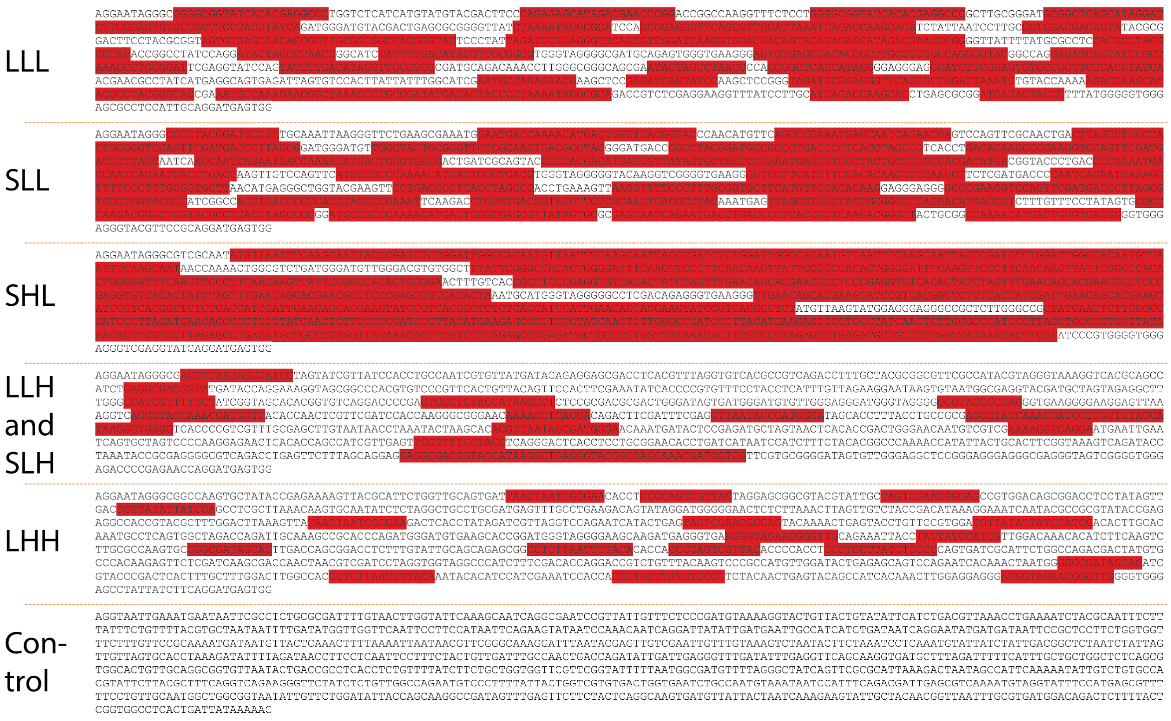

Figure S5: Scaffold sequences for the antennae as used in the screening device. Repetitive motifs of 12-bases or longer are highlighted in red. From top to bottom: (i) LLL: Long staples with low staple and low scaffold crossover density, (ii) SLL: Short staples with low staple and low scaffold crossover density, (iii) SHL: Short staples with repetitive staple crossover arrangement and high staple and low scaffold crossover density, (iv) LLH: Long staples with low staple and high scaffold crossover density, (v) SLH: Short staples with low staple and high scaffold crossover density, (vi) LHH: Long staples with high staple and high scaffold crossover density, and (vii) origami control (sequence is part of M13K07 phage).

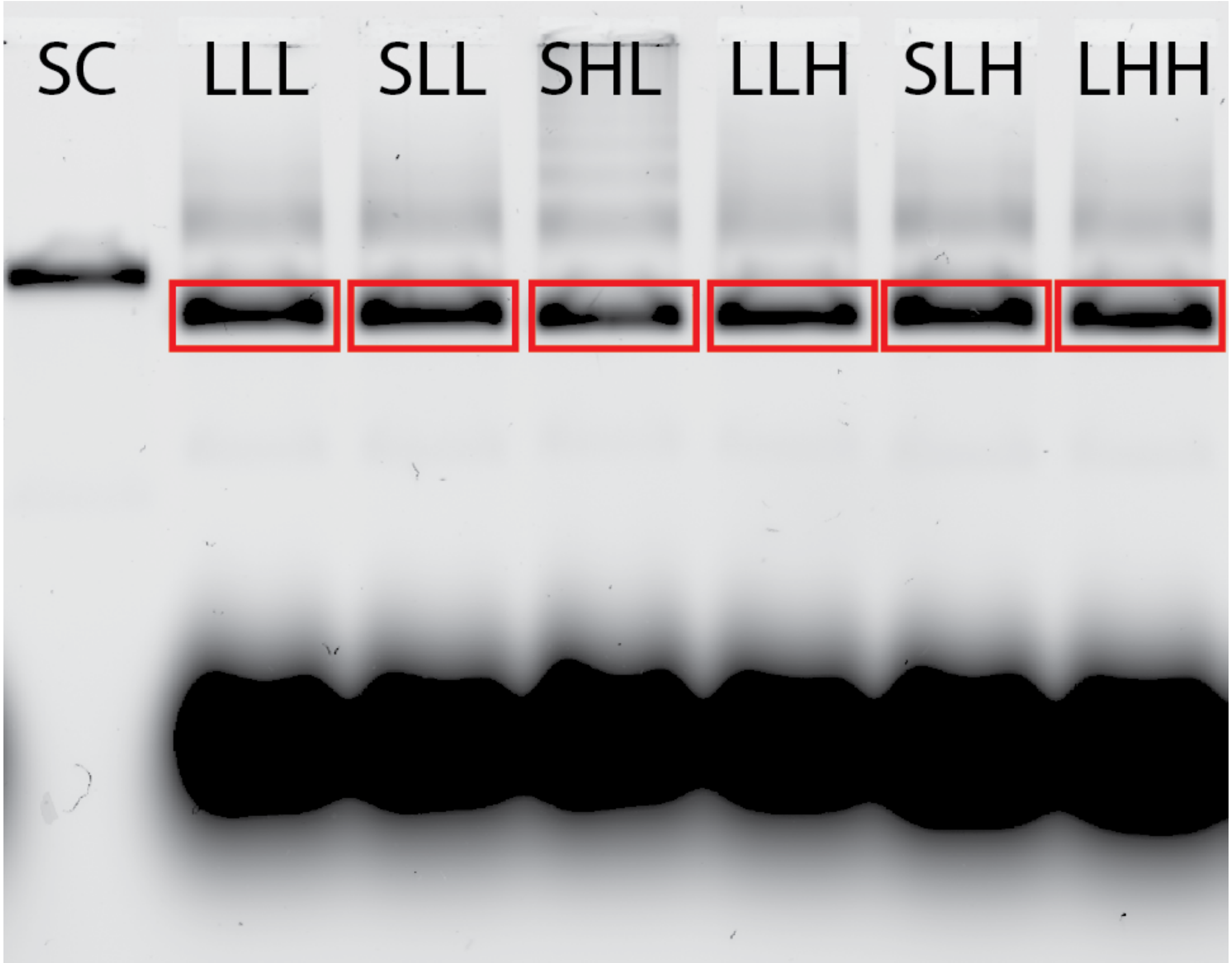

Figure S6: 2% agarose gel comparing migration of unpurified folding products of the six screening device structures with custom scaffold DNA origami and the corresponding control origami antennae. Red boxes indicate the region of each lane that was counted as the fastest-migrating monomeric species that was physically extracted from the gel for purification before TEM imaging. From left to right: (i) LLL scaffold annealed without addition of staples (ii) LLL: Long staples with low staple and low scaffold crossover density, (iii) SLL: Short staples with low staple and low scaffold crossover density, (iv) SHL: Short staples with repetitive staple crossover arrangement and high staple and low scaffold crossover density, (v) LLH: Long staples with low staple and high scaffold crossover density, (vi) SLH: Short staples with low staple and high scaffold crossover density, (vii) LHH: Long staples with high staple and high scaffold crossover density.

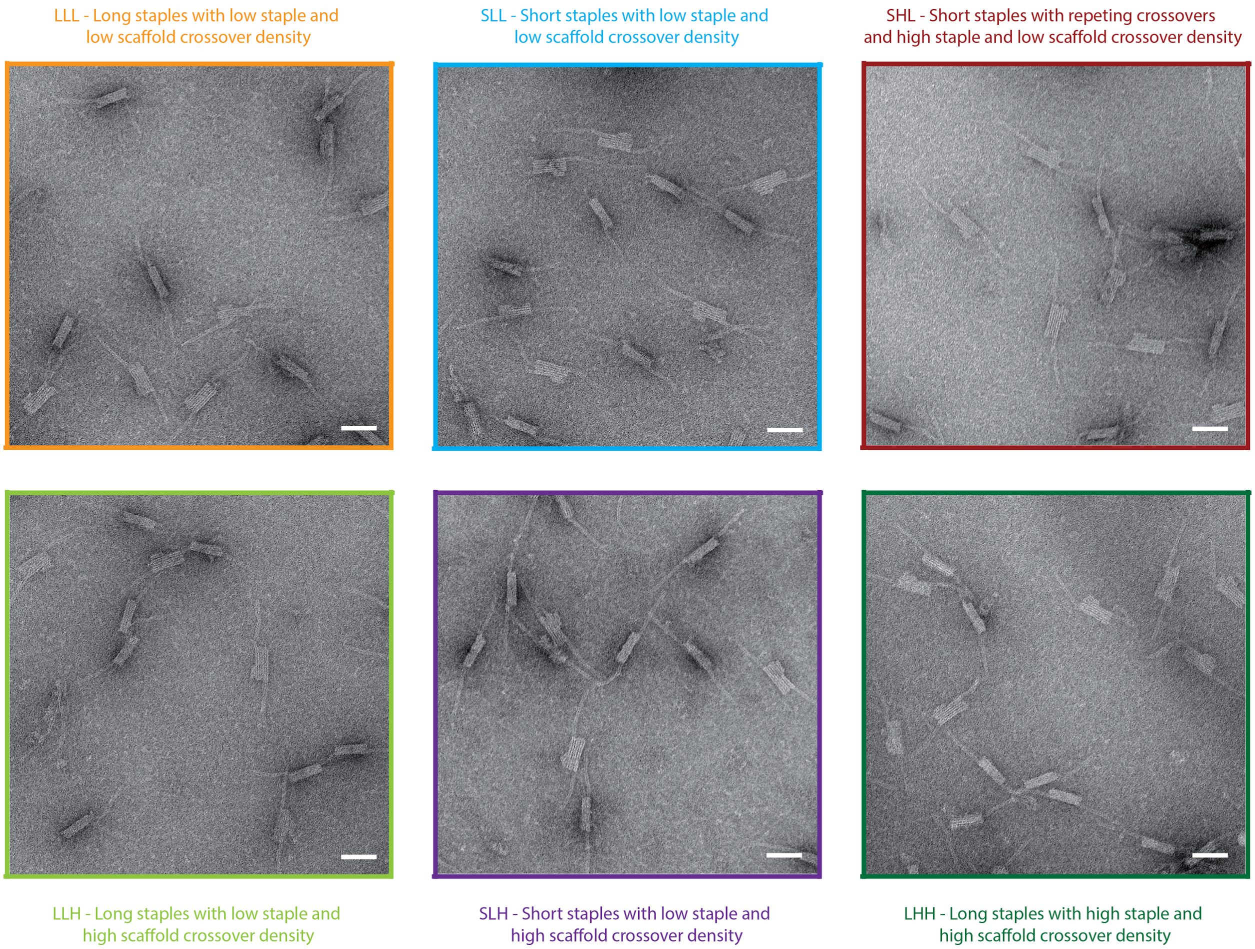

Figure S7: Representative negative-stain TEM micrographs of the testing device with different antennae for the custom scaffold and control origami design. LLL: Long staples with low staple and low scaffold crossover density (orange), SLL: Short staples with low staple and low scaffold crossover density (blue), SHL: Short staples with repetitive staple crossover arrangement and high staple and low scaffold crossover density (red), LLH: Long staples with low staple and high scaffold crossover density (light green), SLH: Short staples with low staple and high scaffold crossover density (purple), and LHH: Long staples with high staple and high scaffold crossover density (dark green). Scale bars: 50 nm.

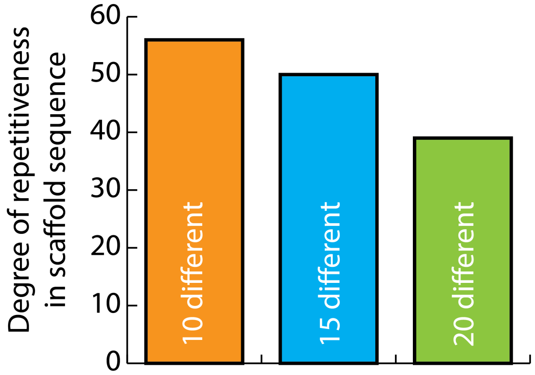

Figure S8: Comparison of degree of repetitiveness in scaffold sequence in a 24-helix bundle folded with 10 (orange), 15 (blue) and 20 (green) different staple sequences.

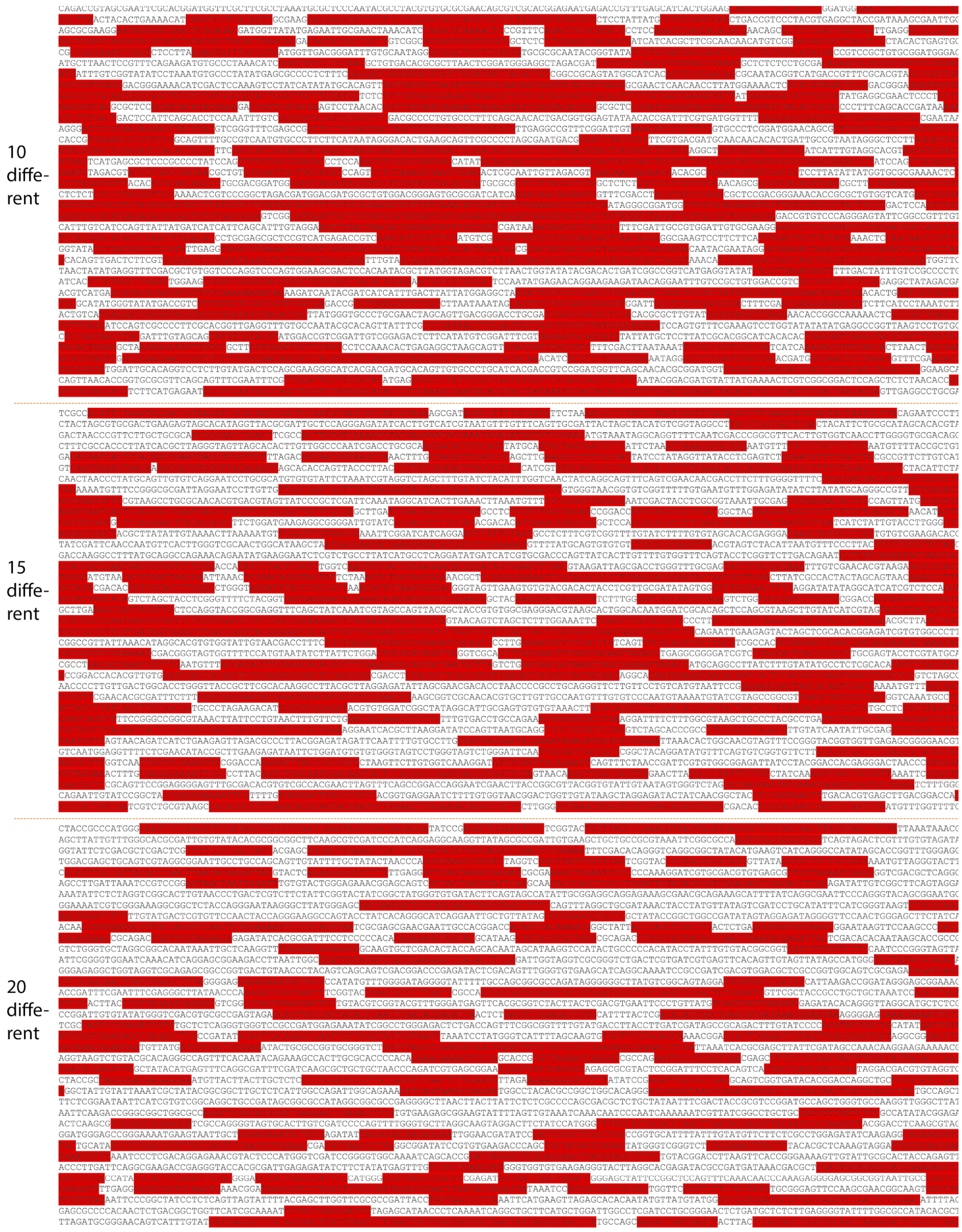

Figure S9: Scaffold sequences for the 24 helix bundles. Repetitive motifs of 12-bases or longer are highlighted in red. From top to bottom: Design with 10, 15 and 20 different staple sequences used for folding of the 24-helix bundle.

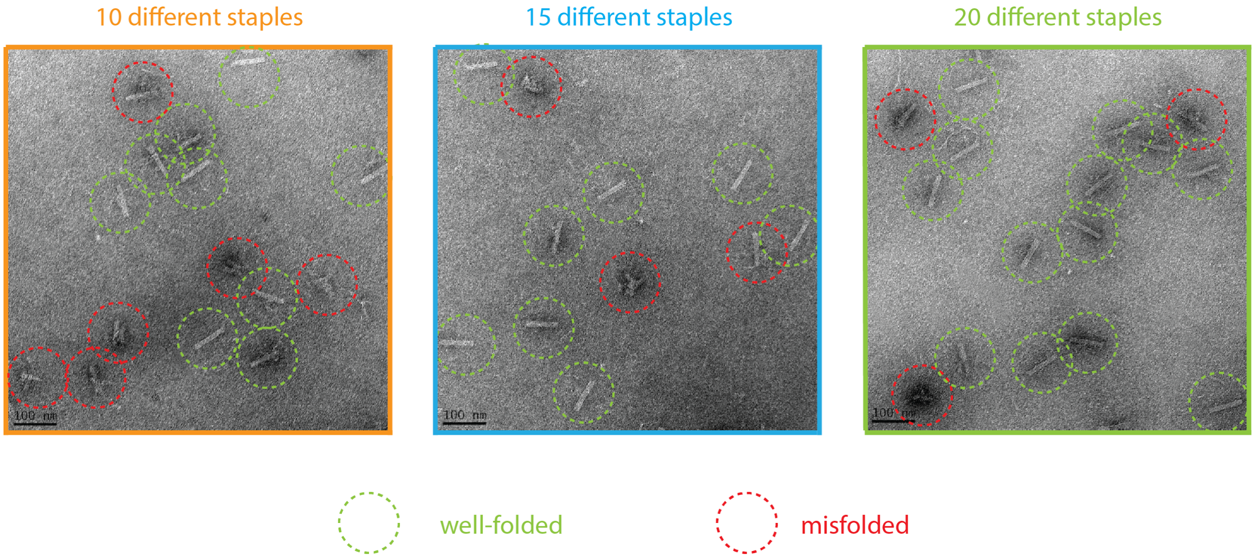

Figure S10: Representative negative stain electron micrographs for the 24 helix bundle with 10 (orange), 15 (blue) and 20 (green) different staple sequences used for folding. Green dashed circles show well-folded structures and red dashed circles highlight misfolded shapes. Scale bars: 100 nm.

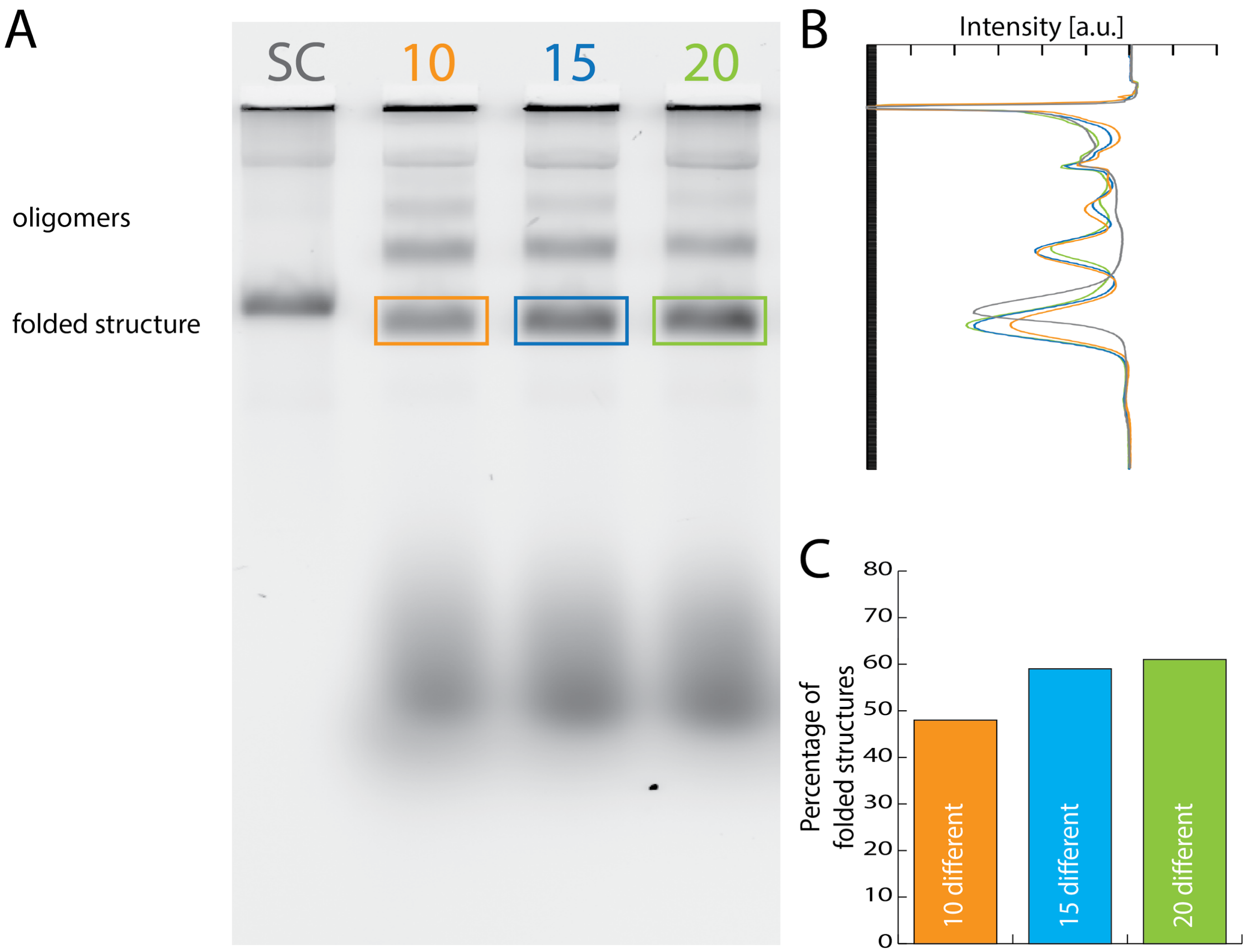

Figure S11: Folding yield of 24 helix bundle with different number of unique staple sequences analysed by gel electrophoresis. (A) 2% agarose gel comparing migration of unpurified folding products of the 24 helix bundle with 10 (orange), 15 (blue) and 20 (green) unique staple sequences. Boxes indicate the region of each lane that was counted as the fastest-migrating monomeric species that was physically extracted from the gel for purification before TEM imaging. “SC” shows migration speed of scaffold annealed without addition of staples (here the scaffold for the 24 helix bundle with 10 unique staple sequences is shown). (B) Intensity profile of each design (24 helix bundle with 10 (orange), 15 (blue) and 20 (green) unique staple sequences) analyzed by using ImageJ. The intensity profile is aligned with the agarose gel from (A). (C) Fraction of scaffold incorporated into fastest-migrating monomeric species, as estimated by SYBR safe-fluorescence intensity.

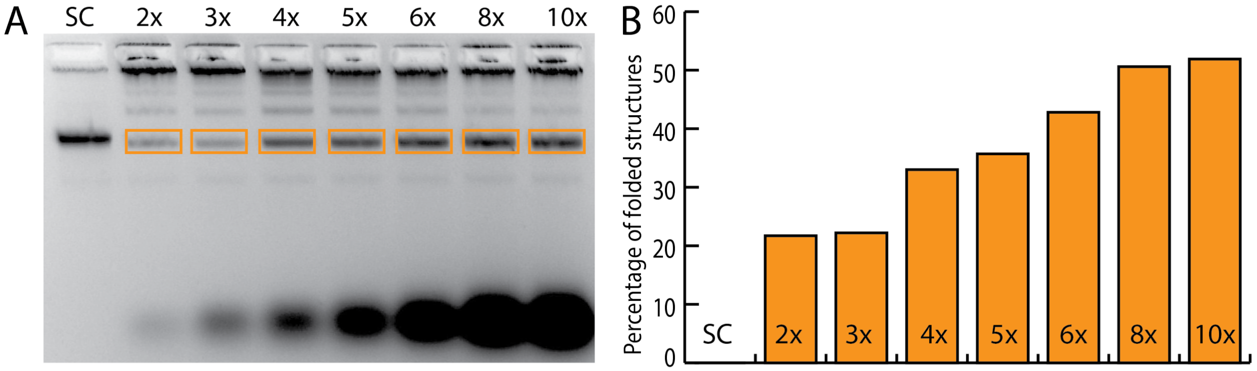

Figure S12: Screen of relative staple concentrations for the 24 helix bundle with 10 unique staple sequences. (A) 2% agarose gel comparing migration of unpurified folding products of the 24-helix bundle with 10 unique staple sequences and various staple concentrations: 2-, 3-, 4-, 5- , 6-, 8-, and 10-to-1 ratio of staples to corresponding scaffold binding sites for custom scaffold DNA origami. Boxes indicate the region of each lane that was counted as the fastest-migrating monomeric species. “SC” shows migration speed of scaffold annealed without addition of staples. (B) Fraction of scaffold incorporated into fastest-migrating monomeric species with relative staple concentrations for 24-helix bundle with 10 unique staple sequences, as estimated by SYBR safe-fluorescence intensity. Here, K91 cells instead of XL1 Blue cells were used to grow phagemid.

caDNAno / json-files:

1. The core of the testing device with the antenna (details of the antenna for each design can be found in Figure S4).
2. Design with 10 different staple sequences
3. Design with 15 different staple sequences
4. Design with 20 different staple sequences

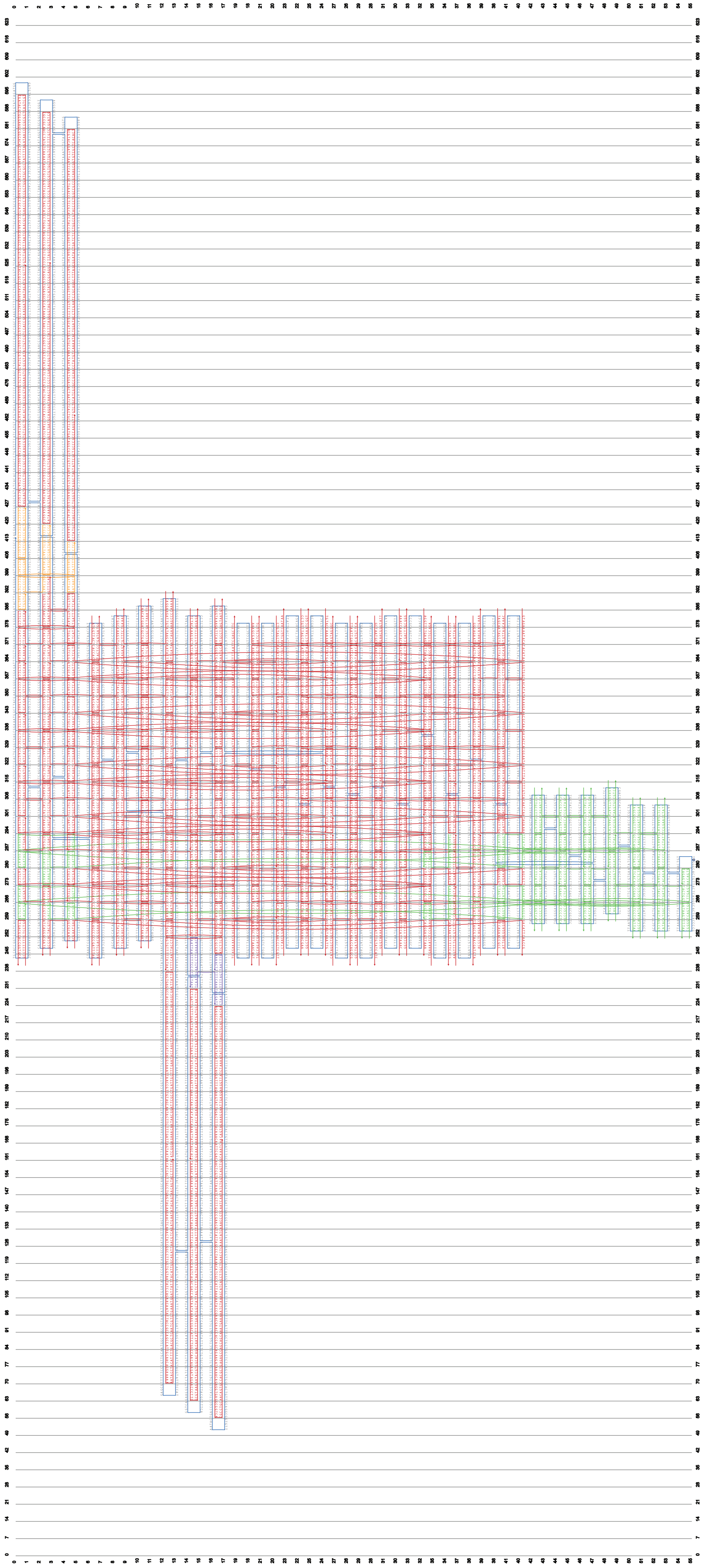

Design with 10 unique staple sequences

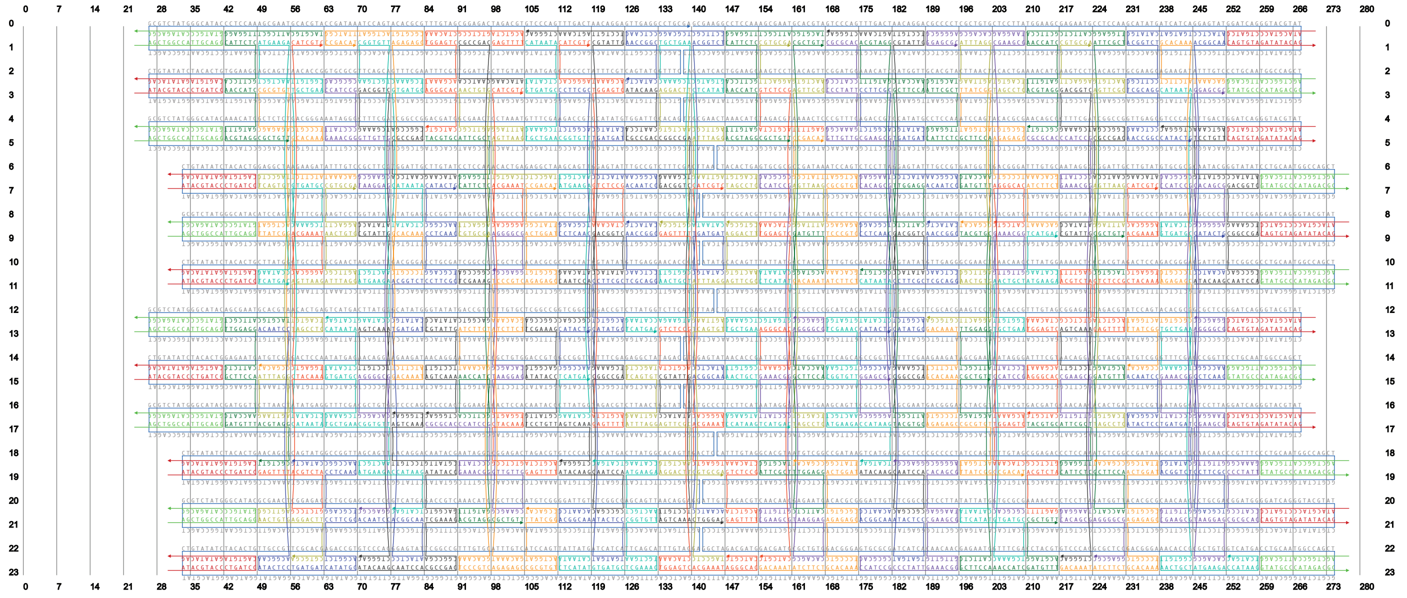

Design with 15 unique staple sequences

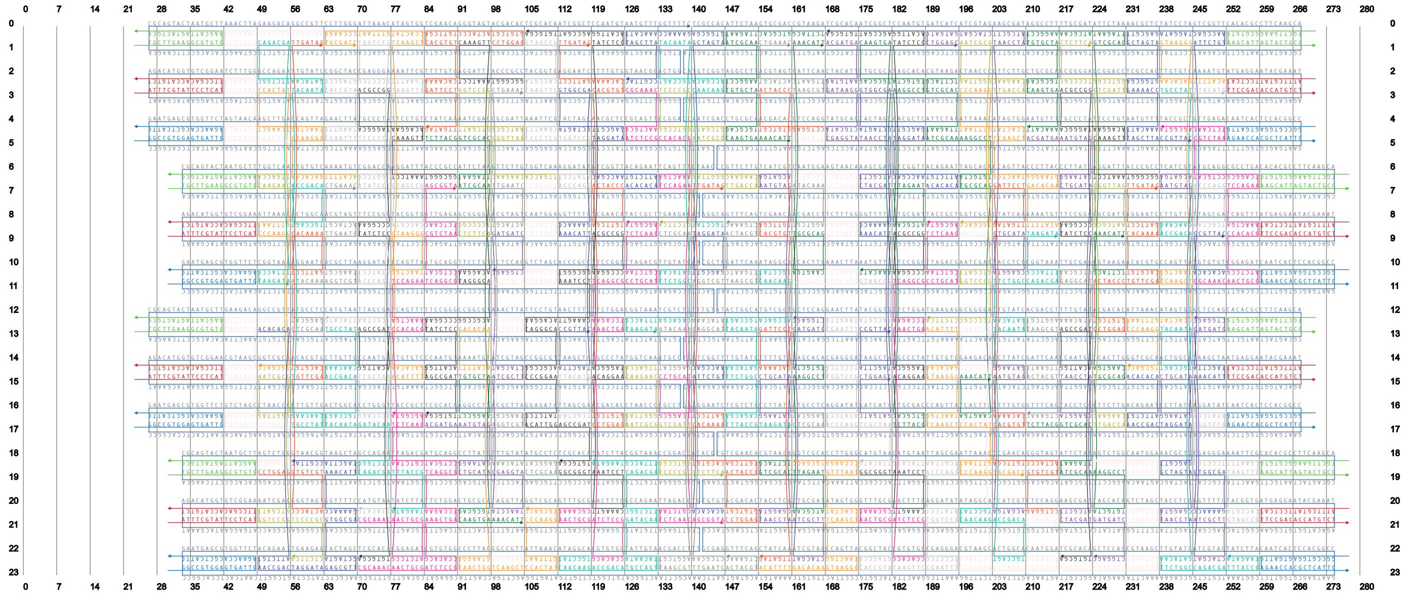

Design with 20 unique staple sequences

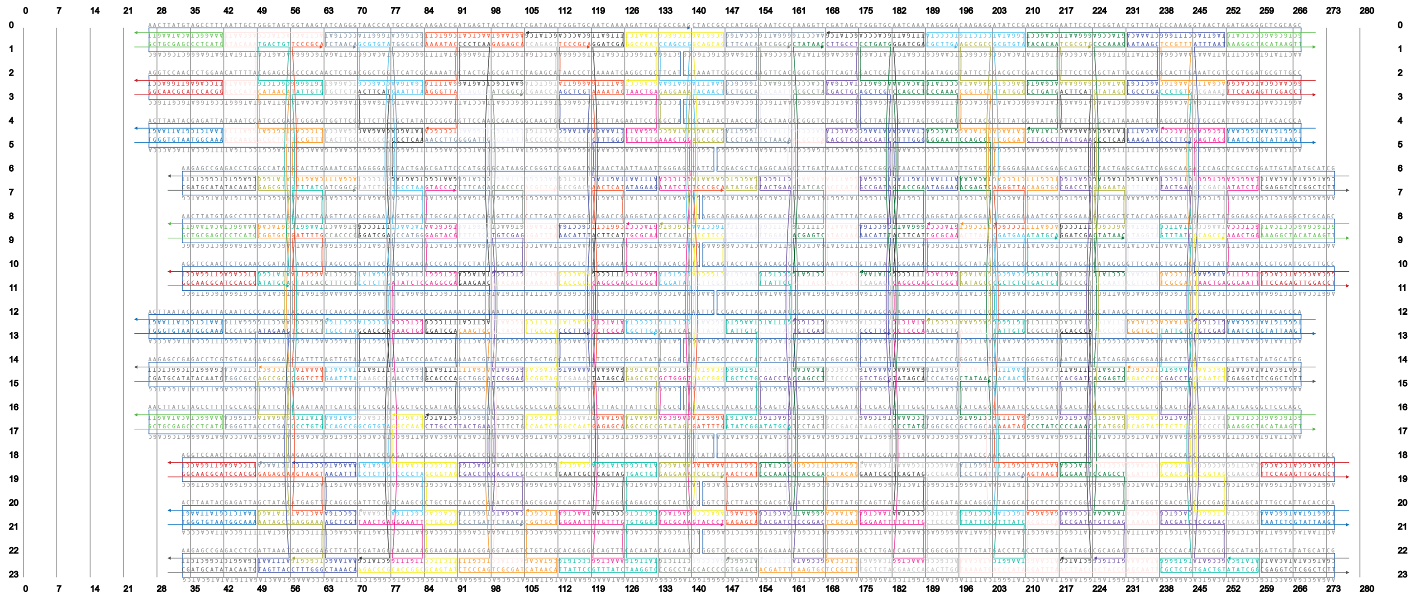

Table S2: Primers for Gibson cloning for 6 kb custom DNA origami scaffold sequences
Upper case bases anneal with DNA block sequence (see below) and lower case bases add DNA for overlap for Gibson reaction to neighboring strand

Name	Sequence
10-different-staples_block01_fwd	agatacttaacagggaagtgagagggccgcggCATCCGCGCGAAGCGCGG
10-different-staples_block01_rev	gtttacaatccgaaaCGGCCTCAACGTATGCCCATAGAC
10-different-staples_block02_fwd	catacgttgaggccgTTTCGGATTGTAAACATCCGCTTC
10-different-staples_block02_rev	acagcgtcgaaacctCATATAGTTAAGAACCATCGTATGCCC
10-different-staples_block03_fwd	gttcttaactatatgAGGTTTCGACGCTGTGGTCCC
10-different-staples_block03_rev	tcaccaactggtcgatccccggggcgagctCATCCGCGCTTCGCGCGG
15-different-staples_block01_fwd	agatacttaacagggaagtgagagggccgcggCATCCGCGCGAAGCGCGG
15-different-staples_block01_rev	tgaacattggttgaaTCGATAAGGTGTCTTCGACACAATAGGGCATAG
15-different-staples_block02_fwd	aagacaccttatcgaTTCAACCAATGTTCACTTGGGTC
15-different-staples_block02_rev	gatacaatctcaacaCGATGAAATGTAGAGGTCGTACATTG
15-different-staples_block03_fwd	ctctacatttcatcgTGTTGAGATTGTATCTATTGTAATAGGCACAATGGAAGTAACACAATCAC
15-different-staples_block03_rev	tcaccaactggtcgatccccggggcgagctCATCCGCGCTTCGCGCGG
20-different-staples_block01_fwd	agatacttaacagggaagtgagagggccgcggCATCCGCGCGAAGCGCGG
20-different-staples_block01_rev	cgggattggaagaacAAAGATGACCGCCGTACACAAATAGGTATGTGG
20-different-staples_block02_fwd	acggcggtcatctttGTTCTTCCAATCCCGGGTAG
20-different-staples_block02_rev	gccaatcttgccttaCTGAACTTTCTGCCAATCTGGC
20-different-staples_block03_fwd	tggcagaaagttcagTAAGGCAAGATTGGCCTACACGCC
20-different-staples_block03_rev	tcaccaactggtcgatccccggggcgagctCATCCGCGCTTCGCGCGG

Table S3: Staple sequences for core of testing device

Name	Sequence
core_1	CAGAACGATTTAATTCGCAAGAGCTTAGATTAAGAGCTTCTG
core_2	GACCATTGCGCGAGTCCAGACACAACGC
core_3	ATCTCCGCGGATTCAGGTGGCCCTTATTGAAGCCCAATAATAAGAGCAA
core_4	TTTCAACATGGATGCTGATGGCCGCGATTAAATTCTTT
core_5	TTATTTTCAATCAATAATCGGATAGATAAATTCTGCTGAAAAAATTAAG
core_6	TTTTTAAGAAAAGTGCATGATCATATTACCTGAATATTTTGC
core_7	TTTTGTACCAAAAACCAGTAGGGCTTT
core_8	CGAGGCGAGTTGCTCTTACCAACCGATA
core_9	TTGAAAGTGCTCCAAAGCGCGAAACAAAACTACGATTTTCATTTAGTAA
core_10	TATCCTGATGCGAGCAGCGCTCGGAAATAGGAGGTCACCACCCTCATTT
core_11	TTAGCAAGGTGGCAAGTAGATTTAGTTTTAAAGTA
core_12	TTTTAGCAATAGCTATCTTACCACGCAGTGCCAGTTCGAGCGTCTTAAAT
core_13	TGCGGGATTTAGAAAGTCAAATGATAAATTCATGCTGAGAGT
core_14	CAAAATTAATTACAGGCCGATAAGGCGTATTCAGT
core_15	AATACAAGGCAAAAATCGGTGTTAAATTACCGGTAATGAACTTTTTTAA
core_16	TTTGCCTAATTTATGTTAGCAAACTTT
core_17	TCATACAAGTAGTATGATTCCCAATTCTTTCATTC
core_18	TTTTCTACAAAGGCTATCAGGTCATTGCCCGGAGAG
core_19	ACCGTCTATCAGGGCGATGTTT
core_20	CATTTCCATAAGCTACGCAAAAGAACTGAAGCAGACACAAGAATTGAGT
core_21	TTTTTGAAAATCTCCAAAATAATAATTTTTTCACGTTT
core_22	TAGCGTCCAGAAGCAAAGCGGGGCTTAGGAATAAAAATCAGCACCAATA
core_23	CGTAAAGGAAGGGAGCGCTTAATGCGCCCTCGTTGGGGGGGGTCTTGAC
core_24	AAAGCCACACAAGATCGGAACACCCAAAGCGAAAAAGCGATCCGTCCCG
core_25	TGCTCGATTATATGCCAGAGT
core_26	ATTATGAAAATGCAAGGGTGAGGTAGCTATTTTTGAGAGGTTT
core_27	TCATATGTACCCCGAAATTGTATATTTTCCCTGTA
core_28	TAGCCGAACAAAGTACGGAATAATAAGACTTAAACCGATAGT
core_29	TTTATGGCAAAGGTATGATGATGCTTT

core_30	TTTATTGAGGGAGGGCTCAGAACCGCCTTT
core_31	ATATCAAAATGAAAGGAATCAAGAAGGCGCATTTTGTATCATAAAATTA
core_32	ACCCGCCAGAAAGCGAAAGGAGTTGATGAATAAAACATCGCCGAACGAG
core_33	TTTGCTAATGCAAGAACGGGTATTTTT
core_34	TTTCATAGGCTGGCTGACCGTGTACAGACCAGGCGTTT
core_35	TTGAGATATACCAGTAGCCGGCGAACTG
core_36	TTTGGGTAAAATACGTAATGCCGTACAACCGAAATCTCTACGTTTTAATT
core_37	TTTGCCTGATAAATTGTGTGGAGATTTGTATCATCTTT
core_38	TTCTCACCCCCGTTCGTAAGCCGCAGACGGCGGCTTTGTTGACAGAAAC
core_39	ATCGTAAAACTAGCTGTTAAAAAAAATTGAAGCCT
core_40	TGAGAGAAACCTCCATTGCATTGGTTTAAAAATCTCATGTAACACTAAT
core_41	AAACAGCTAAGACTAACATATAAAAGAATTTGCCACCAGGATGGCTTTT
core_42	TGCGATTGCTCATTTTAGGAATACCACAGCAGATA
core_43	ATTTCATTTGAATTTTTGTTACACCGGACTAGAAA
core_44	TTTCAGGAAGATTGTATAAGCATGTTTCTGAAACTTT
core_45	TTGCGGATGGCTTAACTCCAACAATAAAATACTTTCAGTATA
core_46	TTTTTAATTGAGCCGACTTGCGGGTTT
core_47	TTTCTCTGATGTCAGGGAGTTAAATTT
core_48	TAATTGTTTTATCAAGCGGAGTTGCTAACAAACTACAACGCCTCAGGGA
core_49	CATATTCCGCGATATTGTATGAATATTTATGTCAACTGGAGCAAACAAG
core_50	CTAAACTATTTATGCCTCTTCTTCCAGGTATGAGCACCCAGCGTATTCT
core_51	CAGGTCACGAAAGACTTCAAACGGAGACCCCTCATAAACGTTGGAAGCC
core_52	TTTGCCTTTTTTCTCCTGCCTGACGCACCGGTGCATTT
core_53	TTTACCCTCAGAACCGCCACCC
core_54	AACGACCAACAAGTAGTTTATAATAATATACCAGAATGTACCGTAGAGA
core_55	GAACCTCAATCGCCCGCTCAA
core_56	TACATTGCGTTGTGTATAACGCCAGAATGAAGATGTAAATCGTCGCTAT
core_57	ATCGCAGACGCTAAACAAAATAAAAACACACCCTGAACAAAGTAAGCCC
core_58	TTTTCAGAAAACGAGAATGAAATGCTTTAAACAGTTTT
core_59	CTTCCTGGTGATTTCTCACTTAGGTTGTAATTGCAGTTTCAT

core_60	TCGCGTAAGGAGCCAACAACTATGAATTTTCCACAGACAACCTCAGAGC
core_61	GAAAGGCTATCGCGGAAGCAAGAGCTTATTTAAATATGCAAC
core_62	TTTATGGTTACTTCCTGATGCAGAGCTTCAAAATCACCAA
core_63	CAATCCAACCCAAAGACACCACGGAATACTGGAAAGAACTGCTTTCTCC
core_64	CTGTCTGAAAGAATAGTTTTATCATTGATTTAACAAGTACATAAATCAA
core_65	GACCTGCCATGAACCGGGTGCGTCGACTCGTGGACGTTGAGTGTTGTTC
core_66	TTTCCTGTTTAGCTAACATGTTCATTT
core_67	ACCAGTAACAACTTAGCAACGGGTAGCAAATCATACAAATCA
core_68	TTTGTAGAAAATACATACATAAAGTCGTCAGTCGGATTGATAA
core_69	ATGAAACAAACATCACAGGAAAATACCGCTGACGA
core_70	TTTTAGCTCGAGAATCCTTATCATTCCAGAACGCGATAAACATATTTTC
core_71	GCGAACGTCAATTCACAAAAGGGCAGAG
core_72	TTTAAGTTTTGTCGTCTTTTTAGCGTAACGATCTATTT
core_73	ACAATGATTCGAGGTCGATTC
core_74	TCACCCTGTCGCTGCTCCAAATTTCGTC
core_75	TAATTTAGTAAAGTAGTCCTGAACAAGATAGAAACCATCGTAATAGCAG
core_76	AGGAAACCGTCACCCACCGAGCAGTTTCGCTTGCTCAACAAC
core_77	TTTTGAAGAAACGCTTTGTCACAATCAATGGCTGGCTGTTTGTTCCTGC
core_78	TTTAAAGCGAACAGCTAAATCGGTTTT
core_79	ACAGGTAAGAAAAACGCGACCAGGACAG
core_80	ATCATCGATTTTTTCTACCATTCAACCGAGAATCG
core_81	AGCATAACAGACCGTTTTAATTCGAGCTTCTTT
core_82	TTTGGGAGAATTGAAACAATGAAATTT
core_83	TTTTACCAGACGACGATAATATCATAACCCTCGTTTTT
core_84	CGTCATACTTTACCCTGACTACTATATGGATAAATTAAAGGGAGCGGGA
core_85	GCTGCTCTAAATAAGTTGGGTCAAAATCATAGGTCTGGAAAC
core_86	CCTAAAGCCGGCGAGGCGCGTACTATGGTTGCTTTT
core_87	GCTACAGACGTGGCGAGAAAGCACTAAATAAAAATATATTCGCAGCAGC
core_88	TTCAAGTGCGTTAAGGAAGAGGTAATTCAGCAAAT
core_89	TTTAGGTTTTGAAGCCTTTCCAGATTT

core_90	CACCACTGTACTCCGCGTTGCCAATGATCGATGCGGGTATAAAAGCCAA
core_91	TTGGACGACTCATGGCATCTTCCAGGAAGCAAAAG
core_92	GCAATCAGGGGTGTTATTAGATTCATTAATAAATCCATCTTCCCGACAA
core_93	CTTACATCAGGTGAGCCGGTTTTAGAGTGAACAGAAGGATCA
core_94	TTTGCCCACTACGTGGGCGCAATCTTT
core_95	CGAGCCAAATATAATATCCCATCAAACACACTACTGCCCAATTCTTTAA
core_96	TGGTTGTAACACTGATGAATAATTGATG
core_97	TTACCGCAATTTACGAGCATGAAAATAAAGTACCGTACTAATGGCAAGG
core_98	CCTGTTGGAGCGTAGCGAGTCCAGTTTGCCACTATTAAAGAAGGCAGTG
core_99	AAATATTGCGGGCGCGCGTAACCACCACGCTAAACAGCCCGACATTACC
core_100	GCTACAGGAGGCGAAAGAATACTCATCTCGCAGACCAAACTGTTAAGTC
core_101	TTTACGAATGAAGACATTCAACCGTTT
core_102	ATGGGCTAACGGGACCGGGAATCAAAAAACGGGACCGTTCCGTGGCAAA
core_103	TGTAGCATTCTGTAGAGGACTGAAAGACACCAACTAAGAACC
core_104	CTTGCCAATTTTATTTTATCCCCTTTACGTAATTGAGCGCTAGATAACC
core_105	GCCTCAGATTTGGGAGATACATTTCGCAAATGGTCAATAATTT
core_106	TTTGCAAAAGAAGTGCGGAATTACCTTAGAATAAGGGATATT
core_107	TTTGGCTTGAGATGGTAATAAAACGAATTT
core_108	TTTCTAACGGAACAACATTATTGCAACAC
core_109	ATATCATAGGGGGGGAATCAGATTTTAGAAGAAAAGAGTGAATAACCTT
core_110	ATATTTAGACGACACCTGTTTATCAACACTGTCTTCAAGCAAATAACAT
core_111	TTCTAGCTCACCATGGAAGCCGGATTAGGTCATTTCGGTGTCTGGAAGT
core_112	GAGGGGGTAATAGTGACTGGACAATAAAAAACAAAACGTAGCTAAATCA
core_113	TTTGGCCGCTTTTGCTTTCCATTAAACTTT
core_114	CACGCTGCTAGGGCGCTGGCACCTGATTAAACAGTTGCGCCGCAGATAT
core_115	CTCGGATTGAATTTAACGATTCGTCAAACACTGAGTTTCGTCGGAACCC
core_116	TAATCATGTTGGGAGAAAGATTCATCAGGAATTAC
core_117	TTTAGCTTGACGGGGAAAGGGAGCCCCCGATTTAGTTT
core_118	CGCTGAGTGATGCAATCAGGTAATATTCAGAGGCTGAGGCATAGTAAGA
core_119	CCTTTTGATAAGAGAGAGTACCAAAGAATTATTTCATGCGTT

core_120	AACTGAAGGGAAGCGCACTCA
core_121	AATTTATTATATAATTATAGTCAATACTTTTGCCACATAACGCCAAAAG
core_122	AGGGAACAACGAGGTTGACCCCCAGCGAAACGAAAAGGCTTTTGGGGTT
core_123	AAACCAAAATAGCGATTGAATTCAACTTGAAACACAGAGTAA
core_124	ATTACGCTGACTTGTATGGTA
core_125	GAAATGCGCTCGCCGCAGTCGGATCACGGAACTTTTGCTGAGGCTGAGT
core_126	AAAGACTAGGCACCAACCTAATTATACCTGTTACTTCAGGACTGTGAAT
core_127	TTTCTGTAGCTCAACATGTATTGCTGAATCTGGTGTTT
core_128	AGTGAGCAGCGTAATAGAAAAGAATTATGACTTGATAGCAAGCCCAATA
core_129	ATTTCAATTACCTGAGCAAAACTTGAGAGACCTAAAGTAGTAAATTGTTT
core_130	TTTTTGACGAGCACGTCTCAAAATTTT
core_131	GCTTGCCACCGTGTTAAATGCAAGAGTCAATAGTGTATATGT
core_132	CCCCCTCACCATAAATCAAAAAATCCAAGGTTTGACGGTACGTGCTTTC
core_133	GTTACAGCAAGCATTTTATCCGCGATCCAACGTCTCAAGATT
core_134	TTTTAGGTAAAGATTCAAAATGCCTGAGTAATGTGTTT
core_135	AATATTTGTTGATAATCAGAAAAGCCCCAAAAATTT
core_136	AACAGCACGACCATATGAGATGGTCAGACTATCGAATGTCGGATACAAA
core_137	CTTTAATCAAAAAGATTAAGACAATATGCAAGGATATTCGCGCGACAAT
core_138	TCAGAGGAGAGAGAGCCGTTTAAGAACGCAACATGTTCTTAC
core_139	AGCATCGCACGCATGTATCGGCCTTTTA
core_140	TTATCCGTAGATACAGCTTCATCCTATG
core_141	TAGCGATACAAAGAACGCGAGATCTTCTAGTGTTTTTATAAT
core_142	TTTAAACCAAGTACCGCATTAGACTTT
core_143	AGAATATAGACGGAGGCTGTGTAGCGGTGGCCGAATCCCTTACTGTTTA
core_144	CTCATAGCCAGACGGAGGAAGGGGATCGATGAACGTTCATCA
core_145	CTCCCTTTTCTAATCAGAATTTTGATGCTCGATGAGTACCCC
core_146	GGTTAATATCCCCGGGGCGAGTCTCACTTTATTTTTGCCTCC
core_146	CGCCCTATTCCTCCGCGGCCCCTCGTCGACCCACTCATCCTG
core_147	CCCTCACGAGGGGAAATTAATGATAACCTCCCTGTTAAGTATCTGGTCG

Table S4: Staple sequences for asymmetric part of testing device

Name	Sequence
asym_1	TTTTAGCGGGGTTTTAGGCGGATAAGTGCCGTCGTTT
asym_2	TTTGTTTGCCTTTAGCGTCAAAGCGATCAGTTCGAAACCATAGCAAG
asym_3	TTTCATTACCATTCGATAGCATTT
asym_4	TTGATGATACTGGTAATAAGTTTTAATTT
asym_5	TTGGTTGTATCACATTAGCGTGGCATTTCTTGAGTAACAGTGTAGAGTG
asym_6	GCCATTTGCCAGCAGCCGGAATCAAGAGGAAAGTATTAAGAGGCCCCCT
asym_7	AACCGCCCCACCACCCACCGTGTATCGCCGCCAGCATTGATTT
asym_8	CCAGAGCCTTTTCACGGAATAAGATAGGTCCAACGTCAAAGGTCAAGTT
asym_9	AAAGGAAATATTGAAAAGACACCCTCAGGGAACCG
asym_10	TTTAGCCAGAATGGAGCCGCCACCTTT
asym_11	TTTGCACCGTAACAGAATCAATTT
asym_12	TTTGCCCCCTTATATTCTGCACATGA
asym_13	TTTCAGGAGGTTGAGACGAATGGATCCTCATTAATTT
asym_14	AAATCACTAAGTATAGCCCGGTGGTACC
asym_15	TTTAGAGGGTTGATACAGTAGCACTTT
asym_16	TACAGATGCTGAATGTCTCTGTTCACAAGCAGGTCAGACGATAATAGGT
asym_17	TTTGAGCCGCCAAAAGGGCTAACGGTTCGCTCAAACCATC
asym_18	GCTCAGCCTTGATAAGACTCCACCAGAG
asym_19	GATGACGGAGGAAGTAATCAAAGACTGT
asym_20	ACCCTGTGGTTTACTGATTTTACAGCGATTTTGGG
asym_21	ACTTAGAGGGAATCTATTCATAGAAAGGTTTAATTAACCGAT
asym_22	AGCGCGTTTTCATCTTGCCATCACCACC
asym_23	TTAGTACACCAGAGCCTCAGAAAGCGCATTACCGTTCCAGTAAGCGTTTT
asym_24	TGAGAATTAAAGGTTTCATATCACCAATACCGGAAGCCTATTTCGGAACCTATTTTT
asym_25	TTTATTCTGAAACATAAGGATTAGGATTTT
asym_26	TTTAGAACCACCCGCCACCAAGGTAATTGCGAAAAAAAGGAGGCTTG
asym_27	TTTCGGGGTCAGTGCTCGGTCATATTT
asym_28	TTTCATACATGGCTTCCTCCCTCATTT

Table S5: Staple sequences for Long staples with Low staple and Low scaffold crossover density (LLL)

Name	Sequence
LLL_01	ATCCGCAGGCTTTAGCGGATACTGATCTCGGAGCTTCGTTCTTTGGCATTCCTGGATAGGCCGGT
LLL_02	CCGGGTTCGTCTATGCTCTCTGGGAAGTCCGCGTATACTGTCGTCCACCGCAAGGATAAACCTTCCTCGA
LLL_03	CTGCCCGTAGGCGTGTGCTTGGTCTGATTTAATCCAACGCTGAACCCTCCGCATCTACCCCTGGCCGCCGCAAGC
LLL_04	CCGCGCTCAGTCGTTGATACGTGGGAAGCGGCACACTCCGAAGATCCTATGCTGAGCCGCTGGGGTTAGGCTAGTATTCG
LLL_05	GGTGTCGCTCACACTACGTACATTTTGTCTGCATCGCGCCGCAACCTATGTCAAAATAGATGCCATGCGCCTATTTTAGATAACC
LLL_06	GGGCCTCGTGTGATACCGCCCCAGGAGTAATAGATGGAGGCGCATAAAGGGTAGTCTCAT
LLL_07	GACGGTCAATAATAAGTGGACACTAATCTCACTGCCTCATGACCCAAGGACATGATGAGACCA

Table S6: Staple sequences for Short staples with Low staple and Low scaffold crossover density (SLL)

Name	Sequence
SLL_01	CCGCAGTAGCCACGCTAAGGGTCATCGAACTGGAC
SLL_02	TGGCCGATGGTCAGGTGCTTCAGAACCCTTAATTTGCA
SLL_03	TCATTCTGATTGCTCATTTCGGGCTAGGTGAGGGGTCAGG
SLL_04	CTTCGGGCTTGTGTCGAACATGAAGCACCGCAAAGGGAAAACCTT
SLL_05	AACTTCGTACCAGCCCGTCTTGAGCGCACTATAACGCTCATGTCGTGGCC
SLL_06	GTACCGTCACCCAGTCATGTTTTGG
SLL_07	GCGGCATCCGTAGGCGTCAGTTGCGAGAAC

Table S7: Staple sequences for short staples with high staple and low scaffold crossover density with repetitive staple array (SHL)

Name	Sequence
SHL_01	TGTGGCCCTTGAAATTCGTGCTGTTCAATCTAAGGGCAGC
SHL_02	ATCTCAGGCACTAGATCTTGTTGAAGGGAAAATCCAGTGAG
SHL_03	AGAGCCGTGATTGATAGACGAAGTGTTTATAGGAGGCGTCCCCAG
SHL_04	TGTCGAGGCTGCATTTAGCCACACGTCCCAAGACGCCAGTTTTGGTT
SHL_05	CGCTCTTCATCGATGGAGATCGGGTA

SHL_06	ATTGCGAATAAAGTGTGACACCTCGACCACGGGATCGGCCCAAGAGCGGATACTTAACAT

Table S8: Staple sequences for long staples with low staple and high scaffold crossover density (LLH)

Name	Sequence
LLH_01	ATCGCTATTAACGTGTGCTAACTCCTTCTCGGGGTGACTCGCCGTACCCTCAGCCTTATGGTACAGGTGG ATCGAACGACCATCGTTTGCTACCCTCGCGGAGCCTCCCAACACTATCCC
LLH_02	TTCCGCATAGAAAGATGGATTATCTACGTATCAATCTGATTGGCGAACGCCGCGAGTGCAGTAATATGGT TATCTGAACCTAAACGCCTCGCGCTACC
LLH_03	AGTCGCGTGACCTTTACCGATAGCAAAACGATGGCTGGTGTGAGTTACTAGCATCATTCCTGACCTTTTG TTCCC
LLH_04	CGCAAACGATAGTATTTAGGTTATTGGACACGTGGGCCGTGGGGCTCCTCTGTATCATACCGTCGCCTCA GAACTCAGGTCTGACGCCTGAGGTCAGGTG
LLH_05	GCCCTTGCCCTGACACCCTCGAAATCGAAGTCTGTACACTTATAAAGGTGGGTAAATGAGGTAGGAAACA CGGGGTGATAGTATCTCGGATTACTGTAACACGACATTCGAGTGAACGACAAGCT

Table S9: Staple sequences for short staples with low staple and high scaffold crossover density (SLH)

Name	Sequence
SLH_01	AGTCGCGTGACCTTTACCGATAGCAAAACGATGGCTGGTGTGAGTTACTA
SLH_02	ATCGCTATTAACGTGTGCTAACTCCTTCTCGGGGTGACTCGCCGTACCCTCAGCCT
SLH_03	CCTCGCGGAGCCTCCCAACACTATCCC
SLH_04	TATGGTACAGGTGGATCGAACGACCATCGTTTGCTAC
SLH_05	CGCAAACGATAGTATTTAGGTTATTGGACACGTGGGCCGTGGGGCTCCTCTGTATCATACCG
SLH_06	TCGCCTCAGAACTCAGGTCTGACGCCTGAGGTCAGGTG
SLH_07	CGCGAGTGCAGTAATATGGTTATCTGAACCTAAACGCCTCGCGCTACC
SLH_08	GATAGTATCTCGGATTACTGTAACACGACATTCGAGTGAACGACAAGCT
SLH_09	GTCTGTACACTTATAAAGGTGGGTAAATGAGGTAGGAAACACGGGGT
SLH_10	TTCCGCATAGAAAGATGGATTATCTACGTATCAATCTGATTGGCGAACGC
SLH_11	GCCCTTGCCCTGACACCCTCGAAATCGAA
SLH_12	GCATCATTCCTGACCTTTTGTTCCC

Table S10: Staple sequences for long staples with high staple and high scaffold crossover density (LHH)

Name	Sequence
LHH_01	GCACTTGGCAAGATGTCGATGGATAATAGGAACAGTTGTACTCCCGTTCTATCCAAGTTTGTGAT
LHH_02	GGCCAGATGCAACTGTAGAGGGGAGATATGGATTTGTATTTGTAAAATTCCTCTCGACTTTAAGTC
LHH_03	TCTGGCTGCTATCAACTATAGGTGAGGCAAAGAAATACGTACGGTGGAAGAGGCAACGACTGGGGTGGAGCAGGGGATTAGTTAATCACATGCGTAGTATA
LHH_04	CCATGCGAGTTTAATACATGGCGGGAGAGAAGCCTACACGGTCCTGCATCCTCCCCGTGGGCCTACCACCTATAGTTGGGAGAACTCTTGTGGGTAGGCATCTTCCT
LHH_05	CAAATCTTCGCAGGTGGGGTACGATACCTAGTCGCTCTGCTCTCAGGACTAGCGGTCCGCTGTCCACTAACAGTCGCCC
LHH_06	CGCGTAGTCGCACAGCCCTTTATGTGACGTGGATCCGAAGTTTAAGAGAGTTATACTGAACTCATCGCAGGCTATGATCTCGCTTT
LHH_07	CTTGCTTTCCTAGCCTGGTCGGTGCTTCTGGGTGCGGCTTTGCATCTGCTAATTATCACTGAG

Table S11: Staple sequences for origami control for long staples with low staple and low scaffold crossover density (LLL)

Name	Sequence
LLL-origami_01	CAGCCATTGCAACAGGAAAAAAAAAAATATATCTACCTACATAATCGTCTGAAATGGATT
LLL-origami_02	TACTTCTTTGATTAGTAATAACATCACCCTTTTATGATTATCAGATGAAGAACCCTTCTG
LLL-origami_03	GACATTCCACCGCCTGCAACAGTGCCACGCTGAGAGCCAGCAATTGAGGAACAATATTACCGC
LLL-origami_04	ACCTGAAAGCGTAAGACACCAGCAGAAGATAAAACAGAGGTGAGGCGGTCACTAATAGAGTAGAA
LLL-origami_05	GCCGTCAATAGATAATACATTTGAGGATTAACGAACATACGTGGCACAGAAGAAGGAGCGGAATT
LLL-origami_06	TTCATCAATATAATCCTGATTGCAGGTTTAACGTCAGATGAATATACAGTAACAGTATTGCCTGATTAGA
LLL-origami_07	GAACTCAAACTATCGGCCTTGCTGGTAAAGATTTTTTTGGATTATACTTCACACGACCAGTAATAAAAGG
LLL-origami_08	ATCATCATATTCCCATCGGGAGAAACAATAACGGATTCGCCTGATTGCTAACCGTATTAGACTTTACAAACAATT
LLL-origami_09	TATTTTTGAATGGCTATTAGTCTTTCTGATAGCCCTAAAACATCGCCATTAAAAATACCGTAGAAGTTGTAGCAA
LLL-origami_10	GAAAGGAGCAAATGGCAGATTCACCAGTCTGAATTATGGAAGGAATTGAACCAACCTATTAGCACGTAAAACAGA
LLL-origami_11	GAGGCGAATTATTCCAGTGAGGCCACCGAGTAAAAGAGTCTGTCCATCACGCAAATTTTGAATACAAAGAAACCACCCAA
LLL-origami_12	ATTTACATTGAAAAATCTAAAGCATCACCTTGCTGAACCTCAAATCAATCAATATCTGGTCAGTTGGCAAATCAACAGTA
LLL-origami_13	CGACAACTCGTATTACCGCGAAAATGCGAACGTTATAGTTTGAGTAACATTATCATTTTGCGGAACCAAGTTACAAAATCGCGCA

LLL-origami_14	AATAAAGAAATTGCGTTATCCAGAAGGTTATCTAAAATATCTTTAGGTGCACTAACAAGTATTAATGGCCAACAGAGATTGGCAA

Table S12: Staple sequences for origami control for short staples with low staple and low scaffold crossover density (SLL)

Name	Sequence
SLL-origami_01	TTATAGTTTGAGTAACATTATCATT
SLL-origami_02	AGATGAAGAACCCTTCTGACCTGAA
SLL-origami_03	AACCTATTAGCACGTAAAACAGAAA
SLL-origami_04	GAAGAACTCAAACTATCGGCCTTGC
SLL-origami_05	AGGTGAGGCGGTCACTAATAGAGTA
SLL-origami_06	TACATAATCGTCTGAAATGGATTAT
SLL-origami_07	AGCGTAAGACACCAGCAGAAGATAAAACAG
SLL-origami_08	TGCAACAGGAAAAAAAAAAATATATCTACC
SLL-origami_09	TTGCGGAACCAAGTTACAAAATCGCGCAGA
SLL-origami_10	GGATTCGCCTGATTGCTAACCGTATTAGAC
SLL-origami_11	GGCGAATTATTCCAGTGAGGCCACCGAGTAAAAGA
SLL-origami_12	GTCTGTCCATCACGCAAATTTTGAATACAAAGAAA
SLL-origami_13	ATATAATCCTGATTGCAGGTTTAACGTCAGATGAA
SLL-origami_14	TCGCCATTAAAAATACCGTAGAAGTTGTAGCAATA
SLL-origami_15	TTACATTGAAAAATCTAAAGCATCACCTTGCTGAA
SLL-origami_16	AGAGCCAGCAATTGAGGAACAATATTACCGCCAGCCAT
SLL-origami_17	CTTCTTTGATTAGTAATAACATCACCCTTTTATGATTATC
SLL-origami_18	GCGGAATTATCATCATATTCCCATCGGGAGAAACAATAAC
SLL-origami_19	TGGTAAAGATTTTTTTGGATTATACTTCACACGACCAGTA
SLL-origami_20	CATTTGAGGATTAACGAACATACGTGGCACAGAAGAAGGA
SLL-origami_21	TAAAGAAATTGCGTTATCCAGAAGGTTATCTAAAATATCT
SLL-origami_22	ATAAAAGGGACATTCCACCGCCTGCAACAGTGCCACGCTG
SLL-origami_23	TATACAGTAACAGTATTGCCTGATTAGAGCCGTCAATAGATAATA
SLL-origami_24	CCTCAAATCAATCAATATCTGGTCAGTTGGCAAATCAACAGTAGA
SLL-origami_25	TTTACAAACAATTCGACAACTCGTATTACCGCGAAAATGCGAACG
SLL-origami_26	CCACCCAATATTTTTGAATGGCTATTAGTCTTTCTGATAGCCCTAAAACA

SLL-origami_27	AAGGAGCAAATGGCAGATTCACCAGTCTGAATTATGGAAGGAATTGAACC
SLL-origami_28	TTAGGTGCACTAACAAGTATTAATGGCCAACAGAGATTGGCAATTCATCA

Table S13: Staple sequences for origami control for short staples with high staple and low scaffold crossover density with repetitive staple array (SHL)

Name	Sequence
SHL-origami_01	AGGCGGTCACTAATAGAGTAGAAGAA
SHL-origami_02	AAAATACCGTAGAAGTTGTAGCAATA
SHL-origami_03	GAGCCAGCAATTGAGGAACAATATTA
SHL-origami_04	AGAAACATTTGATTTACATTTGAGGATTAACGAACATACG
SHL-origami_05	ACATCACCCTTTTATGATTATCAGATGAAGAACCCAGGTG
SHL-origami_06	AACGTCAAACTATCAGGTGCACTAACAAGTATTAATGGCC
SHL-origami_07	TACAAAAGAGTCTGGACAACTCGTATTACCGCGAAAATGC
SHL-origami_08	ACGTAAACAGCCATAACAGTAGAAAGGAGCAAATGGCAGA
SHL-origami_09	CTGGTAAAGATTTTTTTGGATTATACTTCACACGAGCTGA
SHL-origami_10	CGCAAATTTTGAATACAAAGAAACCACCCAATATTCATTA
SHL-origami_11	TTCACCAGTCTGAATTTAAAGAAATTGCGTTATCCAGAAGG
SHL-origami_12	AACAGAGATTGGCAATATACAGTAACAGTATTGCCTGATTA
SHL-origami_13	TGGCACAGAAGAAGGAATTCGCCTGATTGCTAACCGTATTA
SHL-origami_14	GACTTTACAAACATCGCTTTGAATGGCTATTTTGCGGAACCAAGT
SHL-origami_15	GAGCCGTCAAAAAACAGTTCTGACCTGAAAGATATTCCCATCGGG
SHL-origami_16	TTATCTAAAAGTGCCACCCAGTAATAAAAGGCTGATTGCAGGTTT
SHL-origami_17	TGGTCAGTTGTCACCTTTGAAATGGATTATTACCAACCTATTAGC
SHL-origami_18	GAACGTTATAGTTTGAGAGGCGAATTATTCCAGTGAGGCCACCGAGT
SHL-origami_19	GGAAAAAAAAAAATATATCTACCTACATAATCGTCGCTGAACCTCAAATCAATCAATATC
SHL-origami_20	CCGCACAGAAAATGGAAGGAATTGATACATTGAAAAATCTAAAGCAGCAAATCTGCAACA
SHL-origami_21	CTCAGATGAATTCATCAATATAATCGACATTCCACCGCCTGCAACATATCTTTGGCCTTG
SHL-origami_22	AAAATCGCGCAGTAACATTATCATTAGTCTTTCTGATAGCCCTAAAACAATTCTCCATCA
SHL-origami_23	CTTCATAACGGGCGGAATTATCATCCGTAAGACACCAGCAGAAGATTAGATAAAGTAATA

Table S14: Staple sequences for origami control for long staples with low staple and high scaffold crossover density (LLH)

Name	Sequence
LLH-origami_01	CCACCGAGGGATTCGTGATTAGTAATAACATCACAGTTTTAGTACCAAGAACTCACTTTTTCAGGTTTAACGTCA
LLH-origami_02	CAATGACCTGACAACAGTAGGAGATAGAAGTATTAGACTTTACAAAACTCGTATGCCCGAACGTTATAGTTTGAG
LLH-origami_03	TAACCACGACCAGTAATAAAACATTTGAGCCAGTCAATTATCATTTTGCGGAACACAGGATTGAAATGAAGGAGC
LLH-origami_04	TAGATTATATCTTTAGGTGCACTAAAGGGATCCTTTTAAACATTCTGGCCAACAAAAGGAATTGAGGAAGGGCAAATAAGCGTAAGCAATATCCGAAC
LLH-origami_05	CCTGTAATGCGTGGTCAGTTGTTGGCTTTGAATATCTAAAAGAGGCACAGACAATATAAAGCATCACCTCAAATATCAAACCCTCAATAATACGTGCCGT
LLH-origami_06	ATCAATATATGATTATCAGATGATGTGACGCTCAATCGTCGACACAATTCTATTTACATTGGCAGATTCAGATTTAGAACCCTTCTAGATAATCAACTAA
LLH-origami_07	GGAATTATCATCATATTAGAACTATCCCCATCCTGATTTCGGCAACTAGTTTGGATTATACTTCTGCTCATAAACGAATTATGGAAGGAATTGAACCAACCTGAGTTTGCCATTTAGCAC
LLH-origami_08	TGATAGCCCTAAAAAAAAACCTTGCTGAAATACCGAAGGAGCCAGCAGCAAATGAAAAATCTTTATTAGTCTTCAACAGTGCCACGCTGACAGAAGATAGAGGCGGTCAGTATTAACACCG
LLH-origami_09	GTAAAACAGAAAACTTCTTCCTGATTGCAAACCGTAATTAGTTACAAAAGCGTCTGTCCATCACGTATACAGTAACACATCGGGAGAAACAATAACTAAAAGAGAATTATTCCAGTGAGG
LLH-origami_10	GATGAACATGTAGCAATTAAAGAAATTGCGTAGATGCTGGTAAAAAATTAATCAATATTACCGCCAGCCATTGCAACAGGACCACAAGAAAAGGAAATACCTTAAATAATTTACATTTGCAATTC

Table S15: Staple sequences for origami control for short staples with low staple and high scaffold crossover density (SLH)

Name	Sequence
SLH-origami_01	ACTCACTTTTTCAGGTTTAACGTCA
SLH-origami_02	GAACACAGGATTGAAATGAAGGAGC
SLH-origami_03	GTATGCCCGAACGTTATAGTTTGAG
SLH-origami_04	AACCAACCTGAGTTTGCCATTTAGCAC
SLH-origami_05	AACTAAAAGAGAATTATTCCAGTGAGG
SLH-origami_06	AGATAGAGGCGGTCAGTATTAACACCG
SLH-origami_07	GATGAACATGTAGCAATTAAAGAAATTGC
SLH-origami_08	TATACTTCTGCTCATAAACGAATTATGGAAGGAATTG
SLH-origami_09	AATCTTTATTAGTCTTCAACAGTGCCACGCTGACAGA
SLH-origami_10	TCCATCACGTATACAGTAACACATCGGGAGAAACAAT

SLH-origami_11	CAGATTCAGATTTAGAACCCTTCTAGATAATCAACTAA
SLH-origami_12	ATCACCTCAAATATCAAACCCTCAATAATACGTGCCGT
SLH-origami_13	GTAGATGCTGGTAAAAAATTAATCAATATTACCGCCAGCCATTGCAA
SLH-origami_14	AACAAAAGGAATTGAGGAAGGGCAAATAAGCGTAAGCAATATCCGAAC
SLH-origami_15	CAGGACCACAAGAAAAGGAAATACCTTAAATAATTTACATTTGCAATTC
SLH-origami_16	TAGATTATATCTTTAGGTGCACTAAAGGGATCCTTTTAAACATTCTGGCC
SLH-origami_17	TAACCACGACCAGTAATAAAACATTTGAGCCAGTCAATTATCATTTTGCG
SLH-origami_18	CAATGACCTGACAACAGTAGGAGATAGAAGTATTAGACTTTACAAAACTC
SLH-origami_19	CCACCGAGGGATTCGTGATTAGTAATAACATCACAGTTTTAGTACCAAGA
SLH-origami_20	GTAAAACAGAAAACTTCTTCCTGATTGCAAACCGTAATTAGTTACAAAAGCGTCTG
SLH-origami_21	GGAATTATCATCATATTAGAACTATCCCCATCCTGATTTCGGCAACTAGTTTGGAT
SLH-origami_22	TGATAGCCCTAAAAAAAACCTTGCTGAAATACCGAAGGAGCCAGCAGCAAATGAAA
SLH-origami_23	CCTGTAATGCGTGGTCAGTTGTTGGCTTTGAATATCTAAAAGAGGCACAGACAATATAAAGC
SLH-origami_24	ATCAATATATGATTATCAGATGATGTGACGCTCAATCGTCGACACAATTCTATTTACATTGG

Table S16: Staple sequences for origami control for long staples with high staple and high scaffold crossover density (LHH)

Name	Sequence
LHH-origami_01	TACAAAAGCGTCTGAAAGAGAATTATTCCAGTGAGGCCACCGAGGGATTATAACTATCCATCA
LHH-origami_02	CGTATACATGAACATGTAGCAATACTTCAATAACAGAGTAGAAGAACTCCCTTTTAGTACCTTGC
LHH-origami_03	GAGCCAGCAAAATCTTAAAATTCTAAAGCAGAAAATCTTGGCACCTCAAATCAATCAATATCTGG
LHH-origami_04	TAACGTCAGAGTAACACATCGGGAGAAACACGTGCCTTCACTCCTGATTGCAAACCGTAATTAGT
LHH-origami_05	AAGAATAACTTATCTAACAAATATTTTTTAATTTTTATTAGCCCTAAAAAATCCTTACAAACAAT
LHH-origami_06	CAATTCACCTCACCCAGATTGATTGTTTACGGATTGAAATCAACCATATTTTGACGAAAACGCTCA
LHH-origami_07	TCACACCTATTAAGAACAGTAGAAAGGAGTTATCTTAGGTGCACTAACATAGCCACACAGTGAGCC
LHH-origami_08	GTCACAACAGAATAAATTTGAGGATAAGAACCCGAACCTCGTATTACATCGCCAATGCGCGAACTGAGAA TGGCTATTA
LHH-origami_09	TGGACCAGCCAACTTCTGAACAGAAATATCTTATTAGCACGATCGGAAACTTAAAAGAAATTTTAGTTTTG AGCAGGTT
LHH-origami_10	TCGTAGATGTTATCCTATTTACATTCCACCAGAAAGGAGTCACACGACCAGGACATTCAGATAGAACCCT TCGCATTATGCGGAGT
LHH-origami_11	TCGACTTAGAAGTATTAGTCTTAATAATAGATTATTAAAAATTAGATACTAAACACCAGCAGAGGAAGATT GAAGGGTGAGGCGGTCAGGCCTGCAGCTGA

LHH-origami_12	TGGTACATTTTCGTAGAATATTACCGAAGAAATAAACATACCTACATCAAAATTATGGAAGGAATTGAGGATTATTTGCAACAGATAATTCAATGACTCAA
LHH-origami_13	GACTCACAGCGTGGTTTGCCCGAACTGAAATGACCGTAAAAGTTTGAAAGGTAATAAGATTTTGCGGAACAAAGAAGGACATCATATTCCTGAATAACAGTGGCCGG

Table S17: Staple sequences for connection between origami control antenna and core for scaffolds with low and high scaffold crossover density

Name	Sequence
Low-scaffold-conector_1	TAATTAGTTTCAAATATTTGC
Low-scaffold-conector_2	AATCCTTGAAAACATAATTAATTTTCCCTTTTAAA
Low-scaffold-conector_3	AATCCTATTTTAGTTAATTTCAAAACTT
High-scaffold-conector_1	TCGCGAGTTTCAAATAATACC
High-scaffold-conector_2	AATCCTTGAAAACATAATTAATTTTCCCTTCAGAG
High-scaffold-conector_3	TTTGATATTTTAGTTAATTTCAAAACTT

Table S18: Staple sequences for folding with 10 different staple sequences

Name	Sequence
10-different-staples_01	TACGTGCATTCGCTTTGGAGGCATTCTCGCTTCCAAACCATCGATGTTTACGTAGGCGCTGTT
10-different-staples_02	TTTATACGTACCCTGATCCCAGTGTAGATATACAGTTT
10-different-staples_03	TTTAGCTGGCCATTGCAGGGTATGCCCATAGACGCTTT
10-different-staples_04	GAGTTTTACGTCTAGTCTCCGCTACAAATGGAGTCACGAAATAGGGCACCATCGTC
10-different-staples_05	CATAATACTCATATGTGATGCTCGAAACTGCTGAACGGTGTTAACTGCTATGAAGACCATAAGTCATGAC
10-different-staples_06	CGCGCACCCATCCGCCCTATTGAAACGGGTTGTTGCGAAGCGTAAGGAGCACAGCGAGGGGCGGGAGCGC
10-different-staples_07	GACAAATATCTTCTGCACAAATCCCGTCAGAGAGCCGCGTGTACTGGATTTATCGGCCGACAT
10-different-staples_08	TAGCCTCTCAGTGTAGTTAAGATTTAGGAGTTCGCAACTGTGAGGACTTCGTGCGA
10-different-staples_09	ATACAAGCAATCCACGCCGACCGGCCGATCGAAAGGACGGTCATATACCCGTATTGTCCTGTTAGTCAAACTGGGAC

10-different-staples_10	ACAATCCACGGCAAATACTCCTGATGATCATATGCACGGTCTCCTTCGCTCGCAGGCCTCAACAACCGGCCATACTG

Table S19: Staple sequences for folding with 15 different staple sequences

Name	Sequence
15-different-staples_01	TCTTACGGTCGCACTTAGAATATCGCAAAAGGCCTTGTGCTATGCGCAGCAAGTGAAAACATT
15-different-staples_02	TTTATTTCGTATTCCTCATTTCCGACACCATGTCTTTT
15-different-staples_03	TTTTGCTTGAAGGCGTGTGAAGCATTAGTACTGCGTTT
15-different-staples_04	CCTGGAGGTGTCGTACTACCCTGTTCGACACGTGTCACAAAAGATTCCTTTGATAG
15-different-staples_05	TGCCTATCAACAAGACCGACATGCCAGTTACAATAGATACAATTCTGGCCAGACGATTTACCGTAAGATA
15-different-staples_06	ACGATGAAATGTAGGATAAGGCTGCATACGAGGTATAACCTAATCGCTTCTACGATGATGATCCTGGAGC
15-different-staples_07	ACATTTTGACACAAGTAAGGGTAACTGGTCAAGCTCCACTATGTTTAATCCCAAGGTGCGACC
15-different-staples_08	TTGACCACAAGAACGGGTTAGAATCGCGGTCGATTGGTCCGGTCCCCGCCTCTTCA
15-different-staples_09	TTTGGCCGTGGAGTGATTGAGAACCACGCTCATTCTTT
15-different-staples_10	CTCGCAAGAGGCATATACAAAAGGTCGTTAAGCGTTTGAATCACTACGTCTGAAAC
15-different-staples_11	AACGGCCTGTCTTCTAAGTTTGCTTACGCCAAAGAGCTAGACTGTTACTTCCATTGCCAGACT
15-different-staples_12	GTAGCCGCACCGTATAAGTTCCGAATTTTCCCTCGTGGTCCGATATCCTACCCAGGTCGCTAATTACATG
15-different-staples_13	GGCGGGTAAATCCTCAAAGTTACAGGAATAGGGCAACGCCGGCCCGGAATATCTCCACATTGGAGCCGATTGTGCGA
15-different-staples_14	CGCAAACAACTGCGATCTCCGCCACACGAAACTGATCCAGAATCAGGCGCCTGCATCGTCTAATCTCAACAGCGGTA
15-different-staples_15	ACACACAATTCTGTAACCGACTAGGATAGAGCGTTGCTAGTAGTGGCGAGAAAACCAAACATTTAGCTTACCGTTAC

Table S20: Staple sequences for folding with 20 different staple sequences

Name	Sequence
20-different-staples_01	CCCTATCCCCAAACGTACCGAGGGAATTCCAGCCTTACACAAACGAGTCCCTGATGCTATAAC

20-different-staples_02	TTTGGCAACGCATCCACGGTTCCAGAGTTGGACCTTTT
20-different-staples_03	TTTGCTGCGAGCCCTCATCAAAGGCTACATAAGTTTTT
20-different-staples_04	GAGAGCAAGTAAGTAACTCATCGGTCTTAAAATACGATTTTGAGGGTTATCCCGCA
20-different-staples_05	CCCTGTGTTATTCCGTTTATCTAAGGTCTATTGTGTGTGGGGCGCTCTGTGACTGTATATCGGATATGCA
20-different-staples_06	CTTGCCTTACTGAACGACTGCCGACCTACACGTCGCACGATCTCCGGACGCCGATATGTCGAGGTCTGCG
20-different-staples_07	ACGATTTCAAGTGCTCCGTTTCTCCCAGTCGCGATCATAACACGTACAGCGGTGCTTTCCGCT
20-different-staples_08	ATATGGCGAGCGTCGAGAATAAGCCGCCGTATAGCAATAGCCGAGGAAATCGCGTG
20-different-staples_09	TTTTGGGTGTAATGGCAAATAATCTCGTATTAAGTTTT
20-different-staples_10	TCCTACTGGTCCGTGTATCACCTTTCTGCCGCCTACCACCCCGTGAACTATCGGCG
20-different-staples_11	ACTTACCACTACCCAGCAATTATACAAATAAAATGACCCATAGGATTTAATCAAGGACGCTCA
20-different-staples_12	TCAGAGTTGCTCTACGAACCACACTTGCACCGCCGCTCCCCTTATCTCGGCCGACATAAGCCCTCGAAAT
20-different-staples_13	TTTCGATGCATATACAATCCGAGGTCTCGGCTCTTTTT
20-different-staples_14	GACCCGGCTTATGCGGCAATTCGTTCGCTTGGAACATAGGTACTGGTAGTTTCTCC
20-different-staples_15	AACCTTGGGGATTGCCCATGGCTTCACATGGCGCCGCTGGCATGGGTTACCCTGATTCTAACA
20-different-staples_16	TGCCTAATACAACTGAATTTACGATGAACCAGCCGGCGTGTACGGATATCCTCTTGTCCCCTACGCTTGA
20-different-staples_17	GAATCGCTCAGTAGCCCTCAATATAGCAGAAGAACACTTCATTTTCCCGGGATCGATTTGATTGCACCCAGCTATCG
20-different-staples_18	TAACTGAGGGAATTTTGTTTGAAACTGGGCTCCCAATATCTCCAGGCGAGCTGGGTGAGTACGTGCGCAAGTACCCT
20-different-staples_19	ATAGAAGATTTAATTAGTTACCTTTGGGCTAAACAAATAAGCAGCTCGTCGCCTGAAACATTTAAAGATGCCCTTCT
20-different-staples_20	AGACCCGCACCGGCGCAGTATTTCTTCCCTGGCGAGCAGCAGGCGGTAGCTCGGCGCCAATCTGGCCAATGCAGCCT

Scaffold sequence for Long staples with Low staple crossover density and Low scaffold crossover density (LLL) (in red = 1082bp) in M13K07 (in black=7760bp):

AACGCTACTACTATTAGTAGAATTGATGCCACCTTTTCAGCTCGCGCCCCAAATGAAAATATAGCTAAACAGGTTATTGACCATTTGCGAAATGTATCTAATGGTCAAACTAAA
TCTACTCGTTCGCAGAATTGGGAATCAACTGTTACATGGAATGAAACTTCCAGACACCGTACTTTAGTTGCATATTTAAAACATGTTGAGCTACAGCACCAGATTCAGCAATTA
AGCTCTAAGCCATCCGCAAAAATGACCTCTTATCAAAAGGAGCAATTAAAGGTACTCTCTAATCCTGACCTGTTGGAGTTTGCTTCCGGTCTGGTTCGCTTTGAAGCTCGAAT
TAAAACGCGATATTTGAAGTCTTTCGGGCTTCCTCTTAATCTTTTTGATGCAATCCGCTTTGCTTCTGACTATAATAGTCAGGGTAAAGACCTGATTTTTGATTTATGGTCATTC
TCGTTTTCTGAACTGTTTAAAGCATTTGAGGGGGATTCAATGAATATTTATGACGATTCCGCAGTATTGGACGCTATCCAGTCTAAACATTTTACTATTACCCCCTCTGGCAAA
ACTTCTTTTGCAAAAGCCTCTCGCTATTTTGGTTTTTATCGTCGTCTGGTAAACGAGGGTTATGATAGTGTTGCTCTTACTATGCCTCGTAATTCCTTTTGGCGTTATGTATCTG
CATTAGTTGAATGTGGTATTCCTAAATCTCAACTGATGAATCTTTCTACCTGTAATAATGTTGTTCCGTTAGTTCGTTTTATTAACGTAGATTTTTCTTCCCAACGTCCTGACTGG
TATAATGAGCCAGTTCTTAAAATCGCATAAGGTAATTCACAATGATTAAAGTTGAAATTAAACCATCTCAAGCCCAATTTACTACTCGTTCTGGTGTTTCTCGTCAGGGCAAGC
CTTATTCACTGAATGAGCAGCTTTGTTACGTTGATTTGGGTAATGAATATCCGGTTCTTGTCAAGATTACTCTTGATGAAGGTCAGCCAGCCTATGCGCCTGGTCTGTACACC
GTTCATCTGTCCTCTTTCAAAGTTGGTCAGTTCGGTTCCCTTATGATTGACCGTCTGCGCCTCGTTCCGGCTAAGTAACATGGAGCAGGTCGCGGATTTCGACACAATTTATC
AGGCGATGATACAAATCTCCGTTGTACTTTGTTTCGCGCTTGGTATAATCGCTGGGGGTCAAAGATGAGTGTTTTAGTGTATTCTTTCGCCTCTTTCGTTTTAGGTTGGTGCCT
TCGTAGTGGCATTACGTATTTTACCCGTTTAATGGAAACTTCCTCATGAAAAAGTCTTTAGTCCTCAAAGCCTCTGTAGCCGTTGCTACCCTCGTTCCGATGCTGTCTTTCGCT
GCTGAGGGTGACGATCCCGCAAAAGCGGCCTTTAACTCCCTGCAAGCCTCAGCGACCGAATATATCGGTTATGCGTGGGCGATGGTTGTTGTCATTGTCGGCGCAACTATC
GGTATCAAGCTGTTTAAGAAATTCACCTCGAAAGCAAGCTGATAAACCGATACAATTAAAGGCTCCTTTTGGAGCCTTTTTTTTTGGAGATTTTCAACGTGAAAAAATTATTATT
CGCAATTCCTTTAGTTGTTCCTTTCTATTCTCACTCCGCTGAAACTGTTGAAAGTTGTTTAGCAAAACCCCATACAGAAAATTCATTTACTAACGTCTGGAAAGACGACAAAACT
TTAGATCGTTACGCTAACTATGAGGGTTGTCTGTGGAATGCTACAGGCGTTGTAGTTTGTACTGGTGACGAAACTCAGTGTTACGGTACATGGGTTCCTATTGGGCTTGCTAT
CCCTGAAAATGAGGGTGGTGGCTCTGAGGGTGGCGGTTCTGAGGGTGGCGGTTCTGAGGGTGGCGGTACTAAACCTCCTGAGTACGGTGATACACCTATTCCGGGCTATA
CTTATATCAACCCTCTCGACGGCACTTATCCGCCTGGTACTGAGCAAAACCCCGCTAATCCTAATCCTTCTCTTGAGGAGTCTCAGCCTCTTAATACTTTCATGTTTCAGAATA
ATAGGTTCCGAAATAGGCAGGGGGCATTAACTGTTTATACGGGCACTGTTACTCAAGGCACTGACCCCGTTAAAACTTATTACCAGTACACTCCTGTATCATCAAAAGCCATG
TATGACGCTTACTGGAACGGTAAATTCAGAGACTGCGCTTTCCATTCTGGCTTTAATGAGGATCCATTCGTTTGTGAATATCAAGGCCAATCGTCTGACCTGCCTCAACCTCC
TGTCAATGCTGGCGGCGGCTCTGGTGGTGGTTCTGGTGGCGGCTCTGAGGGTGGTGGCTCTGAGGGTGGCGGTTCTGAGGGTGGCGGCTCTGAGGGAGGCGGTTCCGG
TGGTGGCTCTGGTTCCGGTGATTTTGATTATGAAAAGATGGCAAACGCTAATAAGGGGGCTATGACCGAAAATGCCGATGAAAACGCGCTACAGTCTGACGCTAAAGGCAAA
CTTGATTCTGTCGCTACTGATTACGGTGCTGCTATCGATGGTTTCATTGGTGACGTTTCCGGCCTTGCTAATGGTAATGGTGCTACTGGTGATTTTGCTGGCTCTAATTCCCA
AATGGCTCAAGTCGGTGACGGTGATAATTCACCTTTAATGAATAATTTCCGTCAATATTTACCTTCCCTCCCTCAATCGGTTGAATGTCGCCCTTTTGTCTTTAGCGCTGGTAA
ACCATATGAATTTTCTATTGATTGTGACAAAATAAACTTATTCCGTGGTGTCTTTGCGTTTCTTTTATATGTTGCCACCTTTATGTATGTATTTTCTACGTTTGCTAACATACTGC
GTAATAAGGAGTCTTAATCATGCCAGTTCTTTTGGGTATTCCGTTATTATTGCGTTTCCTCGGTTTCCTTCTGGTAACTTTGTTCGGCTATCTGCTTACTTTTCTTAAAAAGGGC
TTCGGTAAGATAGCTATTGCTATTTCATTGTTTCTTGCTCTTATTATTGGGCTTAACTCAATTCTTGTGGGTTATCTCTCTGATATTAGCGCTCAATTACCCTCTGACTTTGTTCA
GGGTGTTCAGTTAATTCTCCCGTCTAATGCGCTTCCCTGTTTTTATGTTATTCTCTCTGTAAAGGCTGCTATTTTCATTTTTGACGTTAAACAAAAAATCGTTTCTTATTTGGATT
GGGATAAATAATATGGCTGTTTATTTTGTAACTGGCAAATTAGGCTCTGGAAAGACGCTCGTTAGCGTTGGTAAGATTCAGGATAAAATTGTAGCTGGGTGCAAAATAGCAAC
TAATCTTGATTTAAGGCTTCAAAACCTCCCGCAAGTCGGGAGGTTCGCTAAAACGCCTCGCGTTCTTAGAATACCGGATAAGCCTTCTATATCTGATTTGCTTGCTATTGGGC
GCGGTAATGATTCCTACGATGAAAATAAAAACGGCTTGCTTGTTCTCGATGAGTGCGGTACTTGGTTTAATACCCGTTCTTGGAATGATAAGGAAAGACAGCCGATTATTGAT
TGGTTTCTACATGCTCGTAAATTAGGATGGGATATTATTTTTCTTGTTCAGGACTTATCTATTGTTGATAAACAGGCGCGTTCTGCATTAGCTGAACATGTTGTTTATTGTCGTC
GTCTGGACAGAATTACTTTACCTTTTGTCGGTACTTTATATTCTCTTATTACTGGCTCGAAAATGCCTCTGCCTAAATTACATGTTGGCGTTGTTAAATATGGCGATTCTCAATT
AAGCCCTACTGTTGAGCGTTGGCTTTATACTGGTAAGAATTTGTATAACGCATATGATACTAAACAGGCTTTTTCTAGTAATTATGATTCCGGTGTTTATTCTTATTTAACGCCT
TATTTATCACACGGTCGGTATTTCAAACCATTAAATTTAGGTCAGAAGATGAAATTAACTAAAATATATTTGAAAAAGTTTTCTCGCGTTCTTTGTCTTGCGATTGGATTTGCATC
AGCATTTACATATAGTTATATAACCCAACCTAAGCCGGAGGTTAAAAAGGTAGTCTCTCAGACCTATGATTTTGATAAATTCACTATTGACTCTTCTCAGCGTCTTAATCTAAGC
TATCGCTATGTTTTCAAGGATTCTAAGGGAAAATTAATTAATAGCGACGATTTACAGAAGCAAGGTTATTCACTCACATATATTGATTTATGTACTGTTTCCATTAAAAAAGGTA
ATTCAAATGAAATTGTTAAATGTAATTAATTTTGTTTTCTTGATGTTTGTTTCATCATCTTCTTTTGCTCAGGTAATTGAAATGAATAATTCGCCTCTGCGCGATTTTGTAACTTGG
TATTCAAAGCAATCAGGCGAATCCGTTATTGTTTCTCCCGATGTAAAAGGTACTGTTACTGTATATTCATCTGACGTTAAACCTGAAAATCTACGCAATTTCTTTATTTCTGTTTT
ACGTGCTAATAATTTTGATATGGTTGGTTCAATTCCTTCCATAATTCAGAAGTATAATCCAAACAATCAGGATTATATTGATGAATTGCCATCATCTGATAATCAGGAATATGAT
GATAATTCCGCTCCTTCTGGTGGTTTCTTTGTTCCGCAAAATGATAATGTTACTCAAACTTTTAAAATTAATAACGTTCGGGCAAAGGATTTAATACGAGTTGTCGAATTGTTTG
TAAAGTCTAATACTTCTAAATCCTCAAATGTATTATCTATTGACGGCTCTAATCTATTAGTTGTTAGTGCACCTAAAGATATTTTAGATAACCTTCCTCAATTCCTTTCTACTGTT
GATTTGCCAACTGACCAGATATTGATTGAGGGTTTGATATTTGAGGTTCAGCAAGGTGATGCTTTAGATTTTTCATTTGCTGCTGGCTCTCAGCGTGGCACTGTTGCAGGCGG
TGTTAATACTGACCGCCTCACCTCTGTTTTATCTTCTGCTGGTGGTTCGTTCGGTATTTTTAATGGCGATGTTTTAGGGCTATCAGTTCGCGCATTAAAGACTAATAGCCATTC
AAAAATATTGTCTGTGCCACGTATTCTTACGCTTTCAGGTCAGAAGGGTTCTATCTCTGTTGGCCAGAATGTCCCTTTTATTACTGGTCGTGTGACTGGTGAATCTGCCAATGT
AAATAATCCATTTCAGACGATTGAGCGTCAAAATGTAGGTATTTCCATGAGCGTTTTTCCTGTTGCAATGGCTGGCGGTAATATTGTTCTGGATATTACCAGCAAGGCCGATA
GTTTGAGTTCTTCTACTCAGGCAAGTGATGTTATTACTAATCAAAGAAGTATTGCTACAACGGTTAATTTGCGTGATGGACAGACTCTTTTACTCGGTGGCCTCACTGATTATA
AAAACACTTCTCAAGATTCTGGCGTACCGTTCCTGTCTAAAATCCCTTTAATCGGCCTCCTGTTTAGCTCCCGCTCTGATTCCAACGAGGAAAGCACGTTATACGTGCTCGTC
AAAGCAACCATAGTACGCGCCCTGTAGCGGCGCATTAAGCGCGGCGGGTGTGGTGGTTACGCGCAGCGTGACCGCTACACTTGCCAGCGCCCTAGCGCCCGCTCCTTTC
GCTTTCTTCCCTTCCTTTCTCGCCACGTTCGCCGGCTTTCCCCGTCAAGCTCTAAATCGGGGGCTCCCTTTAGGGTTCCGATTTAGTGCTTTACGGCACCTCGACCCCAAAA
AACTTGATTTGGGTGATGGTTCACGTAGTGGGCCATCGCCCTGATAGACGGTTTTTCGCCCTTTGACGTTGGAGTCCACGTTCTTTAATAGTGGACTCTTGTTCCAAACTGGA
ACAACACTCAACCCTATCTCGGGACGGATCGCTTCATGTGGCAGGAGAAAAAAGGCTGCACCGGTGCGTCAGCAGAATATGTGATACAGGATATATTCCGCTTCCTCGCTC
ACTGACTCGCTACGCTCGGTCGTTCGACTGCGGCGAGCGGAAATGGCTTACGAACGGGGCGGAGATTTCCTGGAAGATGCCAGGAAGATACTTAACAGGGAAGTGAGAGG
GCCGCGGAGGAATAGGGCGGGGCGGTATCACACGAGGCCCTGGTCTCATCATGTATGTACGACTTCCCAGAGAGCATAGACGAACCCGGACCGGCCAAGGTTTCTCCTG
GGGCGGTATCACACGAGGCCCGCTTGCGGGATGCGGCTCAGCATAGGATCTTCGGAGTGTGCCGCTTCCCACGTATCAGATGGGATGTACGACTGAGCGCGGGGTTATCT
AAAATAGGCGCATCCAGCGGAGGGTTCAGCGTTGGATTAAATCAGACCAAGCACATCTATTAATCCTTGCGGTGGACGACAGTATACGCGGACTTCCTACGCGGTAGTGTG
AGCGACACCGCTTGCGGCGGCCAGGGGTACTCCCTATTAGATGCGGAGGGTTCAGCGTTGGATTAAGGTGGACGACAGTACAGAGAGCATAGACGAACCCGGGGTTATTT
TATGCGCCTCCACGCCTACGGGCAGACCGGCCTATCCAGGATACTAGCCTAACCGGCATCTATTTTGACATAGGTTGCGGCGTGGGTAGGGGCGATGCAGAGTGGGTGAA
GGGAGTGTGAGCGACACCGCTTGCGGCGGCCAGGGGTACGGCCAGGAGATCAGTATCCGCTAAAGCCTGCGGATTCGAGGTATCCAGTATTTTGACATAGGTTGCGGCG
CGATGCAGACAAACCTTGGGCGGGCAGCGAATACTAGCCTAACCCCAGCGGCTCAGCATAGGGGAGGGAGGGATCTTCGGAGTGTGCCGCTTCCCACGTATCAACGAAC
GCCTATCATGAGGCAGTGAGATTAGTGTCCACTTATTATTTGGCATCGAATGCCAAAGAACGAAGCTCCGAGATCAGTATCCAAGCTCCGGGTAGATGCGGAGGGTTCAGC
GTTGGATTAAATCTGTACCAAAAAGACCAAGCACACGCCTACGGGCAGCGAAATGCCAAAGAACGGCTAAAGCCTGCGGATATGAGACTACCCCTAAAATAGGCGCAGACC
GTCTCGAGGAAGGTTTATCCTTGCATCAGACCAAGCACCTGAGCGCGGATGAGACTACCCTTTATGGGGGTGGGAGCGCCTCCATTGCAGGATGAGTGGGTCGACGAGCT
CGCCCCGGGGATCGACCAGTTGGTGATTTTGAACTTTTGCTTTGCCACGGAACGGTCTGCGTTGTCGGGAAGATGCGTGATCTGATCCTTCAACTCAGCAAAAGTTCGATTT
ATTCAACAAAGCCGCCGTCCCGTCAAGTCAGCGTAATGCTCTGCCAGTGTTACAACCAATTAACCAATTCTGATTAGAAAAACTCATCGAGCATCAAATGAAACTGCAATTTAT
TCATATCAGGATTATCAATACCATATTTTTGAAAAAGCCGTTTCTGTAATGAAGGAGAAAACTCACCGAGGCAGTTCCATAGGATGGCAAGATCCTGGTATCGGTCTGCGATT
CCGACTCGTCCAACATCAATACAACCTATTAATTTCCCCTCGTCAAAAATAAGGTTATCAAGTGAGAAATCACCATGAGTGACGACTGAATCCGGTGAGAATGGCAAAAGCTT
ATGCATTTCTTTCCAGACTTGTTCAACAGGCCAGCCATTACGCTCGTCATCAAAATCACTCGCATCAACCAAACCGTTATTCATTCGTGATTGCGCCTGAGCGAGACGAAATA
CGCGATCGCTGTTAAAAGGACAATTACAAACAGGAATCGAATGCAACCGGCGCAGGAACACTGCCAGCGCATCAACAATATTTTCACCTGAATCAGGATATTCTTCTAATACC
TGGAATGCTGTTTTCCCGGGGATCGCAGTGGTGAGTAACCATGCATCATCAGGAGTACGGATAAAATGCTTGATGGTCGGAAGAGGCATAAATTCCGTCAGCCAGTTTAGTC
TGACCATCTCATCTGTAACATCATTGGCAACGCTACCTTTGCCATGTTTCAGAAACAACTCTGGCGCATCGGGCTTCCCATACAATCGATAGATTGTCGCACCTGATTGCCCG
ACATTATCGCGAGCCCATTTATACCCATATAAATCAGCATCCATGTTGGAATTTAATCGCGGCCTCGAGCAAGACGTTTCCCGTTGAATATGGCTCATAACACCCCTTGTATTA
CTGTTTATGTAAGCAGACAGTTTTATTGTTCATGATGATATATTTTTATCTTGTGCAATGTAACATCAGAGATTTTGAGACACAACGTGGCTTTCCCCCCCCCCCCCTGCAGGT
CTCGGGCTATTCTTTTGATTTATAAGGGATTTTGCCGATTTCGGCCTATTGGTTAAAAAATGAGCTGATTTAACAAAAATTTAACGCGAATTTTAACAAAATATTAACGTTTACA

ATTTAAATATTTGCTTATACAATCTTCCTGTTTTTGGGGCTTTTCTGATTATCAACCGGGGTACATATGATTGACATGCTAGTTTTACGATTACCGTTCATCGATTCTCTTGTTTG
CTCCAGACTCTCAGGCAATGACCTGATAGCCTTTGTAGACCTCTCAAAAATAGCTACCCTCTCCGGCATGAATTTATCAGCTAGAACGGTTGAATATCATATTGATGGTGATTT
GACTGTCTCCGGCCTTTCTCACCCTTTTGAATCTTTACCTACACATTACTCAGGCATTGCATTTAAAATATATGAGGGTTCTAAAAATTTTTATCCTTGCGTTGAAATAAAGGCT
TCTCCCGCAAAAGTATTACAGGGTCATAATGTTTTTGGTACAACCGATTTAGCTTTATGCTCTGAGGCTTTATTGCTTAATTTTGCTAATTCTTTGCCTTGCCTGTATGATTTATT
GGATGTT

Scaffold sequence for Short staples with Low staple crossover density and Low scaffold crossover density (SLL) (in red = 1082bp) in M13K07 (in black=7760bp):

AACGCTACTACTATTAGTAGAATTGATGCCACCTTTTCAGCTCGCGCCCCAAATGAAAATATAGCTAAACAGGTTATTGACCATTTGCGAAATGTATCTAATGGTCAAACTAAA
TCTACTCGTTCGCAGAATTGGGAATCAACTGTTACATGGAATGAAACTTCCAGACACCGTACTTTAGTTGCATATTTAAAACATGTTGAGCTACAGCACCAGATTCAGCAATTA
AGCTCTAAGCCATCCGCAAAAATGACCTCTTATCAAAAGGAGCAATTAAAGGTACTCTCTAATCCTGACCTGTTGGAGTTTGCTTCCGGTCTGGTTCGCTTTGAAGCTCGAAT
TAAAACGCGATATTTGAAGTCTTTCGGGCTTCCTCTTAATCTTTTTGATGCAATCCGCTTTGCTTCTGACTATAATAGTCAGGGTAAAGACCTGATTTTTGATTTATGGTCATTC
TCGTTTTCTGAACTGTTTAAAGCATTTGAGGGGGATTCAATGAATATTTATGACGATTCCGCAGTATTGGACGCTATCCAGTCTAAACATTTTACTATTACCCCCTCTGGCAAA
ACTTCTTTTGCAAAAGCCTCTCGCTATTTTGGTTTTTATCGTCGTCTGGTAAACGAGGGTTATGATAGTGTTGCTCTTACTATGCCTCGTAATTCCTTTTGGCGTTATGTATCTG
CATTAGTTGAATGTGGTATTCCTAAATCTCAACTGATGAATCTTTCTACCTGTAATAATGTTGTTCCGTTAGTTCGTTTTATTAACGTAGATTTTTCTTCCCAACGTCCTGACTGG
TATAATGAGCCAGTTCTTAAAATCGCATAAGGTAATTCACAATGATTAAAGTTGAAATTAAACCATCTCAAGCCCAATTTACTACTCGTTCTGGTGTTTCTCGTCAGGGCAAGC
CTTATTCACTGAATGAGCAGCTTTGTTACGTTGATTTGGGTAATGAATATCCGGTTCTTGTCAAGATTACTCTTGATGAAGGTCAGCCAGCCTATGCGCCTGGTCTGTACACC
GTTCATCTGTCCTCTTTCAAAGTTGGTCAGTTCGGTTCCCTTATGATTGACCGTCTGCGCCTCGTTCCGGCTAAGTAACATGGAGCAGGTCGCGGATTTCGACACAATTTATC
AGGCGATGATACAAATCTCCGTTGTACTTTGTTTCGCGCTTGGTATAATCGCTGGGGGTCAAAGATGAGTGTTTTAGTGTATTCTTTCGCCTCTTTCGTTTTAGGTTGGTGCCT
TCGTAGTGGCATTACGTATTTTACCCGTTTAATGGAAACTTCCTCATGAAAAAGTCTTTAGTCCTCAAAGCCTCTGTAGCCGTTGCTACCCTCGTTCCGATGCTGTCTTTCGCT
GCTGAGGGTGACGATCCCGCAAAAGCGGCCTTTAACTCCCTGCAAGCCTCAGCGACCGAATATATCGGTTATGCGTGGGCGATGGTTGTTGTCATTGTCGGCGCAACTATC
GGTATCAAGCTGTTTAAGAAATTCACCTCGAAAGCAAGCTGATAAACCGATACAATTAAAGGCTCCTTTTGGAGCCTTTTTTTTTGGAGATTTTCAACGTGAAAAAATTATTATT
CGCAATTCCTTTAGTTGTTCCTTTCTATTCTCACTCCGCTGAAACTGTTGAAAGTTGTTTAGCAAAACCCCATACAGAAAATTCATTTACTAACGTCTGGAAAGACGACAAAACT
TTAGATCGTTACGCTAACTATGAGGGTTGTCTGTGGAATGCTACAGGCGTTGTAGTTTGTACTGGTGACGAAACTCAGTGTTACGGTACATGGGTTCCTATTGGGCTTGCTAT
CCCTGAAAATGAGGGTGGTGGCTCTGAGGGTGGCGGTTCTGAGGGTGGCGGTTCTGAGGGTGGCGGTACTAAACCTCCTGAGTACGGTGATACACCTATTCCGGGCTATA
CTTATATCAACCCTCTCGACGGCACTTATCCGCCTGGTACTGAGCAAAACCCCGCTAATCCTAATCCTTCTCTTGAGGAGTCTCAGCCTCTTAATACTTTCATGTTTCAGAATA
ATAGGTTCCGAAATAGGCAGGGGGCATTAACTGTTTATACGGGCACTGTTACTCAAGGCACTGACCCCGTTAAAACTTATTACCAGTACACTCCTGTATCATCAAAAGCCATG
TATGACGCTTACTGGAACGGTAAATTCAGAGACTGCGCTTTCCATTCTGGCTTTAATGAGGATCCATTCGTTTGTGAATATCAAGGCCAATCGTCTGACCTGCCTCAACCTCC
TGTCAATGCTGGCGGCGGCTCTGGTGGTGGTTCTGGTGGCGGCTCTGAGGGTGGTGGCTCTGAGGGTGGCGGTTCTGAGGGTGGCGGCTCTGAGGGAGGCGGTTCCGG
TGGTGGCTCTGGTTCCGGTGATTTTGATTATGAAAAGATGGCAAACGCTAATAAGGGGGCTATGACCGAAAATGCCGATGAAAACGCGCTACAGTCTGACGCTAAAGGCAAA
CTTGATTCTGTCGCTACTGATTACGGTGCTGCTATCGATGGTTTCATTGGTGACGTTTCCGGCCTTGCTAATGGTAATGGTGCTACTGGTGATTTTGCTGGCTCTAATTCCCA
AATGGCTCAAGTCGGTGACGGTGATAATTCACCTTTAATGAATAATTTCCGTCAATATTTACCTTCCCTCCCTCAATCGGTTGAATGTCGCCCTTTTGTCTTTAGCGCTGGTAA
ACCATATGAATTTTCTATTGATTGTGACAAAATAAACTTATTCCGTGGTGTCTTTGCGTTTCTTTTATATGTTGCCACCTTTATGTATGTATTTTCTACGTTTGCTAACATACTGC
GTAATAAGGAGTCTTAATCATGCCAGTTCTTTTGGGTATTCCGTTATTATTGCGTTTCCTCGGTTTCCTTCTGGTAACTTTGTTCGGCTATCTGCTTACTTTTCTTAAAAAGGGC
TTCGGTAAGATAGCTATTGCTATTTCATTGTTTCTTGCTCTTATTATTGGGCTTAACTCAATTCTTGTGGGTTATCTCTCTGATATTAGCGCTCAATTACCCTCTGACTTTGTTCA
GGGTGTTCAGTTAATTCTCCCGTCTAATGCGCTTCCCTGTTTTTATGTTATTCTCTCTGTAAAGGCTGCTATTTTCATTTTTGACGTTAAACAAAAAATCGTTTCTTATTTGGATT
GGGATAAATAATATGGCTGTTTATTTTGTAACTGGCAAATTAGGCTCTGGAAAGACGCTCGTTAGCGTTGGTAAGATTCAGGATAAAATTGTAGCTGGGTGCAAAATAGCAAC
TAATCTTGATTTAAGGCTTCAAAACCTCCCGCAAGTCGGGAGGTTCGCTAAAACGCCTCGCGTTCTTAGAATACCGGATAAGCCTTCTATATCTGATTTGCTTGCTATTGGGC
GCGGTAATGATTCCTACGATGAAAATAAAAACGGCTTGCTTGTTCTCGATGAGTGCGGTACTTGGTTTAATACCCGTTCTTGGAATGATAAGGAAAGACAGCCGATTATTGAT
TGGTTTCTACATGCTCGTAAATTAGGATGGGATATTATTTTTCTTGTTCAGGACTTATCTATTGTTGATAAACAGGCGCGTTCTGCATTAGCTGAACATGTTGTTTATTGTCGTC
GTCTGGACAGAATTACTTTACCTTTTGTCGGTACTTTATATTCTCTTATTACTGGCTCGAAAATGCCTCTGCCTAAATTACATGTTGGCGTTGTTAAATATGGCGATTCTCAATT
AAGCCCTACTGTTGAGCGTTGGCTTTATACTGGTAAGAATTTGTATAACGCATATGATACTAAACAGGCTTTTTCTAGTAATTATGATTCCGGTGTTTATTCTTATTTAACGCCT
TATTTATCACACGGTCGGTATTTCAAACCATTAAATTTAGGTCAGAAGATGAAATTAACTAAAATATATTTGAAAAAGTTTTCTCGCGTTCTTTGTCTTGCGATTGGATTTGCATC
AGCATTTACATATAGTTATATAACCCAACCTAAGCCGGAGGTTAAAAAGGTAGTCTCTCAGACCTATGATTTTGATAAATTCACTATTGACTCTTCTCAGCGTCTTAATCTAAGC
TATCGCTATGTTTTCAAGGATTCTAAGGGAAAATTAATTAATAGCGACGATTTACAGAAGCAAGGTTATTCACTCACATATATTGATTTATGTACTGTTTCCATTAAAAAAGGTA
ATTCAAATGAAATTGTTAAATGTAATTAATTTTGTTTTCTTGATGTTTGTTTCATCATCTTCTTTTGCTCAGGTAATTGAAATGAATAATTCGCCTCTGCGCGATTTTGTAACTTGG
TATTCAAAGCAATCAGGCGAATCCGTTATTGTTTCTCCCGATGTAAAAGGTACTGTTACTGTATATTCATCTGACGTTAAACCTGAAAATCTACGCAATTTCTTTATTTCTGTTTT
ACGTGCTAATAATTTTGATATGGTTGGTTCAATTCCTTCCATAATTCAGAAGTATAATCCAAACAATCAGGATTATATTGATGAATTGCCATCATCTGATAATCAGGAATATGAT
GATAATTCCGCTCCTTCTGGTGGTTTCTTTGTTCCGCAAAATGATAATGTTACTCAAACTTTTAAAATTAATAACGTTCGGGCAAAGGATTTAATACGAGTTGTCGAATTGTTTG
TAAAGTCTAATACTTCTAAATCCTCAAATGTATTATCTATTGACGGCTCTAATCTATTAGTTGTTAGTGCACCTAAAGATATTTTAGATAACCTTCCTCAATTCCTTTCTACTGTT
GATTTGCCAACTGACCAGATATTGATTGAGGGTTTGATATTTGAGGTTCAGCAAGGTGATGCTTTAGATTTTTCATTTGCTGCTGGCTCTCAGCGTGGCACTGTTGCAGGCGG
TGTTAATACTGACCGCCTCACCTCTGTTTTATCTTCTGCTGGTGGTTCGTTCGGTATTTTTAATGGCGATGTTTTAGGGCTATCAGTTCGCGCATTAAAGACTAATAGCCATTC
AAAAATATTGTCTGTGCCACGTATTCTTACGCTTTCAGGTCAGAAGGGTTCTATCTCTGTTGGCCAGAATGTCCCTTTTATTACTGGTCGTGTGACTGGTGAATCTGCCAATGT
AAATAATCCATTTCAGACGATTGAGCGTCAAAATGTAGGTATTTCCATGAGCGTTTTTCCTGTTGCAATGGCTGGCGGTAATATTGTTCTGGATATTACCAGCAAGGCCGATA
GTTTGAGTTCTTCTACTCAGGCAAGTGATGTTATTACTAATCAAAGAAGTATTGCTACAACGGTTAATTTGCGTGATGGACAGACTCTTTTACTCGGTGGCCTCACTGATTATA
AAAACACTTCTCAAGATTCTGGCGTACCGTTCCTGTCTAAAATCCCTTTAATCGGCCTCCTGTTTAGCTCCCGCTCTGATTCCAACGAGGAAAGCACGTTATACGTGCTCGTC
AAAGCAACCATAGTACGCGCCCTGTAGCGGCGCATTAAGCGCGGCGGGTGTGGTGGTTACGCGCAGCGTGACCGCTACACTTGCCAGCGCCCTAGCGCCCGCTCCTTTC
GCTTTCTTCCCTTCCTTTCTCGCCACGTTCGCCGGCTTTCCCCGTCAAGCTCTAAATCGGGGGCTCCCTTTAGGGTTCCGATTTAGTGCTTTACGGCACCTCGACCCCAAAA
AACTTGATTTGGGTGATGGTTCACGTAGTGGGCCATCGCCCTGATAGACGGTTTTTCGCCCTTTGACGTTGGAGTCCACGTTCTTTAATAGTGGACTCTTGTTCCAAACTGGA
ACAACACTCAACCCTATCTCGGGACGGATCGCTTCATGTGGCAGGAGAAAAAAGGCTGCACCGGTGCGTCAGCAGAATATGTGATACAGGATATATTCCGCTTCCTCGCTC
ACTGACTCGCTACGCTCGGTCGTTCGACTGCGGCGAGCGGAAATGGCTTACGAACGGGGCGGAGATTTCCTGGAAGATGCCAGGAAGATACTTAACAGGGAAGTGAGAGG
GCCGCGGAGGAATAGGGCGCCTACGGATGCCGCTGCAAATTAAGGGTTCTGAAGCGAAATGGAATGACCAAAACATGACTGGGTGACGGTACCCAACATGTTCAGCCCGA
AATGAGCAATCAGAATGAGTCCAGTTCGCAACTGACTTAGCGTGGCTACTGCGGGTCCAGTTCGATGACCCTTAGCGGATGGGATGTTGGCTACTGCGGGTTCTCGCAACT
GACGCCTACGGGATGACCCGCCTACGGATGCCGCCCTGACCCCTCACCTAGCCCTCACCTGACACAAGCCCGAAGGTCCAGTTCGATGACCCTTAGCAATCAAGCAATCA
GAATGACCAAAACATGACTGGGTGACGACTGATCGCAGTACGGCCACGACATGAGCGTTATAGTGCTAGCCCGAAATGAGCGTGGCTACTGCGGGGCCACGACATGACGG
TACCCTGACCCCGAAATGAGCAATCAGAATGACCTGACCAAGTTGTCCAGTTCATGCCGCCCAAAACATGACTGGGTGACGTGGGTAGGGGGTACAAGGTCGGGGTGAAG
GGGTGCTTCATGTTCGACACAAGCCCGAAGGTTCTCGATGACCCCAATCAGAATGAAAGGTTTTCCCTTTGCGGTGCTTAACATGAGGGCTGGTACGAAGTTCCTGACCCCT
CACCTAGCCCACCTGAAAGTTAAGGTTTTCCCTTTGCGGTGCTTCATGTTCGACACAAGGAGGGAGGGGCCCGAAGGTCCAGTTCGATGACCCTTAGCGTGGCTGGTACGC
CATCGGCCACCTGACCCCTCACCTAGCCCGAAATTCAAGACCTGGGTGACGGTACGTTCTCGCAACTGACGCCTACAAATGAGTTAGCGTGGCTACTGCGGGGCCACGAC
ATGAGCGTCTTTGTTTCCTATAGTGCGCTCAAGACGGGCTGGTACGCCTCACCTAGCCCGGGATGCCGCCCAAAACATGACTGGGTGAGCGTTATAGTGCGCGAGCAATCA
GAATGACCTGACCCCTCACCGCTCAAGACGGGCTACTGCGGCCAAAACATGACTGGGTGACGGGGTGGGAGGGTACGTTCCGCAGGATGAGTGGGTCGACGAGCTCGCC
CCGGGGATCGACCAGTTGGTGATTTTGAACTTTTGCTTTGCCACGGAACGGTCTGCGTTGTCGGGAAGATGCGTGATCTGATCCTTCAACTCAGCAAAAGTTCGATTTATTC
AACAAAGCCGCCGTCCCGTCAAGTCAGCGTAATGCTCTGCCAGTGTTACAACCAATTAACCAATTCTGATTAGAAAAACTCATCGAGCATCAAATGAAACTGCAATTTATTCAT
ATCAGGATTATCAATACCATATTTTTGAAAAAGCCGTTTCTGTAATGAAGGAGAAAACTCACCGAGGCAGTTCCATAGGATGGCAAGATCCTGGTATCGGTCTGCGATTCCGA
CTCGTCCAACATCAATACAACCTATTAATTTCCCCTCGTCAAAAATAAGGTTATCAAGTGAGAAATCACCATGAGTGACGACTGAATCCGGTGAGAATGGCAAAAGCTTATGC
ATTTCTTTCCAGACTTGTTCAACAGGCCAGCCATTACGCTCGTCATCAAAATCACTCGCATCAACCAAACCGTTATTCATTCGTGATTGCGCCTGAGCGAGACGAAATACGCG

ATCGCTGTTAAAAGGACAATTACAAACAGGAATCGAATGCAACCGGCGCAGGAACACTGCCAGCGCATCAACAATATTTTCACCTGAATCAGGATATTCTTCTAATACCTGGA
ATGCTGTTTTCCCGGGGATCGCAGTGGTGAGTAACCATGCATCATCAGGAGTACGGATAAAATGCTTGATGGTCGGAAGAGGCATAAATTCCGTCAGCCAGTTTAGTCTGAC
CATCTCATCTGTAACATCATTGGCAACGCTACCTTTGCCATGTTTCAGAAACAACTCTGGCGCATCGGGCTTCCCATACAATCGATAGATTGTCGCACCTGATTGCCCGACAT
TATCGCGAGCCCATTTATACCCATATAAATCAGCATCCATGTTGGAATTTAATCGCGGCCTCGAGCAAGACGTTTCCCGTTGAATATGGCTCATAACACCCCTTGTATTACTGT
TTATGTAAGCAGACAGTTTTATTGTTCATGATGATATATTTTTATCTTGTGCAATGTAACATCAGAGATTTTGAGACACAACGTGGCTTTCCCCCCCCCCCCCTGCAGGTCTCG
GGCTATTCTTTTGATTTATAAGGGATTTTGCCGATTTCGGCCTATTGGTTAAAAAATGAGCTGATTTAACAAAAATTTAACGCGAATTTTAACAAAATATTAACGTTTACAATTTA
AATATTTGCTTATACAATCTTCCTGTTTTTGGGGCTTTTCTGATTATCAACCGGGGTACATATGATTGACATGCTAGTTTTACGATTACCGTTCATCGATTCTCTTGTTTGCTCC
AGACTCTCAGGCAATGACCTGATAGCCTTTGTAGACCTCTCAAAAATAGCTACCCTCTCCGGCATGAATTTATCAGCTAGAACGGTTGAATATCATATTGATGGTGATTTGACT
GTCTCCGGCCTTTCTCACCCTTTTGAATCTTTACCTACACATTACTCAGGCATTGCATTTAAAATATATGAGGGTTCTAAAAATTTTTATCCTTGCGTTGAAATAAAGGCTTCTC
CCGCAAAAGTATTACAGGGTCATAATGTTTTTGGTACAACCGATTTAGCTTTATGCTCTGAGGCTTTATTGCTTAATTTTGCTAATTCTTTGCCTTGCCTGTATGATTTATTGGA
TGTT

Scaffold sequence for Short staples with High staple crossover density and Low scaffold crossover density with repetitive staple array (SHL) (in red = 1082bp) in M13K07 (in black=7760bp):

AACGCTACTACTATTAGTAGAATTGATGCCACCTTTTCAGCTCGCGCCCCAAATGAAAATATAGCTAAACAGGTTATTGACCATTTGCGAAATGTATCTAATGGTCAAACTAAA
TCTACTCGTTCGCAGAATTGGGAATCAACTGTTACATGGAATGAAACTTCCAGACACCGTACTTTAGTTGCATATTTAAAACATGTTGAGCTACAGCACCAGATTCAGCAATTA
AGCTCTAAGCCATCCGCAAAAATGACCTCTTATCAAAAGGAGCAATTAAAGGTACTCTCTAATCCTGACCTGTTGGAGTTTGCTTCCGGTCTGGTTCGCTTTGAAGCTCGAAT
TAAAACGCGATATTTGAAGTCTTTCGGGCTTCCTCTTAATCTTTTTGATGCAATCCGCTTTGCTTCTGACTATAATAGTCAGGGTAAAGACCTGATTTTTGATTTATGGTCATTC
TCGTTTTCTGAACTGTTTAAAGCATTTGAGGGGGATTCAATGAATATTTATGACGATTCCGCAGTATTGGACGCTATCCAGTCTAAACATTTTACTATTACCCCCTCTGGCAAA
ACTTCTTTTGCAAAAGCCTCTCGCTATTTTGGTTTTTATCGTCGTCTGGTAAACGAGGGTTATGATAGTGTTGCTCTTACTATGCCTCGTAATTCCTTTTGGCGTTATGTATCTG
CATTAGTTGAATGTGGTATTCCTAAATCTCAACTGATGAATCTTTCTACCTGTAATAATGTTGTTCCGTTAGTTCGTTTTATTAACGTAGATTTTTCTTCCCAACGTCCTGACTGG
TATAATGAGCCAGTTCTTAAAATCGCATAAGGTAATTCACAATGATTAAAGTTGAAATTAAACCATCTCAAGCCCAATTTACTACTCGTTCTGGTGTTTCTCGTCAGGGCAAGC
CTTATTCACTGAATGAGCAGCTTTGTTACGTTGATTTGGGTAATGAATATCCGGTTCTTGTCAAGATTACTCTTGATGAAGGTCAGCCAGCCTATGCGCCTGGTCTGTACACC
GTTCATCTGTCCTCTTTCAAAGTTGGTCAGTTCGGTTCCCTTATGATTGACCGTCTGCGCCTCGTTCCGGCTAAGTAACATGGAGCAGGTCGCGGATTTCGACACAATTTATC
AGGCGATGATACAAATCTCCGTTGTACTTTGTTTCGCGCTTGGTATAATCGCTGGGGGTCAAAGATGAGTGTTTTAGTGTATTCTTTCGCCTCTTTCGTTTTAGGTTGGTGCCT
TCGTAGTGGCATTACGTATTTTACCCGTTTAATGGAAACTTCCTCATGAAAAAGTCTTTAGTCCTCAAAGCCTCTGTAGCCGTTGCTACCCTCGTTCCGATGCTGTCTTTCGCT
GCTGAGGGTGACGATCCCGCAAAAGCGGCCTTTAACTCCCTGCAAGCCTCAGCGACCGAATATATCGGTTATGCGTGGGCGATGGTTGTTGTCATTGTCGGCGCAACTATC
GGTATCAAGCTGTTTAAGAAATTCACCTCGAAAGCAAGCTGATAAACCGATACAATTAAAGGCTCCTTTTGGAGCCTTTTTTTTTGGAGATTTTCAACGTGAAAAAATTATTATT
CGCAATTCCTTTAGTTGTTCCTTTCTATTCTCACTCCGCTGAAACTGTTGAAAGTTGTTTAGCAAAACCCCATACAGAAAATTCATTTACTAACGTCTGGAAAGACGACAAAACT
TTAGATCGTTACGCTAACTATGAGGGTTGTCTGTGGAATGCTACAGGCGTTGTAGTTTGTACTGGTGACGAAACTCAGTGTTACGGTACATGGGTTCCTATTGGGCTTGCTAT
CCCTGAAAATGAGGGTGGTGGCTCTGAGGGTGGCGGTTCTGAGGGTGGCGGTTCTGAGGGTGGCGGTACTAAACCTCCTGAGTACGGTGATACACCTATTCCGGGCTATA
CTTATATCAACCCTCTCGACGGCACTTATCCGCCTGGTACTGAGCAAAACCCCGCTAATCCTAATCCTTCTCTTGAGGAGTCTCAGCCTCTTAATACTTTCATGTTTCAGAATA
ATAGGTTCCGAAATAGGCAGGGGGCATTAACTGTTTATACGGGCACTGTTACTCAAGGCACTGACCCCGTTAAAACTTATTACCAGTACACTCCTGTATCATCAAAAGCCATG
TATGACGCTTACTGGAACGGTAAATTCAGAGACTGCGCTTTCCATTCTGGCTTTAATGAGGATCCATTCGTTTGTGAATATCAAGGCCAATCGTCTGACCTGCCTCAACCTCC
TGTCAATGCTGGCGGCGGCTCTGGTGGTGGTTCTGGTGGCGGCTCTGAGGGTGGTGGCTCTGAGGGTGGCGGTTCTGAGGGTGGCGGCTCTGAGGGAGGCGGTTCCGG
TGGTGGCTCTGGTTCCGGTGATTTTGATTATGAAAAGATGGCAAACGCTAATAAGGGGGCTATGACCGAAAATGCCGATGAAAACGCGCTACAGTCTGACGCTAAAGGCAAA
CTTGATTCTGTCGCTACTGATTACGGTGCTGCTATCGATGGTTTCATTGGTGACGTTTCCGGCCTTGCTAATGGTAATGGTGCTACTGGTGATTTTGCTGGCTCTAATTCCCA
AATGGCTCAAGTCGGTGACGGTGATAATTCACCTTTAATGAATAATTTCCGTCAATATTTACCTTCCCTCCCTCAATCGGTTGAATGTCGCCCTTTTGTCTTTAGCGCTGGTAA
ACCATATGAATTTTCTATTGATTGTGACAAAATAAACTTATTCCGTGGTGTCTTTGCGTTTCTTTTATATGTTGCCACCTTTATGTATGTATTTTCTACGTTTGCTAACATACTGC
GTAATAAGGAGTCTTAATCATGCCAGTTCTTTTGGGTATTCCGTTATTATTGCGTTTCCTCGGTTTCCTTCTGGTAACTTTGTTCGGCTATCTGCTTACTTTTCTTAAAAAGGGC
TTCGGTAAGATAGCTATTGCTATTTCATTGTTTCTTGCTCTTATTATTGGGCTTAACTCAATTCTTGTGGGTTATCTCTCTGATATTAGCGCTCAATTACCCTCTGACTTTGTTCA
GGGTGTTCAGTTAATTCTCCCGTCTAATGCGCTTCCCTGTTTTTATGTTATTCTCTCTGTAAAGGCTGCTATTTTCATTTTTGACGTTAAACAAAAAATCGTTTCTTATTTGGATT
GGGATAAATAATATGGCTGTTTATTTTGTAACTGGCAAATTAGGCTCTGGAAAGACGCTCGTTAGCGTTGGTAAGATTCAGGATAAAATTGTAGCTGGGTGCAAAATAGCAAC
TAATCTTGATTTAAGGCTTCAAAACCTCCCGCAAGTCGGGAGGTTCGCTAAAACGCCTCGCGTTCTTAGAATACCGGATAAGCCTTCTATATCTGATTTGCTTGCTATTGGGC
GCGGTAATGATTCCTACGATGAAAATAAAAACGGCTTGCTTGTTCTCGATGAGTGCGGTACTTGGTTTAATACCCGTTCTTGGAATGATAAGGAAAGACAGCCGATTATTGAT
TGGTTTCTACATGCTCGTAAATTAGGATGGGATATTATTTTTCTTGTTCAGGACTTATCTATTGTTGATAAACAGGCGCGTTCTGCATTAGCTGAACATGTTGTTTATTGTCGTC
GTCTGGACAGAATTACTTTACCTTTTGTCGGTACTTTATATTCTCTTATTACTGGCTCGAAAATGCCTCTGCCTAAATTACATGTTGGCGTTGTTAAATATGGCGATTCTCAATT
AAGCCCTACTGTTGAGCGTTGGCTTTATACTGGTAAGAATTTGTATAACGCATATGATACTAAACAGGCTTTTTCTAGTAATTATGATTCCGGTGTTTATTCTTATTTAACGCCT
TATTTATCACACGGTCGGTATTTCAAACCATTAAATTTAGGTCAGAAGATGAAATTAACTAAAATATATTTGAAAAAGTTTTCTCGCGTTCTTTGTCTTGCGATTGGATTTGCATC
AGCATTTACATATAGTTATATAACCCAACCTAAGCCGGAGGTTAAAAAGGTAGTCTCTCAGACCTATGATTTTGATAAATTCACTATTGACTCTTCTCAGCGTCTTAATCTAAGC
TATCGCTATGTTTTCAAGGATTCTAAGGGAAAATTAATTAATAGCGACGATTTACAGAAGCAAGGTTATTCACTCACATATATTGATTTATGTACTGTTTCCATTAAAAAAGGTA
ATTCAAATGAAATTGTTAAATGTAATTAATTTTGTTTTCTTGATGTTTGTTTCATCATCTTCTTTTGCTCAGGTAATTGAAATGAATAATTCGCCTCTGCGCGATTTTGTAACTTGG
TATTCAAAGCAATCAGGCGAATCCGTTATTGTTTCTCCCGATGTAAAAGGTACTGTTACTGTATATTCATCTGACGTTAAACCTGAAAATCTACGCAATTTCTTTATTTCTGTTTT
ACGTGCTAATAATTTTGATATGGTTGGTTCAATTCCTTCCATAATTCAGAAGTATAATCCAAACAATCAGGATTATATTGATGAATTGCCATCATCTGATAATCAGGAATATGAT
GATAATTCCGCTCCTTCTGGTGGTTTCTTTGTTCCGCAAAATGATAATGTTACTCAAACTTTTAAAATTAATAACGTTCGGGCAAAGGATTTAATACGAGTTGTCGAATTGTTTG
TAAAGTCTAATACTTCTAAATCCTCAAATGTATTATCTATTGACGGCTCTAATCTATTAGTTGTTAGTGCACCTAAAGATATTTTAGATAACCTTCCTCAATTCCTTTCTACTGTT
GATTTGCCAACTGACCAGATATTGATTGAGGGTTTGATATTTGAGGTTCAGCAAGGTGATGCTTTAGATTTTTCATTTGCTGCTGGCTCTCAGCGTGGCACTGTTGCAGGCGG
TGTTAATACTGACCGCCTCACCTCTGTTTTATCTTCTGCTGGTGGTTCGTTCGGTATTTTTAATGGCGATGTTTTAGGGCTATCAGTTCGCGCATTAAAGACTAATAGCCATTC
AAAAATATTGTCTGTGCCACGTATTCTTACGCTTTCAGGTCAGAAGGGTTCTATCTCTGTTGGCCAGAATGTCCCTTTTATTACTGGTCGTGTGACTGGTGAATCTGCCAATGT
AAATAATCCATTTCAGACGATTGAGCGTCAAAATGTAGGTATTTCCATGAGCGTTTTTCCTGTTGCAATGGCTGGCGGTAATATTGTTCTGGATATTACCAGCAAGGCCGATA
GTTTGAGTTCTTCTACTCAGGCAAGTGATGTTATTACTAATCAAAGAAGTATTGCTACAACGGTTAATTTGCGTGATGGACAGACTCTTTTACTCGGTGGCCTCACTGATTATA
AAAACACTTCTCAAGATTCTGGCGTACCGTTCCTGTCTAAAATCCCTTTAATCGGCCTCCTGTTTAGCTCCCGCTCTGATTCCAACGAGGAAAGCACGTTATACGTGCTCGTC
AAAGCAACCATAGTACGCGCCCTGTAGCGGCGCATTAAGCGCGGCGGGTGTGGTGGTTACGCGCAGCGTGACCGCTACACTTGCCAGCGCCCTAGCGCCCGCTCCTTTC
GCTTTCTTCCCTTCCTTTCTCGCCACGTTCGCCGGCTTTCCCCGTCAAGCTCTAAATCGGGGGCTCCCTTTAGGGTTCCGATTTAGTGCTTTACGGCACCTCGACCCCAAAA
AACTTGATTTGGGTGATGGTTCACGTAGTGGGCCATCGCCCTGATAGACGGTTTTTCGCCCTTTGACGTTGGAGTCCACGTTCTTTAATAGTGGACTCTTGTTCCAAACTGGA
ACAACACTCAACCCTATCTCGGGACGGATCGCTTCATGTGGCAGGAGAAAAAAGGCTGCACCGGTGCGTCAGCAGAATATGTGATACAGGATATATTCCGCTTCCTCGCTC
ACTGACTCGCTACGCTCGGTCGTTCGACTGCGGCGAGCGGAAATGGCTTACGAACGGGGCGGAGATTTCCTGGAAGATGCCAGGAAGATACTTAACAGGGAAGTGAGAGG
GCCGCGGAGGAATAGGGCGTCGCAATATGTTAATTTCAAGCAATTACCCGATCTCTGGATTGGCCACAATGTTAATTTCAAGCAATTACCCGATCTCTGGATTGGCCACAAT
GTTAATTTCAAGCAATTACCCGATCTCTGGATTGGCCACAATGTTAATTTCAAGCAATAACCAAAACTGGCGTCTGATGGGATGTTGGGACGTGTGGCTTTATTCGGGCCACA
CTGGGGATTTCAAGTTCCCTTCAACAAGTTATTCGGGCCACACTGGGGATTTCAAGTTCCCTTCAACAAGTTATTCGGGCCACACTGGGGATTTCAAGTTCCCTTCAACAAGT
TATTCGGGCCACACTGGGGAACTTTGTCACCGCCTCCCGAGGTGTCACACTATCTAGTTTGAACAGCACGAACGCCTCCCGAGGTGTCACACTATCTAGTTTGAACAGCAC
GAACGCCTCCCGAGGTGTCACACTATCTAGTTTGAACAGCACGAACGCCTCCCGAGGTGTCACACTAAATGCATGGGTAGGGGGCCTCGACAGAGGGTGAAGGGTTGAAC
AGCACGAAGTATCCGTCACGGCTCTCTCACCATCGATTGAACAGCACGAAGTATCCGTCACGGCTCTCTCACCATCGATTGAACAGCACGAAGTATCCGTCACGGCTCTCTC
ACCATCGATTGAACAGCACGAAGTATCCGTCACGGCTCTATGTTAAGTATGGAGGGAGGGCCGCTCTTGGGCCGCTATCAACTCTTGGGCCGATCCCTTAGATGAAGAGCG
GCTGCCTATCAACTCTTGGGCCGATCCCTTAGATGAAGAGCGGCTGCCTATCAACTCTTGGGCCGATCCCTTAGATGAAGAGCGGCTGCCTATCAACTCTTGGGCCGATCC

CTTAGCTGCCCGTGGTTATAAACACTTCGTCCTTAGAGCCTGAGATGCTGCCCGTGGTTATAAACACTTCGTCCTTAGAGCCTGAGATGCTGCCCGTGGTTATAAACACTTC
GTCCTTAGAGCCTGAGATGCTGCCCGTGGTTATAAACACTTCGTATCCCGTGGGGTGGGAGGGTCGAGGTATCAGGATGAGTGGGTCGACGAGCTCGCCCCGGGGATCGA
CCAGTTGGTGATTTTGAACTTTTGCTTTGCCACGGAACGGTCTGCGTTGTCGGGAAGATGCGTGATCTGATCCTTCAACTCAGCAAAAGTTCGATTTATTCAACAAAGCCGCC
GTCCCGTCAAGTCAGCGTAATGCTCTGCCAGTGTTACAACCAATTAACCAATTCTGATTAGAAAAACTCATCGAGCATCAAATGAAACTGCAATTTATTCATATCAGGATTATC
AATACCATATTTTTGAAAAAGCCGTTTCTGTAATGAAGGAGAAAACTCACCGAGGCAGTTCCATAGGATGGCAAGATCCTGGTATCGGTCTGCGATTCCGACTCGTCCAACAT
CAATACAACCTATTAATTTCCCCTCGTCAAAAATAAGGTTATCAAGTGAGAAATCACCATGAGTGACGACTGAATCCGGTGAGAATGGCAAAAGCTTATGCATTTCTTTCCAGA
CTTGTTCAACAGGCCAGCCATTACGCTCGTCATCAAAATCACTCGCATCAACCAAACCGTTATTCATTCGTGATTGCGCCTGAGCGAGACGAAATACGCGATCGCTGTTAAAA
GGACAATTACAAACAGGAATCGAATGCAACCGGCGCAGGAACACTGCCAGCGCATCAACAATATTTTCACCTGAATCAGGATATTCTTCTAATACCTGGAATGCTGTTTTCCC
GGGGATCGCAGTGGTGAGTAACCATGCATCATCAGGAGTACGGATAAAATGCTTGATGGTCGGAAGAGGCATAAATTCCGTCAGCCAGTTTAGTCTGACCATCTCATCTGTA
ACATCATTGGCAACGCTACCTTTGCCATGTTTCAGAAACAACTCTGGCGCATCGGGCTTCCCATACAATCGATAGATTGTCGCACCTGATTGCCCGACATTATCGCGAGCCC
ATTTATACCCATATAAATCAGCATCCATGTTGGAATTTAATCGCGGCCTCGAGCAAGACGTTTCCCGTTGAATATGGCTCATAACACCCCTTGTATTACTGTTTATGTAAGCAG
ACAGTTTTATTGTTCATGATGATATATTTTTATCTTGTGCAATGTAACATCAGAGATTTTGAGACACAACGTGGCTTTCCCCCCCCCCCCCTGCAGGTCTCGGGCTATTCTTTT
GATTTATAAGGGATTTTGCCGATTTCGGCCTATTGGTTAAAAAATGAGCTGATTTAACAAAAATTTAACGCGAATTTTAACAAAATATTAACGTTTACAATTTAAATATTTGCTTA
TACAATCTTCCTGTTTTTGGGGCTTTTCTGATTATCAACCGGGGTACATATGATTGACATGCTAGTTTTACGATTACCGTTCATCGATTCTCTTGTTTGCTCCAGACTCTCAGG
CAATGACCTGATAGCCTTTGTAGACCTCTCAAAAATAGCTACCCTCTCCGGCATGAATTTATCAGCTAGAACGGTTGAATATCATATTGATGGTGATTTGACTGTCTCCGGCCT
TTCTCACCCTTTTGAATCTTTACCTACACATTACTCAGGCATTGCATTTAAAATATATGAGGGTTCTAAAAATTTTTATCCTTGCGTTGAAATAAAGGCTTCTCCCGCAAAAGTAT
TACAGGGTCATAATGTTTTTGGTACAACCGATTTAGCTTTATGCTCTGAGGCTTTATTGCTTAATTTTGCTAATTCTTTGCCTTGCCTGTATGATTTATTGGATGTT

Scaffold sequence for Long staples with Low staple crossover density and High scaffold crossover density (LLH) (in red = 1082bp) in M13K07 (in black=7760bp) - The same scaffold sequence was used for Short staples with Low staple crossover density and High scaffold crossover density (SLH):

AACGCTACTACTATTAGTAGAATTGATGCCACCTTTTCAGCTCGCGCCCCAAATGAAAATATAGCTAAACAGGTTATTGACCATTTGCGAAATGTATCTAATGGTCAAACTAAA
TCTACTCGTTCGCAGAATTGGGAATCAACTGTTACATGGAATGAAACTTCCAGACACCGTACTTTAGTTGCATATTTAAAACATGTTGAGCTACAGCACCAGATTCAGCAATTA
AGCTCTAAGCCATCCGCAAAAATGACCTCTTATCAAAAGGAGCAATTAAAGGTACTCTCTAATCCTGACCTGTTGGAGTTTGCTTCCGGTCTGGTTCGCTTTGAAGCTCGAAT
TAAAACGCGATATTTGAAGTCTTTCGGGCTTCCTCTTAATCTTTTTGATGCAATCCGCTTTGCTTCTGACTATAATAGTCAGGGTAAAGACCTGATTTTTGATTTATGGTCATTC
TCGTTTTCTGAACTGTTTAAAGCATTTGAGGGGGATTCAATGAATATTTATGACGATTCCGCAGTATTGGACGCTATCCAGTCTAAACATTTTACTATTACCCCCTCTGGCAAA
ACTTCTTTTGCAAAAGCCTCTCGCTATTTTGGTTTTTATCGTCGTCTGGTAAACGAGGGTTATGATAGTGTTGCTCTTACTATGCCTCGTAATTCCTTTTGGCGTTATGTATCTG
CATTAGTTGAATGTGGTATTCCTAAATCTCAACTGATGAATCTTTCTACCTGTAATAATGTTGTTCCGTTAGTTCGTTTTATTAACGTAGATTTTTCTTCCCAACGTCCTGACTGG
TATAATGAGCCAGTTCTTAAAATCGCATAAGGTAATTCACAATGATTAAAGTTGAAATTAAACCATCTCAAGCCCAATTTACTACTCGTTCTGGTGTTTCTCGTCAGGGCAAGC
CTTATTCACTGAATGAGCAGCTTTGTTACGTTGATTTGGGTAATGAATATCCGGTTCTTGTCAAGATTACTCTTGATGAAGGTCAGCCAGCCTATGCGCCTGGTCTGTACACC
GTTCATCTGTCCTCTTTCAAAGTTGGTCAGTTCGGTTCCCTTATGATTGACCGTCTGCGCCTCGTTCCGGCTAAGTAACATGGAGCAGGTCGCGGATTTCGACACAATTTATC
AGGCGATGATACAAATCTCCGTTGTACTTTGTTTCGCGCTTGGTATAATCGCTGGGGGTCAAAGATGAGTGTTTTAGTGTATTCTTTCGCCTCTTTCGTTTTAGGTTGGTGCCT
TCGTAGTGGCATTACGTATTTTACCCGTTTAATGGAAACTTCCTCATGAAAAAGTCTTTAGTCCTCAAAGCCTCTGTAGCCGTTGCTACCCTCGTTCCGATGCTGTCTTTCGCT
GCTGAGGGTGACGATCCCGCAAAAGCGGCCTTTAACTCCCTGCAAGCCTCAGCGACCGAATATATCGGTTATGCGTGGGCGATGGTTGTTGTCATTGTCGGCGCAACTATC
GGTATCAAGCTGTTTAAGAAATTCACCTCGAAAGCAAGCTGATAAACCGATACAATTAAAGGCTCCTTTTGGAGCCTTTTTTTTTGGAGATTTTCAACGTGAAAAAATTATTATT
CGCAATTCCTTTAGTTGTTCCTTTCTATTCTCACTCCGCTGAAACTGTTGAAAGTTGTTTAGCAAAACCCCATACAGAAAATTCATTTACTAACGTCTGGAAAGACGACAAAACT
TTAGATCGTTACGCTAACTATGAGGGTTGTCTGTGGAATGCTACAGGCGTTGTAGTTTGTACTGGTGACGAAACTCAGTGTTACGGTACATGGGTTCCTATTGGGCTTGCTAT
CCCTGAAAATGAGGGTGGTGGCTCTGAGGGTGGCGGTTCTGAGGGTGGCGGTTCTGAGGGTGGCGGTACTAAACCTCCTGAGTACGGTGATACACCTATTCCGGGCTATA
CTTATATCAACCCTCTCGACGGCACTTATCCGCCTGGTACTGAGCAAAACCCCGCTAATCCTAATCCTTCTCTTGAGGAGTCTCAGCCTCTTAATACTTTCATGTTTCAGAATA
ATAGGTTCCGAAATAGGCAGGGGGCATTAACTGTTTATACGGGCACTGTTACTCAAGGCACTGACCCCGTTAAAACTTATTACCAGTACACTCCTGTATCATCAAAAGCCATG
TATGACGCTTACTGGAACGGTAAATTCAGAGACTGCGCTTTCCATTCTGGCTTTAATGAGGATCCATTCGTTTGTGAATATCAAGGCCAATCGTCTGACCTGCCTCAACCTCC
TGTCAATGCTGGCGGCGGCTCTGGTGGTGGTTCTGGTGGCGGCTCTGAGGGTGGTGGCTCTGAGGGTGGCGGTTCTGAGGGTGGCGGCTCTGAGGGAGGCGGTTCCGG
TGGTGGCTCTGGTTCCGGTGATTTTGATTATGAAAAGATGGCAAACGCTAATAAGGGGGCTATGACCGAAAATGCCGATGAAAACGCGCTACAGTCTGACGCTAAAGGCAAA
CTTGATTCTGTCGCTACTGATTACGGTGCTGCTATCGATGGTTTCATTGGTGACGTTTCCGGCCTTGCTAATGGTAATGGTGCTACTGGTGATTTTGCTGGCTCTAATTCCCA
AATGGCTCAAGTCGGTGACGGTGATAATTCACCTTTAATGAATAATTTCCGTCAATATTTACCTTCCCTCCCTCAATCGGTTGAATGTCGCCCTTTTGTCTTTAGCGCTGGTAA
ACCATATGAATTTTCTATTGATTGTGACAAAATAAACTTATTCCGTGGTGTCTTTGCGTTTCTTTTATATGTTGCCACCTTTATGTATGTATTTTCTACGTTTGCTAACATACTGC
GTAATAAGGAGTCTTAATCATGCCAGTTCTTTTGGGTATTCCGTTATTATTGCGTTTCCTCGGTTTCCTTCTGGTAACTTTGTTCGGCTATCTGCTTACTTTTCTTAAAAAGGGC
TTCGGTAAGATAGCTATTGCTATTTCATTGTTTCTTGCTCTTATTATTGGGCTTAACTCAATTCTTGTGGGTTATCTCTCTGATATTAGCGCTCAATTACCCTCTGACTTTGTTCA
GGGTGTTCAGTTAATTCTCCCGTCTAATGCGCTTCCCTGTTTTTATGTTATTCTCTCTGTAAAGGCTGCTATTTTCATTTTTGACGTTAAACAAAAAATCGTTTCTTATTTGGATT
GGGATAAATAATATGGCTGTTTATTTTGTAACTGGCAAATTAGGCTCTGGAAAGACGCTCGTTAGCGTTGGTAAGATTCAGGATAAAATTGTAGCTGGGTGCAAAATAGCAAC
TAATCTTGATTTAAGGCTTCAAAACCTCCCGCAAGTCGGGAGGTTCGCTAAAACGCCTCGCGTTCTTAGAATACCGGATAAGCCTTCTATATCTGATTTGCTTGCTATTGGGC
GCGGTAATGATTCCTACGATGAAAATAAAAACGGCTTGCTTGTTCTCGATGAGTGCGGTACTTGGTTTAATACCCGTTCTTGGAATGATAAGGAAAGACAGCCGATTATTGAT
TGGTTTCTACATGCTCGTAAATTAGGATGGGATATTATTTTTCTTGTTCAGGACTTATCTATTGTTGATAAACAGGCGCGTTCTGCATTAGCTGAACATGTTGTTTATTGTCGTC
GTCTGGACAGAATTACTTTACCTTTTGTCGGTACTTTATATTCTCTTATTACTGGCTCGAAAATGCCTCTGCCTAAATTACATGTTGGCGTTGTTAAATATGGCGATTCTCAATT
AAGCCCTACTGTTGAGCGTTGGCTTTATACTGGTAAGAATTTGTATAACGCATATGATACTAAACAGGCTTTTTCTAGTAATTATGATTCCGGTGTTTATTCTTATTTAACGCCT
TATTTATCACACGGTCGGTATTTCAAACCATTAAATTTAGGTCAGAAGATGAAATTAACTAAAATATATTTGAAAAAGTTTTCTCGCGTTCTTTGTCTTGCGATTGGATTTGCATC
AGCATTTACATATAGTTATATAACCCAACCTAAGCCGGAGGTTAAAAAGGTAGTCTCTCAGACCTATGATTTTGATAAATTCACTATTGACTCTTCTCAGCGTCTTAATCTAAGC
TATCGCTATGTTTTCAAGGATTCTAAGGGAAAATTAATTAATAGCGACGATTTACAGAAGCAAGGTTATTCACTCACATATATTGATTTATGTACTGTTTCCATTAAAAAAGGTA
ATTCAAATGAAATTGTTAAATGTAATTAATTTTGTTTTCTTGATGTTTGTTTCATCATCTTCTTTTGCTCAGGTAATTGAAATGAATAATTCGCCTCTGCGCGATTTTGTAACTTGG
TATTCAAAGCAATCAGGCGAATCCGTTATTGTTTCTCCCGATGTAAAAGGTACTGTTACTGTATATTCATCTGACGTTAAACCTGAAAATCTACGCAATTTCTTTATTTCTGTTTT
ACGTGCTAATAATTTTGATATGGTTGGTTCAATTCCTTCCATAATTCAGAAGTATAATCCAAACAATCAGGATTATATTGATGAATTGCCATCATCTGATAATCAGGAATATGAT
GATAATTCCGCTCCTTCTGGTGGTTTCTTTGTTCCGCAAAATGATAATGTTACTCAAACTTTTAAAATTAATAACGTTCGGGCAAAGGATTTAATACGAGTTGTCGAATTGTTTG
TAAAGTCTAATACTTCTAAATCCTCAAATGTATTATCTATTGACGGCTCTAATCTATTAGTTGTTAGTGCACCTAAAGATATTTTAGATAACCTTCCTCAATTCCTTTCTACTGTT
GATTTGCCAACTGACCAGATATTGATTGAGGGTTTGATATTTGAGGTTCAGCAAGGTGATGCTTTAGATTTTTCATTTGCTGCTGGCTCTCAGCGTGGCACTGTTGCAGGCGG
TGTTAATACTGACCGCCTCACCTCTGTTTTATCTTCTGCTGGTGGTTCGTTCGGTATTTTTAATGGCGATGTTTTAGGGCTATCAGTTCGCGCATTAAAGACTAATAGCCATTC
AAAAATATTGTCTGTGCCACGTATTCTTACGCTTTCAGGTCAGAAGGGTTCTATCTCTGTTGGCCAGAATGTCCCTTTTATTACTGGTCGTGTGACTGGTGAATCTGCCAATGT
AAATAATCCATTTCAGACGATTGAGCGTCAAAATGTAGGTATTTCCATGAGCGTTTTTCCTGTTGCAATGGCTGGCGGTAATATTGTTCTGGATATTACCAGCAAGGCCGATA
GTTTGAGTTCTTCTACTCAGGCAAGTGATGTTATTACTAATCAAAGAAGTATTGCTACAACGGTTAATTTGCGTGATGGACAGACTCTTTTACTCGGTGGCCTCACTGATTATA
AAAACACTTCTCAAGATTCTGGCGTACCGTTCCTGTCTAAAATCCCTTTAATCGGCCTCCTGTTTAGCTCCCGCTCTGATTCCAACGAGGAAAGCACGTTATACGTGCTCGTC
AAAGCAACCATAGTACGCGCCCTGTAGCGGCGCATTAAGCGCGGCGGGTGTGGTGGTTACGCGCAGCGTGACCGCTACACTTGCCAGCGCCCTAGCGCCCGCTCCTTTC
GCTTTCTTCCCTTCCTTTCTCGCCACGTTCGCCGGCTTTCCCCGTCAAGCTCTAAATCGGGGGCTCCCTTTAGGGTTCCGATTTAGTGCTTTACGGCACCTCGACCCCAAAA
AACTTGATTTGGGTGATGGTTCACGTAGTGGGCCATCGCCCTGATAGACGGTTTTTCGCCCTTTGACGTTGGAGTCCACGTTCTTTAATAGTGGACTCTTGTTCCAAACTGGA
ACAACACTCAACCCTATCTCGGGACGGATCGCTTCATGTGGCAGGAGAAAAAAGGCTGCACCGGTGCGTCAGCAGAATATGTGATACAGGATATATTCCGCTTCCTCGCTC

ACTGACTCGCTACGCTCGGTCGTTCGACTGCGGCGAGCGGAAATGGCTTACGAACGGGGCGGAGATTTCCTGGAAGATGCCAGGAAGATACTTAACAGGGAAGTGAGAGG
GCCGCGGAGGAATAGGGCGACGTTAATAGCGATGGTAGTATCGTTATCCACCTGCCAATCGTGTTATGATACAGAGGAGCGACCTCACGTTTAGGTGTCACGCCGTCAGAC
CTTTGCTACGCGGCGTTCGCCATACGTAGGGTAAAGGTCACGCAGCCATCTGAGGCGACGGTATGATACCAGGAAAGGTAGCGGCCCACGTGTCCCGTTCACTGTTACAG
TTCCACTTCGAAATATCACCCCGTGTTTCCTACCTCATTTGTTAGAAGGAATAAGTGTAATGGCGAGGTACGATGCTAGTAGAGGCTTTGGGCCATCGTTTTGCTATCGGTAG
CACACGGTGTCAGGACCCCGAGTCGCTGTACCATAAGGCTCTCCGCGACGCGACTGGGATAGTGATGGGATGTGTTGGGAGGGATGGGTAGGGGGGGTACGGCGAGGG
TGAAGGGGAAGGAGTTAAAGGTCAGGGTAGCAAACGATGGTCACACCAACTCGTTCGATCCACCAAGGGCGGGAACAAAAGGTCAGGACAGACTTCGATTTCGAGGTTAAT
AGCGATGGGATAGCACCTTTACCTGCCGCGAGGGTAGCAAACGATGGTCGCTGTACCATAAGGCTGAGGTCACCCCGTCGTTTGCGAGCTTGTAATAACCTAAATACTAAG
CACACGTTAATAGCGATGGGAACAAATGATACTCCGAGATGCTAGTAACTCACACCGACTGGGAACAATGTCGTCGAAAAGGTCAGGAATGAATTGAATCAGTGCTAGTCCC
CAAGGAGAACTCACACCAGCCATCGTTGAGTTCGTTTTGCTACCTCAGGGACTCACCTCCTGCGGAACACCTGATCATAATCCATCTTTCTACACGGCCCAAAACCATATTAC
TGCACTTCGGTAAAGTCAGATACCTAAATACCGCGAGGGGCGTCAGACCTGAGTTCTTTAGCAGGAGGAGGCGACGGTACCATAAGGCTGAGGGTACGGCGAGCAAACGA
TGGTCGTTCGTGCGGGGATAGTGTTGGGAGGCTCCGGGAGGGAGGGCGAGGGTAGTCGGGGTGGGAGACCCCGAGAACCAGGATGAGTGGGTCGACGAGCTCGCCCC
GGGGATCGACCAGTTGGTGATTTTGAACTTTTGCTTTGCCACGGAACGGTCTGCGTTGTCGGGAAGATGCGTGATCTGATCCTTCAACTCAGCAAAAGTTCGATTTATTCAAC
AAAGCCGCCGTCCCGTCAAGTCAGCGTAATGCTCTGCCAGTGTTACAACCAATTAACCAATTCTGATTAGAAAAACTCATCGAGCATCAAATGAAACTGCAATTTATTCATATC
AGGATTATCAATACCATATTTTTGAAAAAGCCGTTTCTGTAATGAAGGAGAAAACTCACCGAGGCAGTTCCATAGGATGGCAAGATCCTGGTATCGGTCTGCGATTCCGACTC
GTCCAACATCAATACAACCTATTAATTTCCCCTCGTCAAAAATAAGGTTATCAAGTGAGAAATCACCATGAGTGACGACTGAATCCGGTGAGAATGGCAAAAGCTTATGCATTT
CTTTCCAGACTTGTTCAACAGGCCAGCCATTACGCTCGTCATCAAAATCACTCGCATCAACCAAACCGTTATTCATTCGTGATTGCGCCTGAGCGAGACGAAATACGCGATC
GCTGTTAAAAGGACAATTACAAACAGGAATCGAATGCAACCGGCGCAGGAACACTGCCAGCGCATCAACAATATTTTCACCTGAATCAGGATATTCTTCTAATACCTGGAATG
CTGTTTTCCCGGGGATCGCAGTGGTGAGTAACCATGCATCATCAGGAGTACGGATAAAATGCTTGATGGTCGGAAGAGGCATAAATTCCGTCAGCCAGTTTAGTCTGACCAT
CTCATCTGTAACATCATTGGCAACGCTACCTTTGCCATGTTTCAGAAACAACTCTGGCGCATCGGGCTTCCCATACAATCGATAGATTGTCGCACCTGATTGCCCGACATTAT
CGCGAGCCCATTTATACCCATATAAATCAGCATCCATGTTGGAATTTAATCGCGGCCTCGAGCAAGACGTTTCCCGTTGAATATGGCTCATAACACCCCTTGTATTACTGTTTA
TGTAAGCAGACAGTTTTATTGTTCATGATGATATATTTTTATCTTGTGCAATGTAACATCAGAGATTTTGAGACACAACGTGGCTTTCCCCCCCCCCCCCTGCAGGTCTCGGG
CTATTCTTTTGATTTATAAGGGATTTTGCCGATTTCGGCCTATTGGTTAAAAAATGAGCTGATTTAACAAAAATTTAACGCGAATTTTAACAAAATATTAACGTTTACAATTTAAA
TATTTGCTTATACAATCTTCCTGTTTTTGGGGCTTTTCTGATTATCAACCGGGGTACATATGATTGACATGCTAGTTTTACGATTACCGTTCATCGATTCTCTTGTTTGCTCCAG
ACTCTCAGGCAATGACCTGATAGCCTTTGTAGACCTCTCAAAAATAGCTACCCTCTCCGGCATGAATTTATCAGCTAGAACGGTTGAATATCATATTGATGGTGATTTGACTGT
CTCCGGCCTTTCTCACCCTTTTGAATCTTTACCTACACATTACTCAGGCATTGCATTTAAAATATATGAGGGTTCTAAAAATTTTTATCCTTGCGTTGAAATAAAGGCTTCTCCC
GCAAAAGTATTACAGGGTCATAATGTTTTTGGTACAACCGATTTAGCTTTATGCTCTGAGGCTTTATTGCTTAATTTTGCTAATTCTTTGCCTTGCCTGTATGATTTATTGGATG
TT

Scaffold sequence for Long staples with High staple crossover density and High scaffold crossover density (LHH) (in red = 1082bp) in M13K07 (in black=7760bp):

AACGCTACTACTATTAGTAGAATTGATGCCACCTTTTCAGCTCGCGCCCCAAATGAAAATATAGCTAAACAGGTTATTGACCATTTGCGAAATGTATCTAATGGTCAAACTAAA
TCTACTCGTTCGCAGAATTGGGAATCAACTGTTACATGGAATGAAACTTCCAGACACCGTACTTTAGTTGCATATTTAAAACATGTTGAGCTACAGCACCAGATTCAGCAATTA
AGCTCTAAGCCATCCGCAAAAATGACCTCTTATCAAAAGGAGCAATTAAAGGTACTCTCTAATCCTGACCTGTTGGAGTTTGCTTCCGGTCTGGTTCGCTTTGAAGCTCGAAT
TAAAACGCGATATTTGAAGTCTTTCGGGCTTCCTCTTAATCTTTTTGATGCAATCCGCTTTGCTTCTGACTATAATAGTCAGGGTAAAGACCTGATTTTTGATTTATGGTCATTC
TCGTTTTCTGAACTGTTTAAAGCATTTGAGGGGGATTCAATGAATATTTATGACGATTCCGCAGTATTGGACGCTATCCAGTCTAAACATTTTACTATTACCCCCTCTGGCAAA
ACTTCTTTTGCAAAAGCCTCTCGCTATTTTGGTTTTTATCGTCGTCTGGTAAACGAGGGTTATGATAGTGTTGCTCTTACTATGCCTCGTAATTCCTTTTGGCGTTATGTATCTG
CATTAGTTGAATGTGGTATTCCTAAATCTCAACTGATGAATCTTTCTACCTGTAATAATGTTGTTCCGTTAGTTCGTTTTATTAACGTAGATTTTTCTTCCCAACGTCCTGACTGG
TATAATGAGCCAGTTCTTAAAATCGCATAAGGTAATTCACAATGATTAAAGTTGAAATTAAACCATCTCAAGCCCAATTTACTACTCGTTCTGGTGTTTCTCGTCAGGGCAAGC
CTTATTCACTGAATGAGCAGCTTTGTTACGTTGATTTGGGTAATGAATATCCGGTTCTTGTCAAGATTACTCTTGATGAAGGTCAGCCAGCCTATGCGCCTGGTCTGTACACC
GTTCATCTGTCCTCTTTCAAAGTTGGTCAGTTCGGTTCCCTTATGATTGACCGTCTGCGCCTCGTTCCGGCTAAGTAACATGGAGCAGGTCGCGGATTTCGACACAATTTATC
AGGCGATGATACAAATCTCCGTTGTACTTTGTTTCGCGCTTGGTATAATCGCTGGGGGTCAAAGATGAGTGTTTTAGTGTATTCTTTCGCCTCTTTCGTTTTAGGTTGGTGCCT
TCGTAGTGGCATTACGTATTTTACCCGTTTAATGGAAACTTCCTCATGAAAAAGTCTTTAGTCCTCAAAGCCTCTGTAGCCGTTGCTACCCTCGTTCCGATGCTGTCTTTCGCT
GCTGAGGGTGACGATCCCGCAAAAGCGGCCTTTAACTCCCTGCAAGCCTCAGCGACCGAATATATCGGTTATGCGTGGGCGATGGTTGTTGTCATTGTCGGCGCAACTATC
GGTATCAAGCTGTTTAAGAAATTCACCTCGAAAGCAAGCTGATAAACCGATACAATTAAAGGCTCCTTTTGGAGCCTTTTTTTTTGGAGATTTTCAACGTGAAAAAATTATTATT
CGCAATTCCTTTAGTTGTTCCTTTCTATTCTCACTCCGCTGAAACTGTTGAAAGTTGTTTAGCAAAACCCCATACAGAAAATTCATTTACTAACGTCTGGAAAGACGACAAAACT
TTAGATCGTTACGCTAACTATGAGGGTTGTCTGTGGAATGCTACAGGCGTTGTAGTTTGTACTGGTGACGAAACTCAGTGTTACGGTACATGGGTTCCTATTGGGCTTGCTAT
CCCTGAAAATGAGGGTGGTGGCTCTGAGGGTGGCGGTTCTGAGGGTGGCGGTTCTGAGGGTGGCGGTACTAAACCTCCTGAGTACGGTGATACACCTATTCCGGGCTATA
CTTATATCAACCCTCTCGACGGCACTTATCCGCCTGGTACTGAGCAAAACCCCGCTAATCCTAATCCTTCTCTTGAGGAGTCTCAGCCTCTTAATACTTTCATGTTTCAGAATA
ATAGGTTCCGAAATAGGCAGGGGGCATTAACTGTTTATACGGGCACTGTTACTCAAGGCACTGACCCCGTTAAAACTTATTACCAGTACACTCCTGTATCATCAAAAGCCATG
TATGACGCTTACTGGAACGGTAAATTCAGAGACTGCGCTTTCCATTCTGGCTTTAATGAGGATCCATTCGTTTGTGAATATCAAGGCCAATCGTCTGACCTGCCTCAACCTCC
TGTCAATGCTGGCGGCGGCTCTGGTGGTGGTTCTGGTGGCGGCTCTGAGGGTGGTGGCTCTGAGGGTGGCGGTTCTGAGGGTGGCGGCTCTGAGGGAGGCGGTTCCGG
TGGTGGCTCTGGTTCCGGTGATTTTGATTATGAAAAGATGGCAAACGCTAATAAGGGGGCTATGACCGAAAATGCCGATGAAAACGCGCTACAGTCTGACGCTAAAGGCAAA
CTTGATTCTGTCGCTACTGATTACGGTGCTGCTATCGATGGTTTCATTGGTGACGTTTCCGGCCTTGCTAATGGTAATGGTGCTACTGGTGATTTTGCTGGCTCTAATTCCCA
AATGGCTCAAGTCGGTGACGGTGATAATTCACCTTTAATGAATAATTTCCGTCAATATTTACCTTCCCTCCCTCAATCGGTTGAATGTCGCCCTTTTGTCTTTAGCGCTGGTAA
ACCATATGAATTTTCTATTGATTGTGACAAAATAAACTTATTCCGTGGTGTCTTTGCGTTTCTTTTATATGTTGCCACCTTTATGTATGTATTTTCTACGTTTGCTAACATACTGC
GTAATAAGGAGTCTTAATCATGCCAGTTCTTTTGGGTATTCCGTTATTATTGCGTTTCCTCGGTTTCCTTCTGGTAACTTTGTTCGGCTATCTGCTTACTTTTCTTAAAAAGGGC
TTCGGTAAGATAGCTATTGCTATTTCATTGTTTCTTGCTCTTATTATTGGGCTTAACTCAATTCTTGTGGGTTATCTCTCTGATATTAGCGCTCAATTACCCTCTGACTTTGTTCA
GGGTGTTCAGTTAATTCTCCCGTCTAATGCGCTTCCCTGTTTTTATGTTATTCTCTCTGTAAAGGCTGCTATTTTCATTTTTGACGTTAAACAAAAAATCGTTTCTTATTTGGATT
GGGATAAATAATATGGCTGTTTATTTTGTAACTGGCAAATTAGGCTCTGGAAAGACGCTCGTTAGCGTTGGTAAGATTCAGGATAAAATTGTAGCTGGGTGCAAAATAGCAAC
TAATCTTGATTTAAGGCTTCAAAACCTCCCGCAAGTCGGGAGGTTCGCTAAAACGCCTCGCGTTCTTAGAATACCGGATAAGCCTTCTATATCTGATTTGCTTGCTATTGGGC
GCGGTAATGATTCCTACGATGAAAATAAAAACGGCTTGCTTGTTCTCGATGAGTGCGGTACTTGGTTTAATACCCGTTCTTGGAATGATAAGGAAAGACAGCCGATTATTGAT
TGGTTTCTACATGCTCGTAAATTAGGATGGGATATTATTTTTCTTGTTCAGGACTTATCTATTGTTGATAAACAGGCGCGTTCTGCATTAGCTGAACATGTTGTTTATTGTCGTC
GTCTGGACAGAATTACTTTACCTTTTGTCGGTACTTTATATTCTCTTATTACTGGCTCGAAAATGCCTCTGCCTAAATTACATGTTGGCGTTGTTAAATATGGCGATTCTCAATT
AAGCCCTACTGTTGAGCGTTGGCTTTATACTGGTAAGAATTTGTATAACGCATATGATACTAAACAGGCTTTTTCTAGTAATTATGATTCCGGTGTTTATTCTTATTTAACGCCT
TATTTATCACACGGTCGGTATTTCAAACCATTAAATTTAGGTCAGAAGATGAAATTAACTAAAATATATTTGAAAAAGTTTTCTCGCGTTCTTTGTCTTGCGATTGGATTTGCATC
AGCATTTACATATAGTTATATAACCCAACCTAAGCCGGAGGTTAAAAAGGTAGTCTCTCAGACCTATGATTTTGATAAATTCACTATTGACTCTTCTCAGCGTCTTAATCTAAGC
TATCGCTATGTTTTCAAGGATTCTAAGGGAAAATTAATTAATAGCGACGATTTACAGAAGCAAGGTTATTCACTCACATATATTGATTTATGTACTGTTTCCATTAAAAAAGGTA
ATTCAAATGAAATTGTTAAATGTAATTAATTTTGTTTTCTTGATGTTTGTTTCATCATCTTCTTTTGCTCAGGTAATTGAAATGAATAATTCGCCTCTGCGCGATTTTGTAACTTGG
TATTCAAAGCAATCAGGCGAATCCGTTATTGTTTCTCCCGATGTAAAAGGTACTGTTACTGTATATTCATCTGACGTTAAACCTGAAAATCTACGCAATTTCTTTATTTCTGTTTT
ACGTGCTAATAATTTTGATATGGTTGGTTCAATTCCTTCCATAATTCAGAAGTATAATCCAAACAATCAGGATTATATTGATGAATTGCCATCATCTGATAATCAGGAATATGAT
GATAATTCCGCTCCTTCTGGTGGTTTCTTTGTTCCGCAAAATGATAATGTTACTCAAACTTTTAAAATTAATAACGTTCGGGCAAAGGATTTAATACGAGTTGTCGAATTGTTTG
TAAAGTCTAATACTTCTAAATCCTCAAATGTATTATCTATTGACGGCTCTAATCTATTAGTTGTTAGTGCACCTAAAGATATTTTAGATAACCTTCCTCAATTCCTTTCTACTGTT
GATTTGCCAACTGACCAGATATTGATTGAGGGTTTGATATTTGAGGTTCAGCAAGGTGATGCTTTAGATTTTTCATTTGCTGCTGGCTCTCAGCGTGGCACTGTTGCAGGCGG
TGTTAATACTGACCGCCTCACCTCTGTTTTATCTTCTGCTGGTGGTTCGTTCGGTATTTTTAATGGCGATGTTTTAGGGCTATCAGTTCGCGCATTAAAGACTAATAGCCATTC
AAAAATATTGTCTGTGCCACGTATTCTTACGCTTTCAGGTCAGAAGGGTTCTATCTCTGTTGGCCAGAATGTCCCTTTTATTACTGGTCGTGTGACTGGTGAATCTGCCAATGT
AAATAATCCATTTCAGACGATTGAGCGTCAAAATGTAGGTATTTCCATGAGCGTTTTTCCTGTTGCAATGGCTGGCGGTAATATTGTTCTGGATATTACCAGCAAGGCCGATA

GTTTGAGTTCTTCTACTCAGGCAAGTGATGTTATTACTAATCAAAGAAGTATTGCTACAACGGTTAATTTGCGTGATGGACAGACTCTTTTACTCGGTGGCCTCACTGATTATA
AAAACACTTCTCAAGATTCTGGCGTACCGTTCCTGTCTAAAATCCCTTTAATCGGCCTCCTGTTTAGCTCCCGCTCTGATTCCAACGAGGAAAGCACGTTATACGTGCTCGTC
AAAGCAACCATAGTACGCGCCCTGTAGCGGCGCATTAAGCGCGGCGGGTGTGGTGGTTACGCGCAGCGTGACCGCTACACTTGCCAGCGCCCTAGCGCCCGCTCCTTTC
GCTTTCTTCCCTTCCTTTCTCGCCACGTTCGCCGGCTTTCCCCGTCAAGCTCTAAATCGGGGGCTCCCTTTAGGGTTCCGATTTAGTGCTTTACGGCACCTCGACCCCAAAA
AACTTGATTTGGGTGATGGTTCACGTAGTGGGCCATCGCCCTGATAGACGGTTTTTCGCCCTTTGACGTTGGAGTCCACGTTCTTTAATAGTGGACTCTTGTTCCAAACTGGA
ACAACACTCAACCCTATCTCGGGACGGATCGCTTCATGTGGCAGGAGAAAAAGGCTGCACCGGTGCGTCAGCAGAATATGTGATACAGGATATATTCCGCTTCCTCGCTC
ACTGACTCGCTACGCTCGGTCGTTCGACTGCGGCGAGCGGAAATGGCTTACGAACGGGGCGGAGATTTCCTGGAAGATGCCAGGAAGATACTTAACAGGGAAGTGAGAGG
GCCGCGGAGGAATAGGGCGGCCAAGTGCTATACCGAGAAAAGTTACGCATTCTGGTTGCAGTGATTAACTAATCGCGAACACCTCCCCAGTCGTTACTAGGAGCGGCGTAC
GTATTGCTAGTCGAACGGGAGCCGTGGACAGCGGACCTCCTATAGTTGACTGTTATATTATCCAGCCTCGCTTAAACAAGTGCAATATCTCTAGGCTGCCTGCGATGAGTTT
GCCTGAAGACAGTATAGGATGGGGGAACTCTCTTAAACTTAGTTGTCTACCGACATAAAGGAAATCAATACGCCGCGTATACCGAGAGGCCACCGTACGCTTTGGACTTAAA
GTTATAACTAATCGCGAAGACTCACCTATAGATCGTTAGGTCCAGAATCATACTGAGTAGTCGAACGGGAGTACAAAACTGAGTACCTGTTCCGTGGATGTTATATTATCCAT
CGACACTTGCACAAATGCCTCAGTGGCTAGACCAGATTGCAAAGCCGCACCCAGATGGGATGTGAAGCACCGGATGGGTAGGGGAAGCAAGATGAGGGTGAAGGGTAGAA
CGGGTTGCAGAAATTACCTATTATCCATCGTTGGACAAACACATCTTCAAGTCTTGCGCCAAGTGCGGGCGATAGCAGTTGACCAGCGGACCTCTTTGTATTGCAGCAGAGC
GGCCTCTTAATTTTACACACCACCCCAGTCGTTACACCCCACCTCCCTGCTTATCTCCCCCAGTGATCGCATTCTGGCCAGACGACTATGTGCCCACAAGAGTTCTCGATCA
AGCGACCAACTAACGTCGATCCTAGGTGGTAGGCCCATCTTTCGACACCAGGACCGTCTGTTTACAAGTCCCGCCATGTTGGATACTGAGAGCAGTCCAGAATCACAAACTA
ATGGGGGCGATAGCAGATCGTACCCGACTCACTTTGCTTTGGACTTGGCCACCCTCTTAATTTTACAAATACACATCCATCGAAATCCACCACCCTGCTTATCTCCCCTCTAC
AACTGAGTACAGCCATCACAAACTTGGAGGAGGGAGGGTAGAACGGGTTGGGGTGGGAGCCTATTATCTTCAGGATGAGTGGGTCGACGAGCTCGCCCCGGGGATCGAC
CAGTTGGTGATTTTGAACTTTTGCTTTGCCACGGAACGGTCTGCGTTGTCGGGAAGATGCGTGATCTGATCCTTCAACTCAGCAAAAGTTCGATTTATTCAACAAAGCCGCCG
TCCCGTCAAGTCAGCGTAATGCTCTGCCAGTGTTACAACCAATTAACCAATTCTGATTAGAAAAACTCATCGAGCATCAAATGAAACTGCAATTTATTCATATCAGGATTATCA
ATACCATATTTTTGAAAAAGCCGTTTCTGTAATGAAGGAGAAAACTCACCGAGGCAGTTCCATAGGATGGCAAGATCCTGGTATCGGTCTGCGATTCCGACTCGTCCAACATC
AATACAACCTATTAATTTCCCCTCGTCAAAAATAAGGTTATCAAGTGAGAAATCACCATGAGTGACGACTGAATCCGGTGAGAATGGCAAAAGCTTATGCATTTCTTTCCAGAC
TTGTTCAACAGGCCAGCCATTACGCTCGTCATCAAAATCACTCGCATCAACCAAACCGTTATTCATTCGTGATTGCGCCTGAGCGAGACGAAATACGCGATCGCTGTTAAAA
GGACAATTACAAACAGGAATCGAATGCAACCGGCGCAGGAACACTGCCAGCGCATCAACAATATTTTCACCTGAATCAGGATATTCTTCTAATACCTGGAATGCTGTTTTCCC
GGGGATCGCAGTGGTGAGTAACCATGCATCATCAGGAGTACGGATAAAATGCTTGATGGTCGGAAGAGGCATAAATTCCGTCAGCCAGTTTAGTCTGACCATCTCATCTGTA
ACATCATTGGCAACGCTACCTTTGCCATGTTTCAGAAACAACTCTGGCGCATCGGGCTTCCCATACAATCGATAGATTGTCGCACCTGATTGCCCGACATTATCGCGAGCCC
ATTTATACCCATATAAATCAGCATCCATGTTGGAATTTAATCGCGGCCTCGAGCAAGACGTTTCCCGTTGAATATGGCTCATAACACCCCTTGTATTACTGTTTATGTAAGCAG
ACAGTTTTATTGTTCATGATGATATATTTTTATCTTGTGCAATGTAACATCAGAGATTTTGAGACACAACGTGGCTTTCCCCCCCCCCCCCTGCAGGTCTCGGGCTATTCTTTT
GATTTATAAGGGATTTTGCCGATTTCGGCCTATTGGTTAAAAAATGAGCTGATTTAACAAAAATTTAACGCGAATTTTAACAAAATATTAACGTTTACAATTTAAATATTTGCTTA
TACAATCTTCCTGTTTTTGGGGCTTTTCTGATTATCAACCGGGGTACATATGATTGACATGCTAGTTTTACGATTACCGTTCATCGATTCTCTTGTTTGCTCCAGACTCTCAGG
CAATGACCTGATAGCCTTTGTAGACCTCTCAAAAATAGCTACCCTCTCCGGCATGAATTTATCAGCTAGAACGGTTGAATATCATATTGATGGTGATTTGACTGTCTCCGGCCT
TTCTCACCCTTTTGAATCTTTACCTACACATTACTCAGGCATTGCATTTAAAATATATGAGGGTTCTAAAAATTTTTATCCTTGCGTTGAAATAAAGGCTTCTCCCGCAAAAGTAT
TACAGGGTCATAATGTTTTTGGTACAACCGATTTAGCTTTATGCTCTGAGGCTTTATTGCTTAATTTTGCTAATTCTTTGCCTTGCCTGTATGATTTATTGGATGTT

Scaffold sequence for folding with 10 different staple sequences with three ~2kb DNA blocks from Genewiz where block 01 is shown in orange, block 02 in blue and block 03 in green were Gibson cloned into M13K07 RF (black):

AACGCTACTACTATTAGTAGAATTGATGCCACCTTTTCAGCTCGCGCCCCAAATGAAAATATAGCTAAACAGGTTATTGACCATTTGCGAAATGTATCTAATGGTCAAACTAAA
TCTACTCGTTCGCAGAATTGGGAATCAACTGTTACATGGAATGAAACTTCCAGACACCGTACTTTAGTTGCATATTTAAAACATGTTGAGCTACAGCACCAGATTCAGCAATTA
AGCTCTAAGCCATCCGCAAAAATGACCTCTTATCAAAAGGAGCAATTAAAGGTACTCTCTAATCCTGACCTGTTGGAGTTTGCTTCCGGTCTGGTTCGCTTTGAAGCTCGAAT
TAAAACGCGATATTTGAAGTCTTTCGGGCTTCCTCTTAATCTTTTTGATGCAATCCGCTTTGCTTCTGACTATAATAGTCAGGGTAAAGACCTGATTTTTGATTTATGGTCATTC
TCGTTTTCTGAACTGTTTAAAGCATTTGAGGGGGATTCAATGAATATTTATGACGATTCCGCAGTATTGGACGCTATCCAGTCTAAACATTTTACTATTACCCCCTCTGGCAAA
ACTTCTTTTGCAAAAGCCTCTCGCTATTTTGGTTTTTATCGTCGTCTGGTAAACGAGGGTTATGATAGTGTTGCTCTTACTATGCCTCGTAATTCCTTTTGGCGTTATGTATCTG
CATTAGTTGAATGTGGTATTCCTAAATCTCAACTGATGAATCTTTCTACCTGTAATAATGTTGTTCCGTTAGTTCGTTTTATTAACGTAGATTTTTCTTCCCAACGTCCTGACTGG
TATAATGAGCCAGTTCTTAAAATCGCATAAGGTAATTCACAATGATTAAAGTTGAAATTAAACCATCTCAAGCCCAATTTACTACTCGTTCTGGTGTTTCTCGTCAGGGCAAGC
CTTATTCACTGAATGAGCAGCTTTGTTACGTTGATTTGGGTAATGAATATCCGGTTCTTGTCAAGATTACTCTTGATGAAGGTCAGCCAGCCTATGCGCCTGGTCTGTACACC
GTTCATCTGTCCTCTTTCAAAGTTGGTCAGTTCGGTTCCCTTATGATTGACCGTCTGCGCCTCGTTCCGGCTAAGTAACATGGAGCAGGTCGCGGATTTCGACACAATTTATC
AGGCGATGATACAAATCTCCGTTGTACTTTGTTTCGCGCTTGGTATAATCGCTGGGGGTCAAAGATGAGTGTTTTAGTGTATTCTTTCGCCTCTTTCGTTTTAGGTTGGTGCCT
TCGTAGTGGCATTACGTATTTTACCCGTTTAATGGAAACTTCCTCATGAAAAAGTCTTTAGTCCTCAAAGCCTCTGTAGCCGTTGCTACCCTCGTTCCGATGCTGTCTTTCGCT
GCTGAGGGTGACGATCCCGCAAAAGCGGCCTTTAACTCCCTGCAAGCCTCAGCGACCGAATATATCGGTTATGCGTGGGCGATGGTTGTTGTCATTGTCGGCGCAACTATC
GGTATCAAGCTGTTTAAGAAATTCACCTCGAAAGCAAGCTGATAAACCGATACAATTAAAGGCTCCTTTTGGAGCCTTTTTTTTTGGAGATTTTCAACGTGAAAAAATTATTATT
CGCAATTCCTTTAGTTGTTCCTTTCTATTCTCACTCCGCTGAAACTGTTGAAAGTTGTTTAGCAAAACCCCATACAGAAAATTCATTTACTAACGTCTGGAAAGACGACAAAACT
TTAGATCGTTACGCTAACTATGAGGGTTGTCTGTGGAATGCTACAGGCGTTGTAGTTTGTACTGGTGACGAAACTCAGTGTTACGGTACATGGGTTCCTATTGGGCTTGCTAT
CCCTGAAAATGAGGGTGGTGGCTCTGAGGGTGGCGGTTCTGAGGGTGGCGGTTCTGAGGGTGGCGGTACTAAACCTCCTGAGTACGGTGATACACCTATTCCGGGCTATA
CTTATATCAACCCTCTCGACGGCACTTATCCGCCTGGTACTGAGCAAAACCCCGCTAATCCTAATCCTTCTCTTGAGGAGTCTCAGCCTCTTAATACTTTCATGTTTCAGAATA
ATAGGTTCCGAAATAGGCAGGGGGCATTAACTGTTTATACGGGCACTGTTACTCAAGGCACTGACCCCGTTAAAACTTATTACCAGTACACTCCTGTATCATCAAAAGCCATG
TATGACGCTTACTGGAACGGTAAATTCAGAGACTGCGCTTTCCATTCTGGCTTTAATGAGGATCCATTCGTTTGTGAATATCAAGGCCAATCGTCTGACCTGCCTCAACCTCC
TGTCAATGCTGGCGGCGGCTCTGGTGGTGGTTCTGGTGGCGGCTCTGAGGGTGGTGGCTCTGAGGGTGGCGGTTCTGAGGGTGGCGGCTCTGAGGGAGGCGGTTCCGG
TGGTGGCTCTGGTTCCGGTGATTTTGATTATGAAAAGATGGCAAACGCTAATAAGGGGGCTATGACCGAAAATGCCGATGAAAACGCGCTACAGTCTGACGCTAAAGGCAAA
CTTGATTCTGTCGCTACTGATTACGGTGCTGCTATCGATGGTTTCATTGGTGACGTTTCCGGCCTTGCTAATGGTAATGGTGCTACTGGTGATTTTGCTGGCTCTAATTCCCA
AATGGCTCAAGTCGGTGACGGTGATAATTCACCTTTAATGAATAATTTCCGTCAATATTTACCTTCCCTCCCTCAATCGGTTGAATGTCGCCCTTTTGTCTTTAGCGCTGGTAA
ACCATATGAATTTTCTATTGATTGTGACAAAATAAACTTATTCCGTGGTGTCTTTGCGTTTCTTTTATATGTTGCCACCTTTATGTATGTATTTTCTACGTTTGCTAACATACTGC
GTAATAAGGAGTCTTAATCATGCCAGTTCTTTTGGGTATTCCGTTATTATTGCGTTTCCTCGGTTTCCTTCTGGTAACTTTGTTCGGCTATCTGCTTACTTTTCTTAAAAAGGGC
TTCGGTAAGATAGCTATTGCTATTTCATTGTTTCTTGCTCTTATTATTGGGCTTAACTCAATTCTTGTGGGTTATCTCTCTGATATTAGCGCTCAATTACCCTCTGACTTTGTTCA
GGGTGTTCAGTTAATTCTCCCGTCTAATGCGCTTCCCTGTTTTTATGTTATTCTCTCTGTAAAGGCTGCTATTTTCATTTTTGACGTTAAACAAAAAATCGTTTCTTATTTGGATT
GGGATAAATAATATGGCTGTTTATTTTGTAACTGGCAAATTAGGCTCTGGAAAGACGCTCGTTAGCGTTGGTAAGATTCAGGATAAAATTGTAGCTGGGTGCAAAATAGCAAC
TAATCTTGATTTAAGGCTTCAAAACCTCCCGCAAGTCGGGAGGTTCGCTAAAACGCCTCGCGTTCTTAGAATACCGGATAAGCCTTCTATATCTGATTTGCTTGCTATTGGGC
GCGGTAATGATTCCTACGATGAAAATAAAAACGGCTTGCTTGTTCTCGATGAGTGCGGTACTTGGTTTAATACCCGTTCTTGGAATGATAAGGAAAGACAGCCGATTATTGAT
TGGTTTCTACATGCTCGTAAATTAGGATGGGATATTATTTTTCTTGTTCAGGACTTATCTATTGTTGATAAACAGGCGCGTTCTGCATTAGCTGAACATGTTGTTTATTGTCGTC
GTCTGGACAGAATTACTTTACCTTTTGTCGGTACTTTATATTCTCTTATTACTGGCTCGAAAATGCCTCTGCCTAAATTACATGTTGGCGTTGTTAAATATGGCGATTCTCAATT
AAGCCCTACTGTTGAGCGTTGGCTTTATACTGGTAAGAATTTGTATAACGCATATGATACTAAACAGGCTTTTTCTAGTAATTATGATTCCGGTGTTTATTCTTATTTAACGCCT
TATTTATCACACGGTCGGTATTTCAAACCATTAAATTTAGGTCAGAAGATGAAATTAACTAAAATATATTTGAAAAAGTTTTCTCGCGTTCTTTGTCTTGCGATTGGATTTGCATC
AGCATTTACATATAGTTATATAACCCAACCTAAGCCGGAGGTTAAAAAGGTAGTCTCTCAGACCTATGATTTTGATAAATTCACTATTGACTCTTCTCAGCGTCTTAATCTAAGC
TATCGCTATGTTTTCAAGGATTCTAAGGGAAAATTAATTAATAGCGACGATTTACAGAAGCAAGGTTATTCACTCACATATATTGATTTATGTACTGTTTCCATTAAAAAAGGTA
ATTCAAATGAAATTGTTAAATGTAATTAATTTTGTTTTCTTGATGTTTGTTTCATCATCTTCTTTTGCTCAGGTAATTGAAATGAATAATTCGCCTCTGCGCGATTTTGTAACTTGG
TATTCAAAGCAATCAGGCGAATCCGTTATTGTTTCTCCCGATGTAAAAGGTACTGTTACTGTATATTCATCTGACGTTAAACCTGAAAATCTACGCAATTTCTTTATTTCTGTTTT

ACGTGCTAATAATTTTGATATGGTTGGTTCAATTCCTTCCATAATTCAGAAGTATAATCCAAACAATCAGGATTATATTGATGAATTGCCATCATCTGATAATCAGGAATATGAT
GATAATTCCGCTCCTTCTGGTGGTTTCTTTGTTCCGCAAAATGATAATGTTACTCAAACTTTTAAAATTAATAACGTTCGGGCAAAGGATTTAATACGAGTTGTCGAATTGTTTG
TAAAGTCTAATACTTCTAAATCCTCAAATGTATTATCTATTGACGGCTCTAATCTATTAGTTGTTAGTGCACCTAAAGATATTTTAGATAACCTTCCTCAATTCCTTTCTACTGTT
GATTTGCCAACTGACCAGATATTGATTGAGGGTTTGATATTTGAGGTTCAGCAAGGTGATGCTTTAGATTTTTCATTTGCTGCTGGCTCTCAGCGTGGCACTGTTGCAGGCGG
TGTTAATACTGACCGCCTCACCTCTGTTTTATCTTCTGCTGGTGGTTCGTTCGGTATTTTTAATGGCGATGTTTTAGGGCTATCAGTTCGCGCATTAAAGACTAATAGCCATTC
AAAAATATTGTCTGTGCCACGTATTCTTACGCTTTCAGGTCAGAAGGGTTCTATCTCTGTTGGCCAGAATGTCCCTTTTATTACTGGTCGTGTGACTGGTGAATCTGCCAATGT
AAATAATCCATTTCAGACGATTGAGCGTCAAAATGTAGGTATTTCCATGAGCGTTTTTCCTGTTGCAATGGCTGGCGGTAATATTGTTCTGGATATTACCAGCAAGGCCGATA
GTTTGAGTTCTTCTACTCAGGCAAGTGATGTTATTACTAATCAAAGAAGTATTGCTACAACGGTTAATTTGCGTGATGGACAGACTCTTTTACTCGGTGGCCTCACTGATTATA
AAAACACTTCTCAAGATTCTGGCGTACCGTTCCTGTCTAAAATCCCTTTAATCGGCCTCCTGTTTAGCTCCCGCTCTGATTCCAACGAGGAAAGCACGTTATACGTGCTCGTC
AAAGCAACCATAGTACGCGCCCTGTAGCGGCGCATTAAGCGCGGCGGGTGTGGTGGTTACGCGCAGCGTGACCGCTACACTTGCCAGCGCCCTAGCGCCCGCTCCTTTC
GCTTTCTTCCCTTCCTTTCTCGCCACGTTCGCCGGCTTTCCCCGTCAAGCTCTAAATCGGGGGCTCCCTTTAGGGTTCCGATTTAGTGCTTTACGGCACCTCGACCCCAAAA
AACTTGATTTGGGTGATGGTTCACGTAGTGGGCCATCGCCCTGATAGACGGTTTTTCGCCCTTTGACGTTGGAGTCCACGTTCTTTAATAGTGGACTCTTGTTCCAAACTGGA
ACAACACTCAACCCTATCTCGGGACGGATCGCTTCATGTGGCAGGAGAAAAAAGGCTGCACCGGTGCGTCAGCAGAATATGTGATACAGGATATATTCCGCTTCCTCGCTC
ACTGACTCGCTACGCTCGGTCGTTCGACTGCGGCGAGCGGAAATGGCTTACGAACGGGGCGGAGATTTCCTGGAAGATGCCAGGAAGATACTTAACAGGGAAGTGAGAGG
GCCGCGGCATCCGCGCGAAGCGCGGATGCCGCGAAGGCCTCCAAAGCGAATGCACGTAGTCCCAGTTTGACTAACAGGACGCCCCTCGCTGTGCTCCTTATGGAAGCGA
GAATGCCTCCAAGCATATGATCATCAGGAGTATGGATCAGGGTACGTATCTGTATATCTACACTGTTGCCGTTTTGTGCAGACCGTAGCGAATTCGCACGGATGGTTCGCTT
CGCCTAAATGCGCTCCCAATACGCCTACGTGTGCGCGAACAGCGTCGCACGGAGAATGAGACCGTTTGAGCATCACTGGAAGCAAGTCCTCACAGTTCGGATGGAAACATC
GATGGTTGCACGTACTTAACTACACTGAAAACATCAAGTCCTCACAGTTGCGAAGGAGAAGATATTTGTCCCTGCAATGGCCAGCTGCGTCTATGGGCATACGCGCTCCTAT
TATGCCTGCGAGCGAACTGACCGTCCCTACGTGAGGCTACCGATAAAGCGAATTGGAAGCGCGAAGGAATAGGGGCGAACTCGGAGACGATGGTTATATGAGAATTGCGA
ACTAAACATCTAGACGTAAAACTCCCGTTTCAGACCGTGCATATGCCTCCAAATCCAGTACACGCGAACAGCGCTTTCGATCGGCCGGTTGAGGGTCCCAGTTTGACTGGAT
CAGGGTACGTATCTGTATATCTACACTGAACAGGACAGTATGGCCGGTTGTCGGCGCGGATGGGTGCGCGGCTCTCTTGGAAGCGAGAATGATCATCACGCTTCGCAACAA
CATGTCGGAACAGCGCCTACGTCCCTACACTGAGTGCGCGCCGATAAATCCAGTCTCCTTAGGAGTATTTGCCGTGATGGTTGACGGGATTTGTGCAATAGGGTGGATTGC
TTGTATGTGCGCGCAATACGGGTATATCCTGCAATGGCCAGCTGCGTCTATGGGCATACGACCGTCCGCTGTGCGGATGGGACGATGCTTAACTCCGTTTCAGAAGATGTG
CCCTAAACATCGGATTGTCCTCCAACGCTGTGACACGCGCTTAACTCGGATGGGAGGCTAGACGATTATGTCGCACGTTTGTAGCCTAAATGCTCTCTCCTGCGAGAGAATG
CAGTATGATGTCGGGACGATGATTTGTCGGTATATCCTAAATGTGCCCTATATGAGCGCCCCTCTTTCGAGGATCAGGGTACGTATCTGTATATCTACACTGTCGGCCGCAG
TATGGCATCACATTTCGTAACAGCGCAATACGGTCATGACCGTTTCGCACGTAGCCGGTTGACCGTCGTTGAGGGACGGGAAAACATCGACTCCAAAGTCCTATCATATATG
CACAGTTTATTATGCCTACGTTTTGTGCAACAGCGGGTATATGTTGAGGGCGAACTCAACAACCTTATGGAAAACTCGCACGTAGACTCCAGACGGGAGGATTGTGTCGGCG
CCTGCAATGGCCAGCTGCGTCTATGGGCATACTGGATTGCTTGTATGCTCTCTTTTGTAGCGGAGACTAGACGTTCTTCATAGCAGTTCACAGTTCCTGCGAGCGAAGGTAT
TATGAGAAGATATTTGTCATATGAGGCGAACTCCCTGTTTCGAGCATCACGCGCTCCGCATCACATATGAGAGACCGTTCGCACGAAGTCCTAACACCGTTTGTAGCGGAGA
CTAGACGTATCCAGTACACGCGGCGCTCCGGATCAGGGTACGTATCTGTATATCTACACTGCGCCCCTTTCAGCACCGATAAAAAACTCTTTGACTGACTCCATTCAGCACC
TCCAAATTTGTCGCATATGCAGTATGGTTTCGACGCCCCTGTGCCCTTTCAGCAACACTGACGGTGGAGTATAACACCGATTTCGTGATGGTTTTCAGCAGCCGGTTCTTTCG
AAGAAGATAGCGAATAATAGGGATTTCGTAACAGGACCTACGTATGTCGGGTTTCGAGCCGGTTCCTGCAATGGCCAGCTGCGTCTATGGGCATACGTTGAGGCCGTTTCG
GATTGTAAACATCCGCTTCGGTGCCCTCGGATGGAACAGCGTTTGTGCTCGGCCGGCGCTCCAACACCGTGGAAGCCCGTTTCAGCAGTTTTGCCGTCAATGTGCCCTTCT
TCATAATAGGGACACTGAAGCAGTTCGCCCCTAGCGAATGACGGGACCTACGTATTTCGTGACGATGCAACAACACACTGATTGCCGTAATAGGGCTCCTTAGGATCAGGG
TACGTATCTGTATATCTACACTGCGCTTCGATCATCAGGAGTATGAGGCTAAGCGAATGCACGTAGACTCCAACACGCGGCTCTCTGCACGTACTTATGGTCTTCATGAGGC
TAGTCATGACTTATGGATCATTTGTAGGCACGTAATGTCGGCCGATAAGTCATGAGCGCTCCCGCCCCTATCCAGTTTTGTAGCGGAGACCCTCCAAATGTCGGCCGATAAG
CATATGCAACAACCCGTTTCCCTGCAATGGCCAGCTGCGTCTATGGGCATACAATAGGGGCGAAGGAGACCGTATCCAGTTGGAAGCGAGAATGTAGACGTATGTCGGCCG
ATAACGCTGTGTGGATTGCTTGTATATCCAGTCCTCCAAAGCGAATCGGAGACTCGCAATTGTTAGACGTCAACAACGAGAATGACACGCGGGATTGTGTCGGCGCTCCTTA
TATTATGGTGCGCGAAAACTCCTCCTTAGATGGTTACACGCGCAACAACCCTGCGACGGATGGGGATCAGGGTACGTATCTGTATATCTACACTGGTGCGCGCTCCTTACG
CTTCGGCTCTCTCGCCCCTCGCTGTGAACAGCGGCATCACATATGAGCGCTTCGGGAGTATTTGCCGTGCTCTCTCTCCTTACGCTTCGAAAACTCGTCCCGGCTAGACGAT
GGACGATGCGCTGTGGACGGGAGTGCGCGATCATCACAACAACGAGAATGGTTTCGACCTACGTGTCCCAGGCGCTCCGACGGGAAACACCGCGCTGTGGTCATGACCT
GCAATGGCCAGCTGCGTCTATGGGCATACCTTATGGTCTTCATAGCAGTTTTTGTGCAGAAGATATTTGTCAAACATCGATGGTTTGGAAGCCCGTTTCAATAGGGCGGATG
GTTTGTGCAGAAGATATTTGTCGTGCCCTATTTCGTGACTCCAGTTTCGAGCATCACATATGAGACACGCGGCTCTCTGACGGGAGTCGGCGTGGATTGCTTGTATGCATAT
GATCATCAGGAGTATGGATCAGGGTACGTATCTGTATATCTACACTGTTGCCGTTCGCACGAGACCGTGTCCCAGGGAGTATTCGGCCGTTTGTGCATTTGTCATCCAGTTA
TTATGATCATCATTCAGCATTTGTAGGAAGTTTGACTAACACCGGGAGTATTTGCCGTCCGATAAAACAGCGCCTACGTCTTTCGATTGCCGTGGATTGTGCGAAGGAAGTCC
TCACAGTTCCTGCAATGGCCAGCTGCGTCTATGGGCATACGCGAACTCGGAGACCCTGCGAGCGCTCCGTCATGAGACCGTCAAACATCCGCTTCGATGTCGGGGATTGT
GTCGGCGAGCAGTTAACAGGAGGCGAAGTCCTTCTTCATTGGATTGCTTGTATAAAACTCCAACAACCCGTTTCGGTATATCTTATGGTCTTCATGTTGAGGTAGACGTAAAA
CTCGGATCAGGGTACGTATCTGTATATCTACACTGAACAGCGCAGTATGGCCGGTTAGCAGTTAACAGGACAATACGAATAGGGCGGAGACTAGACGTGTCCCAGGTCATG
ACTTATGGCACAGTTGACTCTTCGTGCGAACTCCTAAATAAAACTCTTTGACTAACAGGATTTGTAGCGGATGGGTGCGCGTTTGACTAACACCGTTCAGCATATTATGCCTA
CGTAAACATCCCTGCAATGGCCAGCTGCGTCTATGGGCATACGATGGTTCTTAACTATATGAGGTTTCGACGCTGTGGTCCCAGGTCCCAGTGGAAGCGACTCCACAATAC
GCTTATGGTAGACGTCTTAACTGGTATATACGACACTGATCGGCCGGTCATGAGGTATATCTCCTTAGATGGTTTTTGACTATTTGTCCGCCCCTGCATCACTTTGTAGCCTA
AATTGGAAGCGGATCAGGGTACGTATCTGTATATCTACACTGGAGAATGATGTCGGGACTCCAATATGAGAACAGGAAGAAGATAACAGGAATTTGTCCGCTGTGGACCGTC
GCCGGTTCTTTCGAGAGGCTATAGACGAGACGTCATGAGCATATGCAGTATGCTTTCGAAGAAGATAGAAGATCAATACGATCATCATTTGACTTATTATGGAGGCTAGGATT
GTCCTCCAACCTGCAATGGCCAGCTGCGTCTATGGGCATACAGCGAATGCACGTAACACTGAGTCATGACTTATGGGCATATGGGTATATGACCGTCTTTGTGCTCGGCCG
GTCGGCGAGACCGTCTTATGGTCTTCATCTTAATAAATAGCAGTTCCTGCGAGCGAAGGTGGATTGGCTCTCTGACGGGACTTTCGAGCGAAGGAGACCGTTCTTCATCCTA
AATCTTAACTGTCATGAGGATCAGGGTACGTATCTGTATATCTACACTGCTTATGGGTGCCCTGCGAACTAGCAGTTGACGGGACCTGCGATCGGCCGGCGCTCCACACGC
GCTTGTATGGTATATGTTGAGGAACACCGGCCAAAAACTCGCCGGTTGACCGTCGTTGAGGATCCAGTCGCCCCTTCGCACGGTTGAGGTTTGTGCCAATACGCACAGTTA
TTTCGTCCGATAACCTGCAATGGCCAGCTGCGTCTATGGGCATACATCCAGTGTTTCGAAAGTCCTGGTATATATATGAGGCCGGTTAAGTCCTGTGCCCTCCGATAACCTG
CGATTTGTAGCAGTATGTCGCACGTATGGACCGTCGGATTGTCGGAGACTCTTCATATGTCGGATTTCGTGAGAATGCAGTATGTATTATGCTCCTTATCGCACGGCATCACA
CACTGAGGATCAGGGTACGTATCTGTATATCTACACTGGAGGCTAAGAAGATATTTGTCCGCTTCGTGGATTGCTTGTATCCTCCAAACACTGAGAGGCTAAGCAGTTGGAG
TATTTGCCGTCTTTCGACTTAATAAATTCGGCCGGTCGGCGATCATCAAACACCGTTCAGCACTTAACTAGCGAATGCACGTAGTCGGCGCAACAACCCGTTTCTTTGTGCAA
CAGCGCCTACGTCCTGCAATGGCCAGCTGCGTCTATGGGCATACAAACATCGCTCTCTGACGGGAAATAGGGCTTTCGATCGGCCGGACGATGGCGAACTCCTAAATGTTT
CGAAGACCGTGCATATGTGGATTGCACAGGTCCTCTTGTATGACTCCAGCGAAGGGCATCACGACGATGCACAGTTGTGCCCTGCATCACGACCGTCCGGATGGTTCAGCA
ACACGCGGATGGTTGGATCAGGGTACGTATCTGTATATCTACACTGTGGAAGCAGCAGTTAACACCGGTGCGCGTTCAGCAGTTTCGAATTTCGTTGGATTGCTTGTATATAT
GAGGTGCCCTATTTCGTCAGTATGGTTTCCAGCAGCCGGTTCAATACGGACGATGTATTATGAAAACTCGTCGGCGGACTCCAGCTCTCTAACACCGATGTCGGGACGATGT
CTTCATGAGAATGCCTGCAATGGCCAGCTGCGTCTATGGGCATACCCTCCAAAGCGAATGCACGTACCGATAAATCCAGTACACGCGTTTGTAGCGGAGACTAGACGTGTC
CCAGTTTGACTAACAGGAGTTGAGGCCTGCGATCCATCCGCGCGAAGCGCGGATGGAGCTCGCCCCGGGGATCGACCAGTTGGTGATTTTGAACTTTTGCTTTGCCACGG
AACGGTCTGCGTTGTCGGGAAGATGCGTGATCTGATCCTTCAACTCAGCAAAAGTTCGATTTATTCAACAAAGCCGCCGTCCCGTCAAGTCAGCGTAATGCTCTGCCAGTGT
TACAACCAATTAACCAATTCTGATTAGAAAAACTCATCGAGCATCAAATGAAACTGCAATTTATTCATATCAGGATTATCAATACCATATTTTTGAAAAAGCCGTTTCTGTAATGA
AGGAGAAAACTCACCGAGGCAGTTCCATAGGATGGCAAGATCCTGGTATCGGTCTGCGATTCCGACTCGTCCAACATCAATACAACCTATTAATTTCCCCTCGTCAAAAATAA
GGTTATCAAGTGAGAAATCACCATGAGTGACGACTGAATCCGGTGAGAATGGCAAAAGCTTATGCATTTCTTTCCAGACTTGTTCAACAGGCCAGCCATTACGCTCGTCATCA
AAATCACTCGCATCAACCAAACCGTTATTCATTCGTGATTGCGCCTGAGCGAGACGAAATACGCGATCGCTGTTAAAAGGACAATTACAAACAGGAATCGAATGCAACCGGC
GCAGGAACACTGCCAGCGCATCAACAATATTTTCACCTGAATCAGGATATTCTTCTAATACCTGGAATGCTGTTTTCCCGGGGATCGCAGTGGTGAGTAACCATGCATCATCA
GGAGTACGGATAAAATGCTTGATGGTCGGAAGAGGCATAAATTCCGTCAGCCAGTTTAGTCTGACCATCTCATCTGTAACATCATTGGCAACGCTACCTTTGCCATGTTTCAG
AAACAACTCTGGCGCATCGGGCTTCCCATACAATCGATAGATTGTCGCACCTGATTGCCCGACATTATCGCGAGCCCATTTATACCCATATAAATCAGCATCCATGTTGGAAT
TTAATCGCGGCCTCGAGCAAGACGTTTCCCGTTGAATATGGCTCATAACACCCCTTGTATTACTGTTTATGTAAGCAGACAGTTTTATTGTTCATGATGATATATTTTTATCTTG
TGCAATGTAACATCAGAGATTTTGAGACACAACGTGGCTTTCCCCCCCCCCCCCTGCAGGTCTCGGGCTATTCTTTTGATTTATAAGGGATTTTGCCGATTTCGGCCTATTGG
TTAAAAAATGAGCTGATTTAACAAAAATTTAACGCGAATTTTAACAAAATATTAACGTTTACAATTTAAATATTTGCTTATACAATCTTCCTGTTTTTGGGGCTTTTCTGATTATCA

ACCGGGGTACATATGATTGACATGCTAGTTTTACGATTACCGTTCATCGATTCTCTTGTTTGCTCCAGACTCTCAGGCAATGACCTGATAGCCTTTGTAGACCTCTCAAAAATA
GCTACCCTCTCCGGCATGAATTTATCAGCTAGAACGGTTGAATATCATATTGATGGTGATTTGACTGTCTCCGGCCTTTCTCACCCTTTTGAATCTTTACCTACACATTACTCA
GGCATTGCATTTAAAATATATGAGGGTTCTAAAAATTTTTATCCTTGCGTTGAAATAAAGGCTTCTCCCGCAAAAGTATTACAGGGTCATAATGTTTTTGGTACAACCGATTTAG
CTTTATGCTCTGAGGCTTTATTGCTTAATTTTGCTAATTCTTTGCCTTGCCTGTATGATTTATTGGATGTT

Scaffold sequence for folding with 15 different staple sequences with three ~2kb DNA blocks from Genewiz where block 01 is shown in orange, block 02 in blue and block 03 in green were Gibson cloned into M13K07 RF (black):

AACGCTACTACTATTAGTAGAATTGATGCCACCTTTTCAGCTCGCGCCCCAAATGAAAATATAGCTAAACAGGTTATTGACCATTTGCGAAATGTATCTAATGGTCAAACTAAA
TCTACTCGTTCGCAGAATTGGGAATCAACTGTTACATGGAATGAAACTTCCAGACACCGTACTTTAGTTGCATATTTAAAACATGTTGAGCTACAGCACCAGATTCAGCAATTA
AGCTCTAAGCCATCCGCAAAAATGACCTCTTATCAAAAGGAGCAATTAAAGGTACTCTCTAATCCTGACCTGTTGGAGTTTGCTTCCGGTCTGGTTCGCTTTGAAGCTCGAAT
TAAAACGCGATATTTGAAGTCTTTCGGGCTTCCTCTTAATCTTTTTGATGCAATCCGCTTTGCTTCTGACTATAATAGTCAGGGTAAAGACCTGATTTTTGATTTATGGTCATTC
TCGTTTTCTGAACTGTTTAAAGCATTTGAGGGGGATTCAATGAATATTTATGACGATTCCGCAGTATTGGACGCTATCCAGTCTAAACATTTTACTATTACCCCCTCTGGCAAA
ACTTCTTTTGCAAAAGCCTCTCGCTATTTTGGTTTTTATCGTCGTCTGGTAAACGAGGGTTATGATAGTGTTGCTCTTACTATGCCTCGTAATTCCTTTTGGCGTTATGTATCTG
CATTAGTTGAATGTGGTATTCCTAAATCTCAACTGATGAATCTTTCTACCTGTAATAATGTTGTTCCGTTAGTTCGTTTTATTAACGTAGATTTTTCTTCCCAACGTCCTGACTGG
TATAATGAGCCAGTTCTTAAAATCGCATAAGGTAATTCACAATGATTAAAGTTGAAATTAAACCATCTCAAGCCCAATTTACTACTCGTTCTGGTGTTTCTCGTCAGGGCAAGC
CTTATTCACTGAATGAGCAGCTTTGTTACGTTGATTTGGGTAATGAATATCCGGTTCTTGTCAAGATTACTCTTGATGAAGGTCAGCCAGCCTATGCGCCTGGTCTGTACACC
GTTCATCTGTCCTCTTTCAAAGTTGGTCAGTTCGGTTCCCTTATGATTGACCGTCTGCGCCTCGTTCCGGCTAAGTAACATGGAGCAGGTCGCGGATTTCGACACAATTTATC
AGGCGATGATACAAATCTCCGTTGTACTTTGTTTCGCGCTTGGTATAATCGCTGGGGGTCAAAGATGAGTGTTTTAGTGTATTCTTTCGCCTCTTTCGTTTTAGGTTGGTGCCT
TCGTAGTGGCATTACGTATTTTACCCGTTTAATGGAAACTTCCTCATGAAAAAGTCTTTAGTCCTCAAAGCCTCTGTAGCCGTTGCTACCCTCGTTCCGATGCTGTCTTTCGCT
GCTGAGGGTGACGATCCCGCAAAAGCGGCCTTTAACTCCCTGCAAGCCTCAGCGACCGAATATATCGGTTATGCGTGGGCGATGGTTGTTGTCATTGTCGGCGCAACTATC
GGTATCAAGCTGTTTAAGAAATTCACCTCGAAAGCAAGCTGATAAACCGATACAATTAAAGGCTCCTTTTGGAGCCTTTTTTTTTGGAGATTTTCAACGTGAAAAAATTATTATT
CGCAATTCCTTTAGTTGTTCCTTTCTATTCTCACTCCGCTGAAACTGTTGAAAGTTGTTTAGCAAAACCCCATACAGAAAATTCATTTACTAACGTCTGGAAAGACGACAAAACT
TTAGATCGTTACGCTAACTATGAGGGTTGTCTGTGGAATGCTACAGGCGTTGTAGTTTGTACTGGTGACGAAACTCAGTGTTACGGTACATGGGTTCCTATTGGGCTTGCTAT
CCCTGAAAATGAGGGTGGTGGCTCTGAGGGTGGCGGTTCTGAGGGTGGCGGTTCTGAGGGTGGCGGTACTAAACCTCCTGAGTACGGTGATACACCTATTCCGGGCTATA
CTTATATCAACCCTCTCGACGGCACTTATCCGCCTGGTACTGAGCAAAACCCCGCTAATCCTAATCCTTCTCTTGAGGAGTCTCAGCCTCTTAATACTTTCATGTTTCAGAATA
ATAGGTTCCGAAATAGGCAGGGGGCATTAACTGTTTATACGGGCACTGTTACTCAAGGCACTGACCCCGTTAAAACTTATTACCAGTACACTCCTGTATCATCAAAAGCCATG
TATGACGCTTACTGGAACGGTAAATTCAGAGACTGCGCTTTCCATTCTGGCTTTAATGAGGATCCATTCGTTTGTGAATATCAAGGCCAATCGTCTGACCTGCCTCAACCTCC
TGTCAATGCTGGCGGCGGCTCTGGTGGTGGTTCTGGTGGCGGCTCTGAGGGTGGTGGCTCTGAGGGTGGCGGTTCTGAGGGTGGCGGCTCTGAGGGAGGCGGTTCCGG
TGGTGGCTCTGGTTCCGGTGATTTTGATTATGAAAAGATGGCAAACGCTAATAAGGGGGCTATGACCGAAAATGCCGATGAAAACGCGCTACAGTCTGACGCTAAAGGCAAA
CTTGATTCTGTCGCTACTGATTACGGTGCTGCTATCGATGGTTTCATTGGTGACGTTTCCGGCCTTGCTAATGGTAATGGTGCTACTGGTGATTTTGCTGGCTCTAATTCCCA
AATGGCTCAAGTCGGTGACGGTGATAATTCACCTTTAATGAATAATTTCCGTCAATATTTACCTTCCCTCCCTCAATCGGTTGAATGTCGCCCTTTTGTCTTTAGCGCTGGTAA
ACCATATGAATTTTCTATTGATTGTGACAAAATAAACTTATTCCGTGGTGTCTTTGCGTTTCTTTTATATGTTGCCACCTTTATGTATGTATTTTCTACGTTTGCTAACATACTGC
GTAATAAGGAGTCTTAATCATGCCAGTTCTTTTGGGTATTCCGTTATTATTGCGTTTCCTCGGTTTCCTTCTGGTAACTTTGTTCGGCTATCTGCTTACTTTTCTTAAAAAGGGC
TTCGGTAAGATAGCTATTGCTATTTCATTGTTTCTTGCTCTTATTATTGGGCTTAACTCAATTCTTGTGGGTTATCTCTCTGATATTAGCGCTCAATTACCCTCTGACTTTGTTCA
GGGTGTTCAGTTAATTCTCCCGTCTAATGCGCTTCCCTGTTTTTATGTTATTCTCTCTGTAAAGGCTGCTATTTTCATTTTTGACGTTAAACAAAAAATCGTTTCTTATTTGGATT
GGGATAAATAATATGGCTGTTTATTTTGTAACTGGCAAATTAGGCTCTGGAAAGACGCTCGTTAGCGTTGGTAAGATTCAGGATAAAATTGTAGCTGGGTGCAAAATAGCAAC
TAATCTTGATTTAAGGCTTCAAAACCTCCCGCAAGTCGGGAGGTTCGCTAAAACGCCTCGCGTTCTTAGAATACCGGATAAGCCTTCTATATCTGATTTGCTTGCTATTGGGC
GCGGTAATGATTCCTACGATGAAAATAAAAACGGCTTGCTTGTTCTCGATGAGTGCGGTACTTGGTTTAATACCCGTTCTTGGAATGATAAGGAAAGACAGCCGATTATTGAT
TGGTTTCTACATGCTCGTAAATTAGGATGGGATATTATTTTTCTTGTTCAGGACTTATCTATTGTTGATAAACAGGCGCGTTCTGCATTAGCTGAACATGTTGTTTATTGTCGTC
GTCTGGACAGAATTACTTTACCTTTTGTCGGTACTTTATATTCTCTTATTACTGGCTCGAAAATGCCTCTGCCTAAATTACATGTTGGCGTTGTTAAATATGGCGATTCTCAATT
AAGCCCTACTGTTGAGCGTTGGCTTTATACTGGTAAGAATTTGTATAACGCATATGATACTAAACAGGCTTTTTCTAGTAATTATGATTCCGGTGTTTATTCTTATTTAACGCCT
TATTTATCACACGGTCGGTATTTCAAACCATTAAATTTAGGTCAGAAGATGAAATTAACTAAAATATATTTGAAAAAGTTTTCTCGCGTTCTTTGTCTTGCGATTGGATTTGCATC
AGCATTTACATATAGTTATATAACCCAACCTAAGCCGGAGGTTAAAAAGGTAGTCTCTCAGACCTATGATTTTGATAAATTCACTATTGACTCTTCTCAGCGTCTTAATCTAAGC
TATCGCTATGTTTTCAAGGATTCTAAGGGAAAATTAATTAATAGCGACGATTTACAGAAGCAAGGTTATTCACTCACATATATTGATTTATGTACTGTTTCCATTAAAAAAGGTA
ATTCAAATGAAATTGTTAAATGTAATTAATTTTGTTTTCTTGATGTTTGTTTCATCATCTTCTTTTGCTCAGGTAATTGAAATGAATAATTCGCCTCTGCGCGATTTTGTAACTTGG
TATTCAAAGCAATCAGGCGAATCCGTTATTGTTTCTCCCGATGTAAAAGGTACTGTTACTGTATATTCATCTGACGTTAAACCTGAAAATCTACGCAATTTCTTTATTTCTGTTTT
ACGTGCTAATAATTTTGATATGGTTGGTTCAATTCCTTCCATAATTCAGAAGTATAATCCAAACAATCAGGATTATATTGATGAATTGCCATCATCTGATAATCAGGAATATGAT
GATAATTCCGCTCCTTCTGGTGGTTTCTTTGTTCCGCAAAATGATAATGTTACTCAAACTTTTAAAATTAATAACGTTCGGGCAAAGGATTTAATACGAGTTGTCGAATTGTTTG
TAAAGTCTAATACTTCTAAATCCTCAAATGTATTATCTATTGACGGCTCTAATCTATTAGTTGTTAGTGCACCTAAAGATATTTTAGATAACCTTCCTCAATTCCTTTCTACTGTT
GATTTGCCAACTGACCAGATATTGATTGAGGGTTTGATATTTGAGGTTCAGCAAGGTGATGCTTTAGATTTTTCATTTGCTGCTGGCTCTCAGCGTGGCACTGTTGCAGGCGG
TGTTAATACTGACCGCCTCACCTCTGTTTTATCTTCTGCTGGTGGTTCGTTCGGTATTTTTAATGGCGATGTTTTAGGGCTATCAGTTCGCGCATTAAAGACTAATAGCCATTC
AAAAATATTGTCTGTGCCACGTATTCTTACGCTTTCAGGTCAGAAGGGTTCTATCTCTGTTGGCCAGAATGTCCCTTTTATTACTGGTCGTGTGACTGGTGAATCTGCCAATGT
AAATAATCCATTTCAGAGACACTCGCACAATCGGCTCCAATGTAATGTTTGGTTTTCCGATTGAGCGTCAAAATGTAGGTATTTCCATGAGCGTTTTTCCTGTTGCAATGGCTG
GCGGTAATATTGTTCTGGATATTACCAGCAAGGCCGATAGTTTGAGTTCTTCTACTCAGGCAAGTGATGTTATTACTAATCAAAGAAGTATTGCTACAACGGTTAATTTGCGTG
ATGGACAGACTCTTTTACTCGGTGGCCTCACTGATTATAAAAACACTTCTCAAGATTCTGGCGTACCGTTCCTGTCTAAAATCCCTTTAATCGGCCTCCTGTTTAGCTCCCGCT
CTGATTCCAACGAGGAAAGCACGTTATACGTGCTCGTCAAAGCAACCATAGTACGCGCCCTGTAGCGGCGCATTAAGCGCGGCGGGTGTGGTGGTTACGCGCAGCGTGAC
CGCTACACTTGCCAGCGCCCTAGCGCCCGCTCCTTTCGCTTTCTTCCCTTCCTTTCTCGCCACGTTCGCCGGCTTTCCCCGTCAAGCTCTAAATCGGGGGCTCCCTTTAGGG
TTCCGATTTAGTGCTTTACGGCACCTCGACCCCAAAAAACTTGATTTGGGTGATGGTTCACGTAGTGGGCCATCGCCCTGATAGACGGTTTTTCGCCCTTTGACGTTGGAGT
CCACGTTCTTTAATAGTGGACTCTTGTTCCAAACTGGAACAACACTCAACCCTATCTCGGGACGGATCGCTTCATGTGGCAGGAGAAAAAAGGCTGCACCGGTGCGTCAGCA
GAATATGTGATACAGGATATATTCCGCTTCCTCGCTCACTGACTCGCTACGCTCGGTCGTTCGACTGCGGCGAGCGGAAATGGCTTACGAACGGGGCGGAGATTTCCTGGA
AGATGCCAGGAAGATACTTAACAGGGAAGTGAGAGGGCCGCGGCATCCGCGCGAAGCGCGGATGCCTCGCCACATTCTAAGTGCGACCGTAAGATCGCACAATCGGCTCC
AATGTGATCATCATCGTAGAAGCGATAGGCCTTTTGCGATATTCTAAAACGCTCTATCCTAGTCGGTTCACACGCCTTCAAGCACGCAGTACTAATGCTTACAGAATCCCTTA
CTACTAGCGTGCGACTGAAGAGTAGCACATAGGTTACGCGATTGCTCCAGGGAGATATCACTTGTCATCGTAATGTTTGTTTCAGTTGCGATTACTAGCTACATGTCGGTAGG
CCTTACGTAGTGATTCAACTACATTCTGCGCATAGCACACGTAAGACTAACCCGTTCTTGCTGCGCAGCGGGGACCGGACCTCGCCACTTGTGTCAAAATGTATGAGGAATA
CGAAATAGACATGGTGTCGGAACATGTAAATAGGCAGGTTTTCAATCGACCCGGCGTTCACTTGTGGTCAACCTTGGGGTGCGACAGGCCTTTCGCCACCCTTATCACGCTT
AGGGTAGTTAGCACACTTGTTGGCCCAATCGACCTGCGCAACGACACCTCCAGGTATGCAGTACTAGCAACGCTCATTCTAAATTAAACATAGTGGAATGTTTTGCCCTATTC
CTGTAATGTTTTACCGCTGTTGAGACAATCACTCCACGGCCGAATGAGCGTGGTTCTTTAGACGGTAACGGTAAGCTAAACTTTGCTACATTTCATCGTAGCTTGAAGGCCTT
TTGCGATTATCCTATAGGTTATACCTCGAGTCTGGAATGTTTTCACTTGCGCCGTTCTTGTCATCGTCAATGGAAGTAACAAAGCGATGTCGGTTACAGAATTAGCACACCAG
TTACCCTTACCCTTATCAGGATTTACCCGCCTCATCGTATGCAGGCGCCTGACACACGCCTTCAAGCACGCAGTACTAATGCTTTTCTGGACCTGGGTCTACATTCTATCAAC
TAACCCTATGCAGTTGTGTCAGGAATCCTGCGCATGTGTGTATTCTAAATCGTAGGTCTAGCTTTGTATCTACATTTGGTCAACTATCAGGCAGTTTCAGTCGAACAACGACCT
TCTTTGGGGTTTTCTTGCGATTACCGCTGGTCGCACTATCAAAAAATGTTTCCGGGCGCGATTAGGAATCCTTGTTGTTAGCGATCAGTTTATGAGGAATACGAAATAGACAT
GGTGTCGGAACGTGTGGGTAACGGTGTCGGTTTTTGTGAATGTTTGGAGATATATCTTATATGCAGGGCCGTTGTTGAGACCGGCGTAATGTTTCGTAAGCCTGCGCAACAC
GTGACGTAGTTATCCCGCTCGATTCAAATAGGCATCACTTGAAACTTAAATGTTTTTCCGGGTTAGACGAATCGACTACCTCGCGGTAAATTGCGAGCGTAAGAACACGTGC
CAGTTATGTGTGTCGGAGATCAATCACTCCACGGCCGAATGAGCGTGGTTCTCGCAGTTGTTTGCGAGCTTGATCGAACAGGGTAGTATGCCTCTCGTCTGGCCAGAACCG

GACCATGCAGGCGCCTGACGGCTACGAAGACAGGCCGTTCTTGTTGACGCTTAACATACTGGCATGTCGGTGCTCCAGGAACTTATACGGTGTTCTGGATGAAGAGGCGG
GGATTGTATCACGACCTTTTGTATACGACACATTAAACATAGTGGGCTCCAGCACACGCCTTCAAGCACGCAGTACTAATGCTTGATCATCTATTGTACCTTGGGCTCCAGGA
TCGGCTACGCTTATATTGTAAAACTTAAAAATGTTCAGTTTGTAACGGAAATTCGGATCATCAGGAATCTATTGTAATGCCTCTTTCGTCGGTTTTGTATCTTTTGTGTAGCACA
CGAGGGATAAGCTATGCCCTATTGTGTCGAAGACACCTTATCGATTCAACCAATGTTCACTTGGGTCGCAACTGGCATAAGCTAATGAGGAATACGAAATAGACATGGTGTC
GGAAAATGTTTTATGCAGTGTGTGTCTGCGCATAGGTTAACGTAGTCTACATTAATGTTTCCCTTACTTCCTGTGCTCCAGCGGACCAAGGCCTTTATGCAGGCCAGAAACAG
AATATGAAGGAATCTCGTCTGCCTTATCATGCCTCAGGATATGATCATCGTGCGACCCAGTTATCACTTGTTTTGTGGTTTCAGTACCTCGGTTCTTGACAGAATCCTTATCAG
GATATCAATCACTCCACGGCCGAATGAGCGTGGTTCTCGGACCATATCCTAGTCGGTTTGGTCAAGTGCGACCGTAAGAACACGTGATAGTGGAGCTTGACGTAAGATTAG
CGACCTGGGTTTGCGAGTATCTTACGGTAAATTTGTCGAACACGTAAGAGGTCGCACCTTGGGCATGTAACATGTAATTAGCGAATTAAACTCGAACAGGGTAGTATTCTAAA
GTCTGGCAATGGAAACGCTCCGAGGGAAAATTCGCACACGCCTTCAAGCACGCAGTACTAATGCTTGAACTTATCGCCACTACTAGCAGTAACAAGGCCTTTTGCGATACGA
CACGGTCGCACCTTGGGCCTGGGTAGGATTTACCCGCCATTAAACATTCTAAGTGCGACGGGTAGTTGAAGTGTGTACGACACTACCTCGTTGCGATATAGTGGGTTTGCG
AACTTTGAGGATATATAGGCATCATCGTCTCCAGGAAGCGATTAGCACAGTCTAGCTACCTCGGGTTTTCTACGGTGATGAGGAATACGAAATAGACATGGTGTCGGAACGG
CTACAAGCGATTAGGTTATCTTTGGGATCATCATCGTAGAGTCTGGTGTCGGTCTTGTTGCGGACCACGGAGATCGCAGTTAGCTTGAAAGCGATTAGGTTACTCCAGGTAC
CGGCGAGGTTTCAGCTATCAAATCGTAGCCAGTTACGGCTACCGTGTGGCGAGGGACGTAAGCACTGGCACAATGGATCGCACAGCTCCAGCGTAAGCTTGTATCATCGTA
GTATCTTACAATCACTCCACGGCCGAATGAGCGTGGTTCTCGGTAAATCGTCTGGCCAGAAAAACTTAGAAGACAGGCCGTTAGTAACAGTCTAGCTCTTTGGAAATTCGGA
ACTTATACGGTGCCCTTACTTGTGTCAAAATGTACGTAGTGATTCAAACGCTTAACTGGCATGTCGGTCTTGTTGATAGTGGAGCTTGACCAGTTACGGAGATCGCAGTTGTT
TGCGAACGCTCTATCCTAGTCGGTTCAATCACTCCACGGCCGAATGAGCGTGGTTCTACAGAATTGAAGAGTACTAGCTCGCACACGGAGATCGTGTGGCCCTTACGGCCG
TTATTAAACATAGGCACGTGTGGTATTGTAACGACCTTTCTGTTGAGATTGTATCCGGAGATCGCAGTTCCTTGGGAATGTTTTCACTTGTCAGTTTCGCAGTTGTTTGCGTCG
CCACGCGGGGACCGGACCATGAGGAATACGAAATAGACATGGTGTCGGAAAATCGACGGGTAGTGGTTTTCCATGTAATATCTTATTCTGGACTGCGCATAGGTTAGGTCG
CAGTTTGCGAACTTTGGCCAGAATTAGACGTGAGGCGGGGATCGTCTGAGGATTTACCCGCCTTGCGAGTACCTCGTATGCAGCGCCTGACGGTAAATCGTCTGAATGTTT
ACGACACCTCCAGGCACACGCCTTCAAGCACGCAGTACTAATGCTTAGTCTGGGTAACGGTAAGCTAGCCAGAATTAGACGATGCAGGCCTTATCTTTGTATATGCCTCTCG
CACATATCTTACGGTAAACCGGACCACACGTTGTGAATCGACCGCGATTCTCCAGGATCGGCTCCAATGTACGACCTCTACATTTCATCGTGTTGAGATTGTATCTATTGTAA
TAGGCACAATGGAAGTAACACAATCACTCCACGGCCGAATGAGCGTGGTTCTGTCTAGCCTAACCCCTTGTTGACTGGCACCTGGGTTACCGCTTCGCACAAGGCCTTACG
CTTAGGAGATATTAGCGAACGACACCTAACCCCGCCTGCAGGGTTCTTGTTCCTGTCATGTAATTCCGGGAAGCGATTAGCACAATCGGCTAAAATGTTTAGCGATGTCGGT
TCGAACACGCGATTTCTTTGGATGAGGAATACGAAATAGACATGGTGTCGGAACGTAAGCGGTCGCAACACGTGCTTGTTGCCAATGTTTGTGTCCCAATGTAAAATGTATC
GTAGCCGGCGTTAAGCTATGCCCTATGGTCAAATGCCTGTATTATCTTATCAGTTTGTAACGGTGCCCTAGAAGACATTGTGTCGGAGATACGTGTGGATCGGCTATAGGCA
TTGCGAGTGTGTGTAAACTTACACACGCCTTCAAGCACGCAGTACTAATGCTTGAAGACAGGCCGTTATGCCTCCATGTAATTAGCGATCAGTTTTTCCGGGCCGGCGTAAA
CTTATTCCTGTAACTTTGTTCTGGACGGTAAATCGTCTGTTTGTGACCTGCCAGAAATGCAGGCGCCTGAAGGATTTTCTTTGGCGTAAGCTGCCCTACGCCTGATTCTGGAC
CTGGGTACGACCTTTTGTATTATCTTACAATCACTCCACGGCCGAATGAGCGTGGTTCTCGGTAAAAGGAATCACGCTTAAGGATATCCAGTTAATGCAGGTTCCTGTGCTCC
AGGTCTAGCACCCGCCTTCCGGGTTAGACGTTGTATCAATATTGCGAGGTTGAGACCGGCGTAATGTTTAGTAACAGATCATCTGAAGAGTTAGACGCCCTTACGGAGATAG
ATTCAATTTTGTGCCTTGGGATGAGGAATACGAAATAGACATGGTGTCGGAAATTAAACACTGGCAACGTAGTTTCCGGGTACGGTGGTTGAGAGCGGGGAACGTAGTCAAT
GGAGGTTTTCTCGAACATACCGCTTGAAGAGATAATTCTGGATGTGTGTGGGTAGTCCTGGGTAGTCTGGGATTCAATTGCGATTACCGCTCGGCTACAGGATATGTTTCAG
TGTCGGTGTTCTTGCACACGCCTTCAAGCACGCAGTACTAATGCTTTGGTCAATTGTGTCAAAATGTCGGACCAAGGATTTACCCGCCATTCTAAGTTCTTGTGGTCAAAGGA
TATGTCGGTTACAGAATTCAGTTTCTAACCGATTCGTGTGGCGGAGATTATCCTACGGACCACGAGGGACTAACCCGTGCGACCGTAAGAAACTTTGCGAGGGAAAATTCGC
CCTTACAGTCTGGCAATGGACAATCACTCCACGGCCGAATGAGCGTGGTTCTAGTAACAAGCTTGACCAGTTAGAACTTATGCCCTATTCCTGTCTATCAAAATCGACCGCG
ATTAAATTCGTACTAGCAACGCTCCGCAGTTCCGGAGGGGAGTTTGCGACACGTGTCGCCACGAACTTAGTTTCAGCCGGACCAGGAATCGAACTTACCGGCGTTACGGTG
TATTGTAATAGTGGGTCTAGCATGAGGAATACGAAATAGACATGGTGTCGGAATCTTTGGGCCAGAATTGTATCCGGCTACCGAGGGAAAATTCGTTTTGTGAGGATTTACC
CGCCTACGGTGAGGAATCTTTTGTGGTAACGGACTGGTTGTATAAGCTAGGAGATACTATCAACGGCTACCTCCAGGAACTTTGACACGTGAGCTTGACGGACCAGGTCGC
ACTATCAATCGTCTGCGTAAGCCACACGCCTTCAAGCACGCAGTACTAATGCTTAAACTTAGAAGACAGGCCGTTCCTTGGGATTAAACATAGTGGTCGAACAGGGTAGTAC
GACACTCGCACAATCGGCTCCAATGTAATGTTTGGTTTTCTCCATCCGCGCGAAGCGCGGATGGAGCTCGCCCCGGGGATCGACCAGTTGGTGATTTTGAACTTTTGCTTTG
CCACGGAACGGTCTGCGTTGTCGGGAAGATGCGTGATCTGATCCTTCAACTCAGCAAAAGTTCGATTTATTCAACAAAGCCGCCGTCCCGTCAAGTCAGCGTAATGCTCTGC
CAGTGTTACAACCAATTAACCAATTCTGATTAGAAAAACTCATCGAGCATCAAATGAAACTGCAATTTATTCATATCAGGATTATCAATACCATATTTTTGAAAAAGCCGTTTCT
GTAATGAAGGAGAAAACTCACCGAGGCAGTTCCATAGGATGGCAAGATCCTGGTATCGGTCTGCGATTCCGACTCGTCCAACATCAATACAACCTATTAATTTCCCCTCGTC
AAAAATAAGGTTATCAAGTGAGAAATCACCATGAGTGACGACTGAATCCGGTGAGAATGGCAAAAGCTTATGCATTTCTTTCCAGACTTGTTCAACAGGCCAGCCATTACGCT
CGTCATCAAAATCACTCGCATCAACCAAACCGTTATTCATTCGTGATTGCGCCTGAGCGAGACGAAATACGCGATCGCTGTTAAAAGGACAATTACAAACAGGAATCGAATG
CAACCGGCGCAGGAACACTGCCAGCGCATCAACAATATTTTCACCTGAATCAGGATATTCTTCTAATACCTGGAATGCTGTTTTCCCGGGGATCGCAGTGGTGAGTAACCAT
GCATCATCAGGAGTACGGATAAAATGCTTGATGGTCGGAAGAGGCATAAATTCCGTCAGCCAGTTTAGTCTGACCATCTCATCTGTAACATCATTGGCAACGCTACCTTTGCC
ATGTTTCAGAAACAACTCTGGCGCATCGGGCTTCCCATACAATCGATAGATTGTCGCACCTGATTGCCCGACATTATCGCGAGCCCATTTATACCCATATAAATCAGCATCCA
TGTTGGAATTTAATCGCGGCCTCGAGCAAGACGTTTCCCGTTGAATATGGCTCATAACACCCCTTGTATTACTGTTTATGTAAGCAGACAGTTTTATTGTTCATGATGATATAT
TTTTATCTTGTGCAATGTAACATCAGAGATTTTGAGACACAACGTGGCTTTCCCCCCCCCCCCCTGCAGGTCTCGGGCTATTCTTTTGATTTATAAGGGATTTTGCCGATTTCG
GCCTATTGGTTAAAAAATGAGCTGATTTAACAAAAATTTAACGCGAATTTTAACAAAATATTAACGTTTACAATTTAAATATTTGCTTATACAATCTTCCTGTTTTTGGGGCTTTT
CTGATTATCAACCGGGGTACATATGATTGACATGCTAGTTTTACGATTACCGTTCATCGATTCTCTTGTTTGCTCCAGACTCTCAGGCAATGACCTGATAGCCTTTGTAGACCT
CTCAAAAATAGCTACCCTCTCCGGCATGAATTTATCAGCTAGAACGGTTGAATATCATATTGATGGTGATTTGACTGTCTCCGGCCTTTCTCACCCTTTTGAATCTTTACCTAC
ACATTACTCAGGCATTGCATTTAAAATATATGAGGGTTCTAAAAATTTTTATCCTTGCGTTGAAATAAAGGCTTCTCCCGCAAAAGTATTACAGGGTCATAATGTTTTTGGTACA
ACCGATTTAGCTTTATGCTCTGAGGCTTTATTGCTTAATTTTGCTAATTCTTTGCCTTGCCTGTATGATTTATTGGATGTT

Scaffold sequence for folding with 20 different staple sequences with three ~2kb DNA blocks from Genewiz where block 01 is shown in orange, block 02 in blue and block 03 in green were Gibson cloned into M13K07 RF (black):

AACGCTACTACTATTAGTAGAATTGATGCCACCTTTTCAGCTCGCGCCCCAAATGAAAATATAGCTAAACAGGTTATTGACCATTTGCGAAATGTATCTAATGGTCAAACTAAA
TCTACTCGTTCGCAGAATTGGGAATCAACTGTTACATGGAATGAAACTTCCAGACACCGTACTTTAGTTGCATATTTAAAACATGTTGAGCTACAGCACCAGATTCAGCAATTA
AGCTCTAAGCCATCCGCAAAAATGACCTCTTATCAAAAGGAGCAATTAAAGGTACTCTCTAATCCTGACCTGTTGGAGTTTGCTTCCGGTCTGGTTCGCTTTGAAGCTCGAAT
TAAAACGCGATATTTGAAGTCTTTCGGGCTTCCTCTTAATCTTTTTGATGCAATCCGCTTTGCTTCTGACTATAATAGTCAGGGTAAAGACCTGATTTTTGATTTATGGTCATTC
TCGTTTTCTGAACTGTTTAAAGCATTTGAGGGGGATTCAATGAATATTTATGACGATTCCGCAGTATTGGACGCTATCCAGTCTAAACATTTTACTATTACCCCCTCTGGCAAA
ACTTCTTTTGCAAAAGCCTCTCGCTATTTTGGTTTTTATCGTCGTCTGGTAAACGAGGGTTATGATAGTGTTGCTCTTACTATGCCTCGTAATTCCTTTTGGCGTTATGTATCTG
CATTAGTTGAATGTGGTATTCCTAAATCTCAACTGATGAATCTTTCTACCTGTAATAATGTTGTTCCGTTAGTTCGTTTTATTAACGTAGATTTTTCTTCCCAACGTCCTGACTGG
TATAATGAGCCAGTTCTTAAAATCGCATAAGGTAATTCACAATGATTAAAGTTGAAATTAAACCATCTCAAGCCCAATTTACTACTCGTTCTGGTGTTTCTCGTCAGGGCAAGC
CTTATTCACTGAATGAGCAGCTTTGTTACGTTGATTTGGGTAATGAATATCCGGTTCTTGTCAAGATTACTCTTGATGAAGGTCAGCCAGCCTATGCGCCTGGTCTGTACACC
GTTCATCTGTCCTCTTTCAAAGTTGGTCAGTTCGGTTCCCTTATGATTGACCGTCTGCGCCTCGTTCCGGCTAAGTAACATGGAGCAGGTCGCGGATTTCGACACAATTTATC
AGGCGATGATACAAATCTCCGTTGTACTTTGTTTCGCGCTTGGTATAATCGCTGGGGGTCAAAGATGAGTGTTTTAGTGTATTCTTTCGCCTCTTTCGTTTTAGGTTGGTGCCT
TCGTAGTGGCATTACGTATTTTACCCGTTTAATGGAAACTTCCTCATGAAAAAGTCTTTAGTCCTCAAAGCCTCTGTAGCCGTTGCTACCCTCGTTCCGATGCTGTCTTTCGCT
GCTGAGGGTGACGATCCCGCAAAAGCGGCCTTTAACTCCCTGCAAGCCTCAGCGACCGAATATATCGGTTATGCGTGGGCGATGGTTGTTGTCATTGTCGGCGCAACTATC
GGTATCAAGCTGTTTAAGAAATTCACCTCGAAAGCAAGCTGATAAACCGATACAATTAAAGGCTCCTTTTGGAGCCTTTTTTTTTGGAGATTTTCAACGTGAAAAAATTATTATT
CGCAATTCCTTTAGTTGTTCCTTTCTATTCTCACTCCGCTGAAACTGTTGAAAGTTGTTTAGCAAAACCCCATACAGAAAATTCATTTACTAACGTCTGGAAAGACGACAAAACT
TTAGATCGTTACGCTAACTATGAGGGTTGTCTGTGGAATGCTACAGGCGTTGTAGTTTGTACTGGTGACGAAACTCAGTGTTACGGTACATGGTTCCTATTGGGCTTGCTAT
CCCTGAAAATGAGGGTGGTGGCTCTGAGGGTGGCGGTTCTGAGGGTGGCGGTTCTGAGGGTGGCGGTACTAAACCTCCTGAGTACGGTGATACACCTATTCCGGGCTATA

CTTATATCAACCCTCTCGACGGCACTTATCCGCCTGGTACTGAGCAAAACCCCGCTAATCCTAATCCTTCTCTTGAGGAGTCTCAGCCTCTTAATACTTTCATGTTTCAGAATA
ATAGGTTCCGAAATAGGCAGGGGGCATTAACTGTTTATACGGGCACTGTTACTCAAGGCACTGACCCCGTTAAAACTTATTACCAGTACACTCCTGTATCATCAAAAGCCATG
TATGACGCTTACTGGAACGGTAAATTCAGAGACTGCGCTTTCCATTCTGGCTTTAATGAGGATCCATTCGTTTGTGAATATCAAGGCCAATCGTCTGACCTGCCTCAACCTCC
TGTCAATGCTGGCGGCGGCTCTGGTGGTGGTTCTGGTGGCGGCTCTGAGGGTGGTGGCTCTGAGGGTGGCGGTTCTGAGGGTGGCGGCTCTGAGGGAGGCGGTTCCGG
TGGTGGCTCTGGTTCCGGTGATTTTGATTATGAAAAGATGGCAAACGCTAATAAGGGGGCTATGACCGAAAATGCCGATGAAAACGCGCTACAGTCTGACGCTAAAGGCAAA
CTTGATTCTGTCGCTACTGATTACGGTGCTGCTATCGATGGTTTCATTGGTGACGTTTCCGGCCTTGCTAATGGTAATGGTGCTACTGGTGATTTTGCTGGCTCTAATTCCCA
AATGGCTCAAGTCGGTGACGGTGATAATTCACCTTTAATGAATAATTTCCGTCAATATTTACCTTCCCTCCCTCAATCGGTTGAATGTCGCCCTTTTGTCTTTAGCGCTGGTAA
ACCATATGAATTTTCTATTGATTGTGACAAAATAAACTTATTCCGTGGTGTCTTTGCGTTTCTTTTATATGTTGCCACCTTTATGTATGTATTTTCTACGTTTGCTAACATACTGC
GTAATAAGGAGTCTTAATCATGCCAGTTCTTTTGGGTATTCCGTTATTATTGCGTTTCCTCGGTTTCCTTCTGGTAACTTTGTTCGGCTATCTGCTTACTTTTCTTAAAAAGGGC
TTCGGTAAGATAGCTATTGCTATTTCATTGTTTCTTGCTCTTATTATTGGGCTTAACTCAATTCTTGTGGGTTATCTCTCTGATATTAGCGCTCAATTACCCTCTGACTTTGTTCA
GGGTGTTCAGTTAATTCTCCCGTCTAATGCGCTTCCCTGTTTTTATGTTATTCTCTCTGTAAAGGCTGCTATTTTCATTTTTGACGTTAAACAAAAAATCGTTTCTTATTTGGATT
GGGATAAATAATATGGCTGTTTATTTTGTAACTGGCAAATTAGGCTCTGGAAAGACGCTCGTTAGCGTTGGTAAGATTCAGGATAAAATTGTAGCTGGGTGCAAAATAGCAAC
TAATCTTGATTTAAGGCTTCAAAACCTCCCGCAAGTCGGGAGGTTCGCTAAAACGCCTCGCGTTCTTAGAATACCGGATAAGCCTTCTATATCTGATTTGCTTGCTATTGGGC
GCGGTAATGATTCCTACGATGAAAATAAAAACGGCTTGCTTGTTCTCGATGAGTGCGGTACTTGGTTTAATACCCGTTCTTGGAATGATAAGGAAAGACAGCCGATTATTGAT
TGGTTTCTACATGCTCGTAAATTAGGATGGGATATTATTTTTCTTGTTCAGGACTTATCTATTGTTGATAAACAGGCGCGTTCTGCATTAGCTGAACATGTTGTTTATTGTCGTC
GTCTGGACAGAATTACTTTACCTTTTGTCGGTACTTTATATTCTCTTATTACTGGCTCGAAAATGCCTCTGCCTAAATTACATGTTGGCGTTGTTAAATATGGCGATTCTCAATT
AAGCCCTACTGTTGAGCGTTGGCTTTATACTGGTAAGAATTTGTATAACGCATATGATACTAAACAGGCTTTTTCTAGTAATTATGATTCCGGTGTTTATTCTTATTTAACGCCT
TATTTATCACACGGTCGGTATTTCAAACCATTAAATTTAGGTCAGAAGATGAAATTAACTAAAATATATTTGAAAAAGTTTTCTCGCGTTCTTTGTCTTGCGATTGGATTTGCATC
AGCATTTACATATAGTTATATAACCCAACCTAAGCCGGAGGTTAAAAAGGTAGTCTCTCAGACCTATGATTTTGATAAATTCACTATTGACTCTTCTCAGCGTCTTAATCTAAGC
TATCGCTATGTTTTCAAGGATTCTAAGGGAAAATTAATTAATAGCGACGATTTACAGAAGCAAGGTTATTCACTCACATATATTGATTTATGTACTGTTTCCATTAAAAAAGGTA
ATTCAAATGAAATTGTTAAATGTAATTAATTTTGTTTTCTTGATGTTTGTTTCATCATCTTCTTTTGCTCAGGTAATTGAAATGAATAATTCGCCTCTGCGCGATTTTGTAACTTGG
TATTCAAAGCAATCAGGCGAATCCGTTATTGTTTCTCCCGATGTAAAAGGTACTGTTACTGTATATTCATCTGACGTTAAACCTGAAAATCTACGCAATTTCTTTATTTCTGTTTT
ACGTGCTAATAATTTTGATATGGTTGGTTCAATTCCTTCCATAATTCAGAAGTATAATCCAAACAATCAGGATTATATTGATGAATTGCCATCATCTGATAATCAGGAATATGAT
GATAATTCCGCTCCTTCTGGTGGTTTCTTTGTTCCGCAAAATGATAATGTTACTCAAACTTTTAAAATTAATAACGTTCGGGCAAAGGATTTAATACGAGTTGTCGAATTGTTTG
TAAAGTCTAATACTTCTAAATCCTCAAATGTATTATCTATTGACGGCTCTAATCTATTAGTTGTTAGTGCACCTAAAGATATTTTAGATAACCTTCCTCAATTCCTTTCTACTGTT
GATTTGCCAACTGACCAGATATTGATTGAGGGTTTGATATTTGAGGTTCAGCAAGGTGATGCTTTAGATTTTTCATTTGCTGCTGGCTCTCAGCGTGGCACTGTTGCAGGCGG
TGTTAATACTGACCGCCTCACCTCTGTTTTATCTTCTGCTGGTGGTTCGTTCGGTATTTTTAATGGCGATGTTTTAGGGCTATCAGTTCGCGCATTAAAGACTAATAGCCATTC
AAAAATATTGTCTGTGCCACGTATTCTTACGCTTTCAGGTCAGAAGGGTTCTATCTCTGTTGGCCAGAATGTCCCTTTTATTACTGGTCGTGTGACTGGTGAATCTGCCAATGT
AAATAATCCATTTCAGACGATTGAGCGTCAAAATGTAGGTATTTCCATGAGCGTTTTTCCTGTTGCAATGGCTGGCGGTAATATTGTTCTGGATATTACCAGCAAGGCCGATA
GTTTGAGTTCTTCTACTCAGGCAAGTGATGTTATTACTAATCAAAGAAGTATTGCTACAACGGTTAATTTGCGTGATGGACAGACTCTTTTACTCGGTGGCCTCACTGATTATA
AAAACACTTCTCAAGATTCTGGCGTACCGTTCCTGTCTAAAATCCCTTTAATCGGCCTCCTGTTTAGCTCCCGCTCTGATTCCAACGAGGAAAGCACGTTATACGTGCTCGTC
AAAGCAACCATAGTACGCGCCCTGTAGCGGCGCATTAAGCGCGGCGGGTGTGGTGGTTACGCGCAGCGTGACCGCTACACTTGCCAGCGCCCTAGCGCCCGCTCCTTTC
GCTTTCTTCCCTTCCTTTCTCGCCACGTTCGCCGGCTTTCCCCGTCAAGCTCTAAATCGGGGGCTCCCTTTAGGGTTCCGATTTAGTGCTTTACGGCACCTCGACCCCAAAA
AACTTGATTTGGGTGATGGTTCACGTAGTGGGCCATCGCCCTGATAGACGGTTTTTCGCCCTTTGACGTTGGAGTCCACGTTCTTTAATAGTGGACTCTTGTTCCAAACTGGA
ACAACACTCAACCCTATCTCGGGCTATTCTTTTGATTTATAAGGGATTTTGCCGATTTCGGCCTATTGGTTAAAAAATGAGCTGATTTAACAAAAATTTAACGCGAATTTTAA
ACTGACTCGCTACGCTCGGTCGTTCGACTGCGGCGAGCGGAAATGGCTTACGAACGGGGCGGAGATTTCCTGGAAGATGCCAGGAAGATACTTAACAGGGAAGTGAGAGG
GCCGCGGCATCCGCGCGAAGCGCGGATGCCCTACCGCCCATGGGCAATCCCCAAGGTTCGATAGCTGGGTGCAATCAAATAGGGGACAAGAGGATATCCGAGGCTGGAA
TTCCCTCGGTACTGTTTAGCCCAAAGGTAACTAGATGAGGGCTCGCAGCAACTTATGTAGCCTTTATTAAATAAACGGAGCTTATTGTTTGGGCACGCGATTGTGTATACACG
CGGCGGCTTCAAGCGTCGATCCCATCAGGAGGCAAGGTTATAGCGCCGATTGTGAAGCTGCTGCCGCGTAAATTCGGCGCCAAGTTCACGGGGTGGTTCAGTAGACTCGT
TTGTGTAGATAGGGTATTCTCGACGCTCGACTCGTTTTCCTCGGCTATTACGAGCTGCACTTGAAATCGTCCGTGGATGCGTTGCCAGGTCCAACTCTGGAAATTTCGACAC
AGGGTCAGGCGGCTATACATGAAGTCATCAGGGCCATATAGCACCGGTTTGGGAGGCTGGACGAGCTGCAGTCGTAGGCGGAATTGCCTGCCAGCAGTTGTATTTTGCTAT
ACTAACCCAGCATAAGCCGGGTCTAGGTCGGCTTATTTGTTTAGTCGGTACCTGTACGTGTTATGGTTATAGGTTCTTCTGCTATAAAATGTTAGGGTACTTGCGCATTTGCC
ATTACACCCAACTTAATACGAGATTACGTACTCAGAAGGGCATCTTTTTGAGGGTTCAGTAAGGCAAGATCGCGAAGGCTGGAATTCCCCCCAAAGGATCGTGCGACGTGTG
AGCGTTGTTAGAATCAGGGGGTCGACGCTCAGGCAAGCCTTGATTAAATCCGTCCGGAGTAACTAATTAAATTTGTGTACTGGGAGAAACGGAGCAGTCGCTACTGAGCGA
TTCAGGCAAGACCCAGCTCGCCTGGATTGTATATGCATCGAAGAGCCGAGACCTCGGAGATATTGTCGGCTTCAGTAGGAGAAATATTCTCTAGGTCGGCACTTGTAACCCT
GACTCGTCTTCTATTCGGTACTATCGGCTATGGGTGTGATACTTCAGTAGCCATATTGCGGAGGCAGGAGAAAGCGAACGCAGAAAGCATTTTATCAGGCGAATTCCCAGGG
TACAGCGGAATGCGGGAAAATCGTCGGGAAAGGCGGCTCTACCAGGGAATAAGGGCTTATGGGAGCGATGAGGGCTCGCAGCAACTTATGTAGCCTTTCCAGTTTAGGCT
GCGATAAACTACCTATGTTATAGTCGATCCTGCATATTTCATCGGGTAAGTTTGCGCAATGAAGTAAATGTTTTTGTATGACTCGTGTTCCAACTACCAGGGAAGGCCAGTAC
CTATCACAGGGCATCAGGAATTGCTGTTATAGCGGGAAACGTACTCGCTATACCGGCTGGCCGATATAGTAGGAGATAGGGGTTCCAACTGGGAGCTTCTATCAAACAACC
GTGGATGCGTTGCCAGGTCCAACTCTGGAAAATTCCCTCAGTTAATCGCGAGCGAACGAATTGCCACGGACCACAGTCACAGAGCGGGCTATTACCCAGCTCGCCTGACTC
TGAGGGTAGTGGTAAGTGGAATAAGTTCCAAGCCCGACCTTAGATAAACCGCAGACTGGTTCGTAGAGCAGAGATATCACGCGATTTCCTCCCCCACACAGAAAGGTGATA
CGCATAAGCTGTACGTGTTATGCGCAGACTTTGCCATTACACCCAACTTAATACGAGATTACTCGACACACAATAAGCACCGCCGGGTCTGGGTGCTAGGCGGCACAATAAA
TTGCTCAAGGTTTGGGAGCAGAAGGGGCAAGTGCTCGACACTACCAGCACAATAGCATAAGGTCCATACTGCCCCCACATACCTATTTGTGTACGGCGGTCATCTTTGTTCT
TCCAATCCCGGGTAGTTAAATTCGGGGTGGAATCAAACATCAGGAGCGGAAGACCTTAATTGGCCGATTGTATATGCATCGAAGAGCCGAGACCTCGAGATTGGTAGGTCG
CGGGTCTGACTCGTGATCGTGAGTTCACAGTTGTAGTTATAGCCATGGGTGCTATACGCAGACAGGGGAGAGGCTGGTAGGTCGCAGAGCGGCCGGTGACTGTAACCCTA
CAGTCAGCAGTCGACGGACCCGAGATACTCGACAGTTTGGGTGTGAAGCATCAGGCAAAATCCGCCGATCGACGTGGACGCTCGCCGGTGGCAGTCGCGAGATAGATGA
GGGCTCGCAGCAACTTATGTAGCCTTTAGGGGAGGGAAGAAATACTGCGCCATATGTTTGGGGATAGGGGTATTTTTGCCAGCGGCGCCAGATAGGGGGGCTTATGTCGG
CAGTAGGATGCATATCCGATATCATTAAGACCGGATAGGGAGCGGAAAGCACCGATTTCGAATTTCGAGGGCTTATAACCCAAAGACCGATGAGTTTCGGTACTGAGCGTCC
TTGATTCGCCAGCGGCGGTGCAAGTGCCGTGGATGCGTTGCCAGGTCCAACTCTGGAATGGTTCGCTACCGCCTGCTGCTAAATCCAGGCTGGAATTCCCACTTACTTGTT
AGAATCAGGGTGTCGGCCTACTGAGCGATTCCTGTACGTCGGTACGTTTGGGATGAGTTCACGCGGTCTACTTACTCGACGTGAATTCCCTGTTATGTCAGTTATTGAGGGC
GAGATACACAGGGTTAGGCATGCTCTCGTCCGGATTGTGTATATGGGTCGCGAGCGTGCGCCGAGTAGAGCATTTGCCATTCACACCCAACTTAATACGAGATTAACTCTGAGTCC
GGAGATCGTGCATTTTACTCGACATATCGGCTGAGCGTGATAAACGGAATAAAGGGGAGCAAACAAAATTCCCATCGCGAGTCCGGAGATCGTGTGCTCTCAGGGTGGGTC
CGCCGATGGAGAAATATCGGCCTGGGAGACTCTGACCAGTTTCGGCGGTTTTGTATGACCTTACCTTGATCGATAGCCGCAGACTTTGTATCCCCACATATCGGCTGCATAT
GATTGTATATGCATCGAAGAGCCGAGACCTCGCCGATATACAGTCACAGAGCGAATTGCTGGGTAGTGGTAAGTTAAATCCTATGGGTCATTTTAGCAAGTGTGGTTCGTAG
AGCAAAACGGAGCACTTGAAATCGTAGTTCACGGGGTGGTAGGCGGGACCTTAGATAAACGGAATAATGTTATGATCGCGACTGGGAGATACTGCGCCGGTGCGGGTCTT
GTTTAGCCCAAAGGTAACTAGATTGTATATGCATCGAAGAGCCGAGACCTCGATTAAATCACGCGAGCTTATTCGATAGCCAAACAAGGAAGAAAACGGAGGTAAGTCTGT
ACGCACAGGGCCAGTTTCACAATACAGAAAGCCACTTGCGCACCCACACAAACAAAATTCCCAGCACCGTGTTAGAATCAGGGTCGCCAGAATTCCCTCAGTTAACGAGCT
TTTCCTCGGCTATTTTTGCCATTACACCCAACTTAATACGAGATTAGCTATACATGAGTTTCAGGCGATTTCGATCAAGCGCTGCTGCTAACCCAGATCGTGAGCGGAATCAG
TTATTGAGGGCAGAGCGCGTACTCCGGATTTCCTCACAGTCACTACTGAGCGATTCAGTAGGACGACGTGTAGGTCGCTACCGCTAGGGGACAAGAGGAAATGTTACTTAC
TTGCTCTCCCGTGGATGCGTTGCCAGGTCCAACTCTGGAATGTTAGAAGAAGGGCATCTTTATATCCGAGATTGGCGCCGAGGCAGTCGGTGATACACGGACCAGGCTGCT
GCATATCCGATATGGCTATTGTATTAAATCGCTATACGGCGGCTTGCTCTCATTGGCCAGATTGGCAGAAAGTTCAGTAAGGCAAGATTGGCCTACACGCCGGCTGGCACAG
GGATCAGGGTAACCCAGATGAGGGCTCGCAGCAACTTATGTAGCCTTTTGCCAGCTATTCTCGGAATAATTCATCGTGTCGGCAGGCTGCCGATAGCGGCGCCATAGGCGG
CGCCGAGGGGCTTAACTTACTTATTCTCTCGCCCCAGCGACGCTCTGCTATAATTTCGACTACCGCGTCCGGATGCCAGCTGGGTGCCAAGGTTGGGCTTATAAATTCAAGA
CCGGGCGGCTGGCGCCAGATTGTATATGCATCGAAGAGCCGAGACCTCGTGTGAAGAGCGGAAGTATTTTAGTTGTAAATCAAACAATCCCAATCAAAAAATCGTTATCGGC
CTGCTGCCATCTTTGTTCTTCGCCATATACGGAGATACTCAAGCGTGGGAGCAGAAGGGTCGCCAGGGGTAGTGCACTTGTCGATCCCCAGTTTTGGGTGCTTAGGCAAGT
AGGACTTCTATCCATGGGTTTGCCATTACACCCAACTTAATACGAGATTACAATCCCCAAGGTTACGGACCTCAAGCGTAGGGGATGGGAGCCGGGAAAATGAAGTAATTGC
TGGAAGAAATACTGCGAGATATTAGGGGACAAGAGGAATTGGAACGATATCCGACCCAGCTCGCCTGGCCGGTGCATTTTATTTGTATGTTCTTCTCGCCTGGAGATATCAA
GAGGCAGAAAGGTGATACTGCATATCCGTGGATGCGTTGCCAGGTCCAACTCTGGAACCGATATTAACCCTTAGGCGGATATCCGTGTGAAGACCCAGCTGCTATACGCAG

ACTATGGGTCGGGTCTCGGGAAACGTACTCTACACGCTCAAAGTAGGATTGCGCAATGAAGTAAATGTTTAAATCCCTCGACAGGAGAAACGTACTCCCATGGGTCGATCCG
GGGTGGCAAAATCAGCACCGGATGAGGGCTCGCAGCAACTTATGTAGCCTTTCTGTACGGACCTTAAGTTCACCGGGAAAAGTTGTATTGCGCACTACCAGAGTTCACCCTT
GATTCAGGCGAAGACCGAGGGTACCACGCGATTGAGAGATATCTTCTATATGAGTTTGTCGGCTGAGCGTGGGGTGGTGTGAAGAGGGTACTTAGGCACGAGATACGCCG
ATGATAAACGACGCTCGATTGTATATGCATCGAAGAGCCGAGACCTCGGCCATATGCACTTGAAATCGTAGGGGAGCTACTGAGCGATTCCCATGGGGCATAAGCCGGGTC
CGAGATAGTAACTAATTAAATTGGGAGCTATTCCGGCTCCAGTTTCAAACAACCCAAAGAGGGGAGCGGCGGTAATTGCCCAATCCCCAAGGTTTTGAGGGCGGCGGTGCA
AGTGAAACGGATGAGCGTCCTTGATTTTGCCATTACACCCAACTTAATACGAGATTATAAATCCATCGCGACTGGGAGTGGTTCGGTTCTTCTGCTATATGCGGGAGTTCCAA
GCGAACGGCAAGTGGCTTATTTGTTTAGAATTCCCGGCTATCCTCTCAGTTAGTATTTTACGAGCTTGGTTCGCGCCGATTACCTATTAACCCTTAAATTCATGAAGTTAGAGC
ACACAATATGTTATGTATGGGTCCGTGGATGCGTTGCCAGGTCCAACTCTGGAACATTTTACAGAGCGCCCCACAACTCTGACGGCTGGTTCATCGCAAAATCCTACTGAGC
GATTCTAGAGCATAACCCTCAAAATCAGGCTGCTTCATGCTGGATTGGCCTCGATCCTGCGGGAACTCTGATGCTCTCTTGAGGGGTATTTTGGCGCCATACACGCTGTTAG
ATGCGGGAACAGTCATTTGTATGATGAGGGCTCGCAGCAACTTATGTAGCCTTTAATTGCTGGGTAGTGGTAAGTATCAGGGTAACCCATGCCAGCAAGACCGATGAGTTAC
TTACTCGATAGCTGGGTGCAATCAAAAGATTGGCGCCGAGTCCATCCGCGCGAAGCGCGGATGGAGCTCGCCCCGGGGATCGACCAGTTGGTGATTTTGAACTTTTGCTTT
GCCACGGAACGGTCTGCGTTGTCGGGAAGATGCGTGATCTGATCCTTCAACTCAGCAAAAGTTCGATTTATTCAACAAAGCCGCCGTCCCGTCAAGTCAGCGTAATGCTCTG
CCAGTGTTACAACCAATTAACCAATTCTGATTAGAAAAACTCATCGAGCATCAAATGAAACTGCAATTTATTCATATCAGGATTATCAATACCATATTTTTGAAAAAGCCGTTTC
TGTAATGAAGGAGAAAACTCACCGAGGCAGTTCCATAGGATGGCAAGATCCTGGTATCGGTCTGCGATTCCGACTCGTCCAACATCAATACAACCTATTAATTTCCCCTCGT
CAAAAATAAGGTTATCAAGTGAGAAATCACCATGAGTGACGACTGAATCCGGTGAGAATGGCAAAAGCTTATGCATTTCTTTCCAGACTTGTTCAACAGGCCAGCCATTACGC
TCGTCATCAAAATCACTCGCATCAACCAAACCGTTATTCATTCGTGATTGCGCCTGAGCGAGACGAAATACGCGATCGCTGTTAAAAGGACAATTACAAACAGGAATCGAATG
CAACCGGCGCAGGAACACTGCCAGCGCATCAACAATATTTTCACCTGAATCAGGATATTCTTCTAATACCTGGAATGCTGTTTTCCCGGGGATCGCAGTGGTGAGTAACCAT
GCATCATCAGGAGTACGGATAAAATGCTTGATGGTCGGAAGAGGCATAAATTCCGTCAGCCAGTTTAGTCTGACCATCTCATCTGTAACATCATTGGCAACGCTACCTTTGCC
ATGTTTCAGAAACAACTCTGGCGCATCGGGCTTCCCATACAATCGATAGATTGTCGCACCTGATTGCCCGACATTATCGCGAGCCCATTTATACCCATATAAATCAGCATCCA
TGTTGGAATTTAATCGCGGCCTCGAGCAAGACGTTTCCCGTTGAATATGGCTCATAACACCCCTTGTATTACTGTTTATGTAAGCAGACAGTTTTATTGTTCATGATGATATAT
TTTTATCTTGTGCAATGTAACATCAGAGATTTTGAGACACAACGTGGCTTTCCCCCCCCCCCCCTGCAGGTCTCGGGCTATTCTTTTGATTTATAAGGGATTTTGCCGATTTCG
GCCTATTGGTTAAAAAATGAGCTGATTTAACAAAAATTTAACGCGAATTTTAACAAAATATTAACGTTTACAATTTAAATATTTGCTTATACAATCTTCCTGTTTTTGGGGCTTTT
CTGATTATCAACCGGGGTACATATGATTGACATGCTAGTTTTACGATTACCGTTCATCGATTCTCTTGTTTGCTCCAGACTCTCAGGCAATGACCTGATAGCCTTTGTAGACCT
CTCAAAAATAGCTACCCTCTCCGGCATGAATTTATCAGCTAGAACGGTTGAATATCATATTGATGGTGATTTGACTGTCTCCGGCCTTTCTCACCCTTTTGAATCTTTACCTAC
ACATTACTCAGGCATTGCATTTAAAATATATGAGGGTTCTAAAAATTTTTATCCTTGCGTTGAAATAAAGGCTTCTCCCGCAAAAGTATTACAGGGTCATAATGTTTTTGGTACA
ACCGATTTAGCTTTATGCTCTGAGGCTTTATTGCTTAATTTTGCTAATTCTTTGCCTTGCCTGTATGATTTATTGGATGTT
