# Supplementary material for: Folding complex DNA nanostructures from limited sets of reusable sequences
Source: Nucleic Acids Res. 2016 Apr 1;44(11):e102. doi: 10.1093/nar/gkw208 (PMC4914096; doi:10.1093/nar/gkw208)
Supplement: SUPPLEMENTARY DATA [file supp_44_11_e102__index.html]

Folding complex DNA nanostructures from limited sets of reusable sequences — SUPPLEMENTARY DATA 

# Folding complex DNA nanostructures from limited sets of reusable sequences

## SUPPLEMENTARY DATA

- SUPPLEMENTARY DATA
